# A homozygous R148W mutation in *Semaphorin 7A* causes progressive familial intrahepatic cholestasis

**DOI:** 10.15252/emmm.202114563

**Published:** 2021-09-29

**Authors:** Qiong Pan, Gang Luo, Jiaquan Qu, Sheng Chen, Xiaoxun Zhang, Nan Zhao, Jingjing Ding, Hong Yang, Mingqiao Li, Ling Li, Ying Cheng, Xuan Li, Qiaoling Xie, Qiao Li, Xueqian Zhou, Huiling Zou, Shijun Fan, Lingyun Zou, Wei Liu, Guohong Deng, Shi‐Ying Cai, James L Boyer, Jin Chai

**Affiliations:** ^1^ Cholestatic Liver Diseases Center Department of Gastroenterology Southwest Hospital Third Military Medical University (Army Medical University) Chongqing China; ^2^ Department of Pediatrics Southwest Hospital Third Military Medical University (Army Medical University) Chongqing China; ^3^ Department of Pediatrics Changsha Hospital for Maternal & Child Health Care Changsha China; ^4^ Medical Research Center Southwest Hospital Third Military Medical University (Army Medical University) Chongqing China; ^5^ Bao'an Maternal and Child Health Hospital Jinan University Shenzhen China; ^6^ Institute of Immunology Third Military Medical University (Army Medical University) Chongqing China; ^7^ Department of Infectious Diseases Southwest Hospital Third Military Medical University (Army Medical University) Chongqing China; ^8^ Department of Internal Medicine and Liver Center Yale University School of Medicine New Haven CT USA

**Keywords:** bile acid, bile salt export pump, liver injury, progressive familial intrahepatic cholestasis, semaphorin 7A, Digestive System, Genetics, Gene Therapy & Genetic Disease

## Abstract

Semaphorin 7A (SEMA7A) is a membrane‐bound protein that involves axon growth and other biological processes. *SEMA7A* mutations are associated with vertebral fracture and Kallmann syndrome. Here, we report a case with a mutation in *SEMA7A* that displays familial cholestasis. WGS reveals a *SEMA7A* ^R148W^ homozygous mutation in a female child with elevated levels of serum ALT, AST, and total bile acid (TBA) of unknown etiology. This patient also carried a *SLC10A1* ^S267F^ allele, but *Slc10a1* ^S267F^ homozygous mice exhibited normal liver function. Similar to the child, *Sema7a* ^R145W^ homozygous mice displayed elevated levels of serum ALT, AST, and TBA. Remarkably, liver histology and LC‐MS/MS analyses exhibited hepatocyte hydropic degeneration and increased liver bile acid (BA) levels in *Sema7a* ^R145W^ homozygous mice. Further mechanistic studies demonstrated that *Sema7a* ^R145W^ mutation reduced the expression of canalicular membrane BA transporters, bile salt export pump (Bsep), and multidrug resistance‐associated protein‐2 (Mrp2), causing intrahepatic cholestasis in mice. Administration with ursodeoxycholic acid and a dietary supplement glutathione improved liver function in the child. Therefore, *Sema7a* ^R145W^ homozygous mutation causes intrahepatic cholestasis by reducing hepatic Bsep and Mrp2 expression.

## Graphical abstract

A new type of progressive familial intrahepatic cholestasis (PFIC) was caused by the homozygous R148W mutation in SEMA7A. Preliminary mechanistic studies revealed that the mutation reduced hepatic expression of canalicular membrane bile acid (BA) efflux transporters Bsep and Mrp2, resulting in intrahepatic cholestasis.

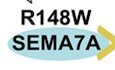

**The paper explained**

**Problem**

Semaphorin 7A (SEMA7A) is crucial for axonal growth, T‐cell responses, and other biological processes. Mutations in *SEMA7A* are associated with increased risks of vertebral fracture and Kallmann syndrome. However, whether *SEMA7A* mutations involve in liver diseases remains unknown.

**Results**

We identified a homozygous R148W mutation in *SEMA7A* in a female child, who developed cholestatic liver injury with elevated levels of serum ALT, AST, and total bile acid (TBA) without known etiology. Although this patient carried *SLC10A1* ^S267F^ allele, *Slc10a1* ^S267F^ homozygous mice exhibited normal liver function. In contrast, *Sema7a* ^R145W^ homozygous mice displayed elevated levels of serum ALT, AST, and TBA, recapitulating the patient's phenotypes. The homozygous mice also presented the accumulation of conjugated bile acid in the liver and reduced protein expression of canalicular membrane BA transporters, bile salt export pump (BSEP), and multidrug resistance‐associated protein‐2 (MRP2). Administration with ursodeoxycholic acid (UDCA) and a dietary supplement glutathione (GSH) improved liver function in the child.

**Impact**

Our findings uncover the clinical features of a novel form of progressive familial intrahepatic cholestasis (PFIC), caused by the *SEMA7A* ^R148W^ homozygous mutation. Administration with UDCA and GSH may be an effective therapy for this new type of PFIC.

## Introduction

Bile acid (BA) transporters are crucial for maintaining BA homeostasis by driving bile flow (Boyer, 2013; Boyer & Soroka, 2021). The deficiency or reduction of canalicular membrane BA efflux transporters, including bile salt export pump (BSEP/ABCB11) and multidrug resistance‐associated protein‐2 (MRP2/ABCC2), can impair bile flow in the liver (Boyer, 2013; Boyer & Soroka, 2021). Impaired bile flow leads to cholestasis, resulting in liver injury characterized by elevated levels of bile acid (BA) and liver enzymes in the serum and liver (Wagner & Trauner, 2016; Pan *et al*, 2018; Amirneni *et al*, 2020). Cholestasis is attributed to multiple pathogenic factors, including hereditary and acquired impairment of bile formation (Wagner & Trauner, 2016). Several cholestatic disorders have been characterized as mutations in genes involved in bile formation, including ATP8B1 (a phospholipid flippase), BSEP, multidrug resistance protein 3 (MDR3/ABCB4), tight junction protein‐2 (TJP2), farnesoid X receptor (FXR/NR1H4), and myosin VB (MYO5B), leading to progressive familial intrahepatic cholestasis (PFIC) 1–6, respectively (Amirneni *et al*, 2020). Here, we present a mutation in *Semaphorin 7A* (*SEMA7A*) for an additional cause of PFIC.

Semaphorins are extracellular signaling proteins mediated through their membrane receptors plexins and integrins, contributing to tissue morphogenesis and homeostasis (Alto & Terman, 2017). Semaphorin 7A (SEMA7A), also known as the John Milton Hagen (JMH) antigen or CD108, is the only membrane‐bound semaphoring linked to glycophosphatidylinositol (GPI) (Yamada *et al*, 1999). SEMA7A is expressed in multiple tissues, including the liver, intestine, lung, bone, and brain (Yamada *et al*, 1999; Liu *et al*, 2010; Song *et al*, 2021). It is crucial for axon growth, immune cell activation, pulmonary fibrosis, cancer metastasis, and other biological processes (Yamada *et al*, 1999; Suzuki *et al*, 2007; Liu *et al*, 2010; Song *et al*, 2021). Mutations in *SEMA7A* are associated with decreased bone mineral density (BMD) and Kallmann syndrome in humans (Koh *et al*, 2006; Zhao *et al*, 2020). However, the functional role of *SEMA7A* mutations in human liver diseases, including cholestasis, remains unclear.

In this report, we identified a female infant patient with a homozygous mutation in *SEMA7A* (p.R148W) and elevated levels of serum alanine transaminase (ALT), aspartate transaminase (AST), and total bile acids (TBA). A genetic mouse model with the homozygous mutation recapitulated the clinical phenotypes. Administration with ursodeoxycholic acid (UDCA) and a dietary supplement glutathione (GSH) improved liver function in the child. Our findings describe the clinical features and possible therapy for a novel form of PFIC, caused by the *SEMA7A* ^R148W^ homozygous mutation in humans.

## Results

### Clinical features of the child patient and her family

**Figure 1:**
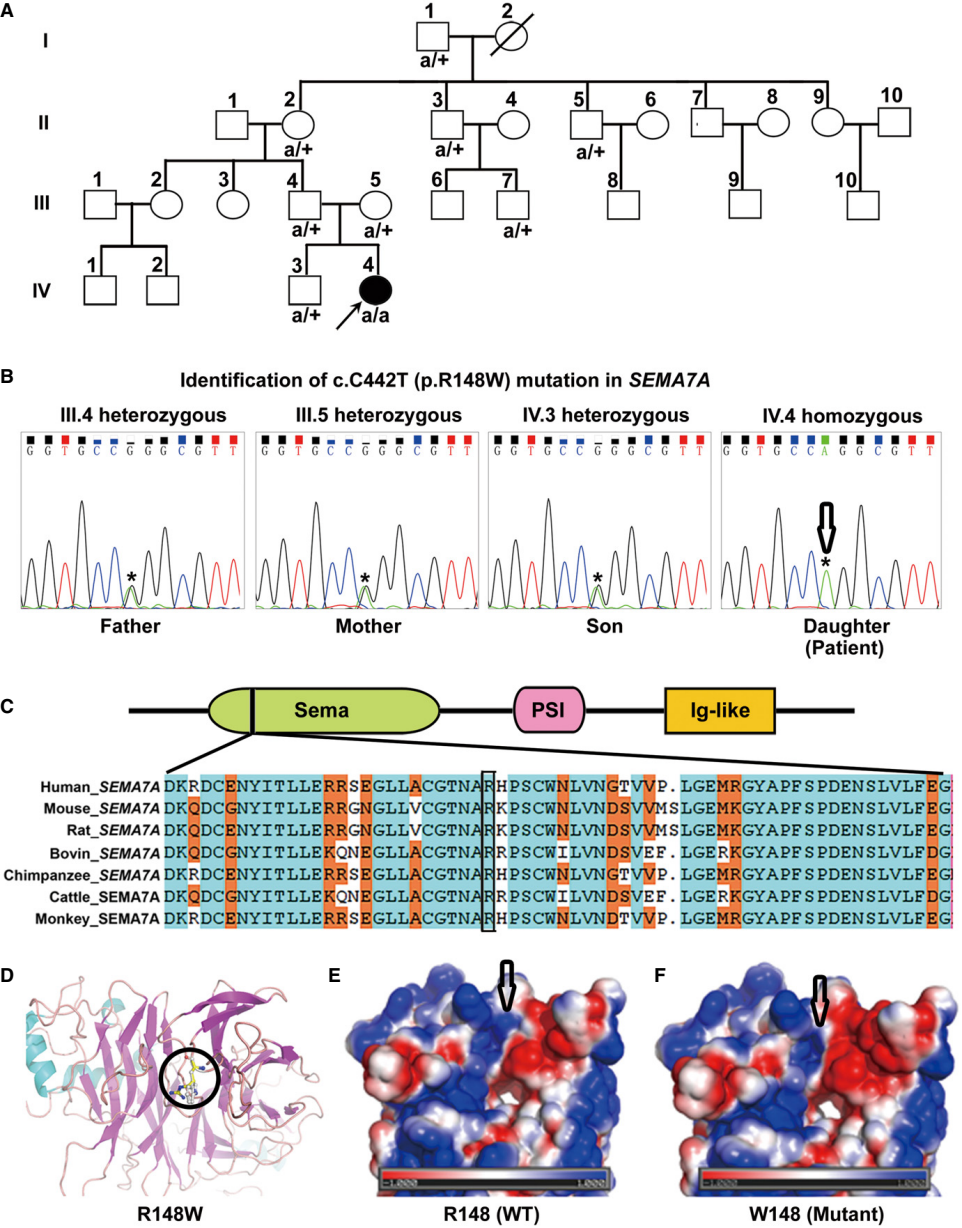
Identification of a new case with a rare homozygous p.R148W mutation in *SEMA7A* APedigrees of the studied family. A child patient (Patient: IV.4) with elevated serum ALT, AST, and TBA levels and her nuclear family members (III.4, III.5, and IV.3) were evaluated by WGS analysis. Mutated alleles are depicted as “a” for *SEMA7A*. Square symbols: male; circles: female; solid: the patient. The reference alleles are depicted by a plus sign. The arrow points to the proband.BThe homozygous p.R148W mutation in *SEMA7A* was further confirmed by Sanger sequencing.CThe protein domain architecture of SEMA7A and conservation of the R148 position in Vertebrata.DStructures of WT SEMA7A and R148W mutant show local conformational changes, as a result of the replacement of an arginine (carbon atoms in yellow) with a tryptophan (pale) at position 148, which is highlighted in ball‐and‐stick models.E, FThe surface electrostatic potential map of the WT and R148W mutant proteins, respectively, in which positively and negatively charged resides are expressed in blue and red, respectively, and non‐polar residues are denoted in white. Source data are available online for this figure.

**Figure EV1:**
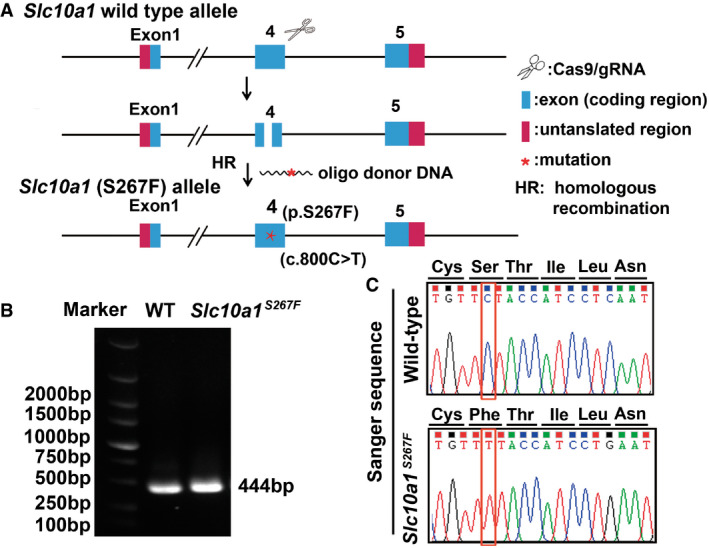
Generation and characterization of *Slc10a1* ^S267F^ mutant mice Schematic diagram for the generation of *Slc10a1* ^S267F^ mutant mice.Genotyping of *Slc10a1* ^S267F^ mutant mice. Genomic DNA was extracted from mouse tails, and the specific region covering the mutation was amplified by PCR.Identification of *Slc10a1* ^S267F^ homozygous mice by Sanger sequencing analysis. Source data are available online for this figure.

An infant patient (Patient IV.4, Fig 1A) presented with elevated levels of serum ALT, AST, and TBA, but normal levels of serum alkaline phosphatase (ALP), gamma‐glutamyl transferase (GGT), direct bilirubin (DBIL), and total bilirubin (TBIL) (AppendixTable S1). Further laboratory and radiological examinations excluded any of the known liver diseases (Appendix Fig S1 and Appendix Tables S4–S8), leading to a speculation of mutations in genes involved in bile formation, such as those PFIC genes (Amirneni *et al*, 2020). However, the whole‐genome sequencing (WGS) analysis of the studied family members did not show any mutation in known PFIC genes (i.e., ATP8B1, BSEP, MDR3, TJP2, FXR, and MYO5B), whereas a homozygous mutation (p.S267F) in sodium/taurocholate cotransporting polypeptide (NTCP/*SLC10A1*) was found in the patient. NTCP is a BA uptake transporter at the basolateral membrane of hepatocytes, and its loss‐of‐function mutation (p.R252H) causes hypercholanemia without liver injury (Vaz *et al*, 2015). Sanger sequencing analysis evidenced the homozygous p.S267F mutation in *SLC10A1* (Appendix Fig S2), with an allele frequency of 8–12% in populations of Southern China, which is significantly associated with hypercholanemia in Han Chinese (Liu *et al*, 2017). To determine whether the *SLC10A1* ^S267F^ mutation was important for her liver injury, we generated *Slc10a1* ^S267F^ homozygous mice, a point mutation of c.800C>T (Ser267Phe) in murine *Slc10a1* (Fig EV1). The *Slc10a1* ^S267F^ homozygous mice exhibited normal levels of serum ALT, AST, and TBA (Appendix Table S2) and liver histology. Therefore, the *SLC10A1* ^S267F^ homozygous mutation was unlike to cause liver injury and hypercholanemia in our patient, suggesting that her abnormal liver function may be attributed to other genetic mutations.

### SEMA7A is a candidate susceptibility gene

To identify potential causative genetic factors, we screened the WGS data using a recessive inheritance model (Appendix Tables [Link], [Link] and [Link], [Link]), a compound heterozygous inheritance model (Appendix Tables S11 and S12 (Schulten *et al*, 2016)), a de novo model (Appendix Table S13), and a dominant inheritance model (Appendix Table S14). The nonsynonymous, rare, and predicted to be damaging variants were further filtered to assess for further evidence of disease causation (Appendix Tables S9–S14). Notably, a predicted missense variant in *SEMA7A* gene (homozygous p.R148W) was identified and was prioritized for further analysis (Appendix Tables S9–S14). Sanger sequencing confirmed that the patient (Patient IV.4) had homozygous variant of c.C442T in *SEMA7A*, a missense mutation of p.R148W (Fig 1B and Appendix Fig S3).

The R148 in SEMA7A is conserved in vertebrata (Fig 1C) and is located in a β‐hairpin loop at an edge of the second β‐sheet (Liu *et al*, 2010). This amino acid is partially exposed to solvent with slight restraints from two neighboring β‐hairpin loops. Structure modeling indicated that the replacement of R148 with a Trp residue changed its local conformation due to the bulky indole side chain of tryptophan (Fig 1D) and altered the electrostatic property of local milieu by replacing a positively charged amino acid with an aromatic residue (Fig 1E and F). These findings suggest that the mutation may affect the function of the SEMA7A protein.

### Sema7a^R145W^ homozygous mutation induces elevated serum ALT, AST, and TBA levels in mice

**Figure EV2:**
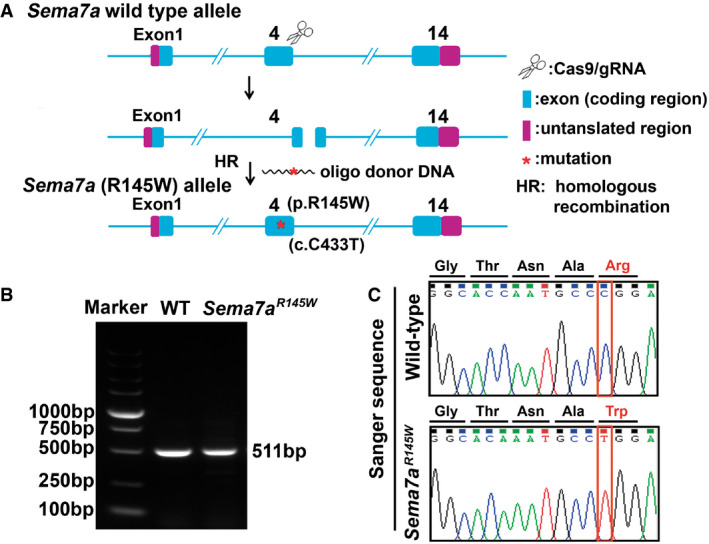
Generation and characterization of *Sema7a* ^R145W^ mutant mice Schematic diagram for the generation of *Sema7a* ^R145W^ mutant mice.Genotyping *Sema7a* ^R145W^ mutant mice. Genomic DNA was extracted from mouse tails, and the specific region was amplified by PCR.Identification of *Sema7a* ^R145W^ homozygous mice by Sanger sequencing analysis. Source data are available online for this figure.

**Figure EV3:**
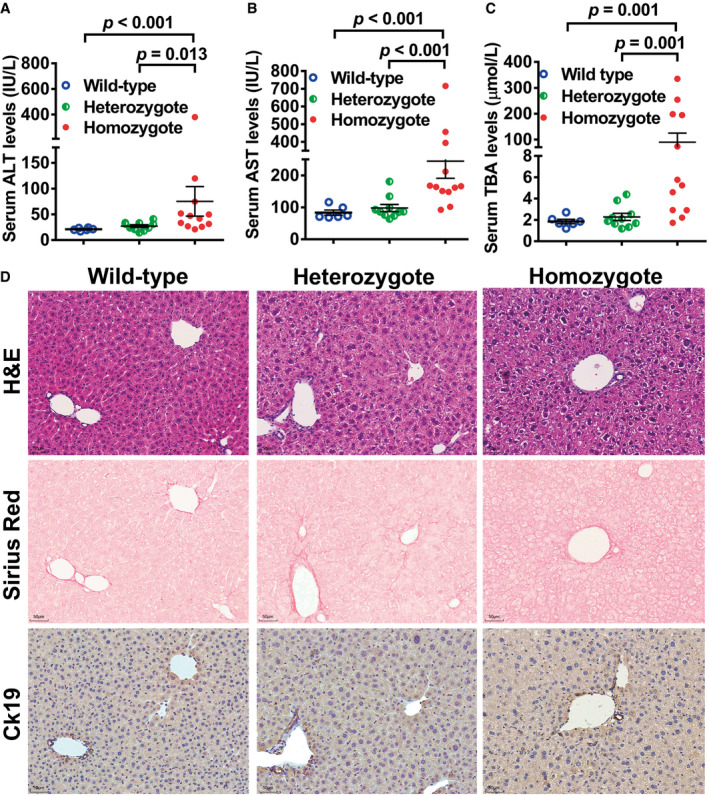
A *Sema7a* ^R145W^ homozygous mutation causes the elevated serum ALT, AST, and TBA levels and remarkable hydropic degeneration in mouse livers A–CSerum ALT, AST, and TBA in *Sema7a* ^R145W^ WT (*n* = 6, two male/four female), heterozygous (*n* = 10, six male/four female) and homozygous mice (*n* = 12, seven male/five female). Data are shown as means ± SD.DRepresentative images of H&E staining, *Sirius Red* staining, and IHC analysis of CK19 expression in WT, *Sema7a* ^R145W^ heterozygous and homozygous mice. The *Sema7a* ^R145W^ homozygous mutation caused significant liver injury with elevated levels of serum ALT, AST, and TBA and striking hydropic degeneration in hepatocytes. Scale bar, 50 µm. Data information: The data were analyzed by the Mann–Whitney *U*‐test. Source data are available online for this figure.

Similar to our patient with the homozygous *SEMA7A* ^R148W^ mutation, *Sema7a* ^R145W^ homozygous mice (Fig EV2) displayed elevated levels of serum ALT, AST, and TBA in both 4 and 8 weeks of ages, compared to the age‐matched WT and heterozygotes (Fig EV3A–C and Table 1 & Appendix Table S15). These homozygotes also had slightly elevated levels of serum TBIL and DBIL (Table 1). Moreover, liver histological assessments in *Sema7a* ^R145W^ homozygotes exhibited striking hydropic degeneration in hepatocytes, substantially higher than that of WT and heterozygotes (Fig EV3D). Together, the *Sema7a* ^R145W^ homozygous mutation in mice caused liver injury, characterized by elevated levels of serum ALT, AST, and TBA and striking hydropic degeneration in hepatocytes.

**Table 1:** Serum biochemistry of *Sema7a* ^R145W^ mutant mice (8‐week‐old).

	Wild type (*n* = 6)	Heterozygote (*n* = 10)	Homozygote (*n* = 12)
Gender (male/female)	2/4	6/4	7/5
Serum ALT (IU/l)	21.00 ± 2.69	27.14 ± 8.24	75.23 ± 99.65^a^, ^b^
Serum AST (IU/l)	83.26 ± 19.91	97.81 ± 34.87	244.40 ± 185.40^a^, ^b^
Serum ALP (IU/l)	98.80 ± 57.10	86.45 ± 36.04	93.91 ± 82.04
Serum TBA (μmol/l)	1.86 ± 0.51	2.28 ± 1.05	90.03 ± 121.39^a^, ^b^
Serum TBIL (μmol/l)	0.98 ± 1.15	3.65 ± 7.64	7.28 ± 5.18^a^, ^b^
Serum DBIL (μmol/l)	0.39 ± 0.61	2.91 ± 6.59	4.63 ± 4.38^a^

Values are mean ± SD.

ALT, alanine aminotransferase; AST, aspartate aminotransferase; ALP, alkaline phosphatase; TBA, total bile acids; TBIL, total bilirubin; DBIL, direct bilirubin.

^a^ *P* < 0.05 versus the WT mice.

^b^ *P* < 0.05 versus the heterozygous mutant mice. The data were analyzed by the independent‐samples Student's *t*‐test or the Mann–Whitney *U*‐test when applicable.

### Sema7a^R145W^ homozygous mutation causes intrahepatic BA accumulation by reducing the expression of canalicular membrane BA efflux transporters Bsep and Mrp2

**Figure 2:**
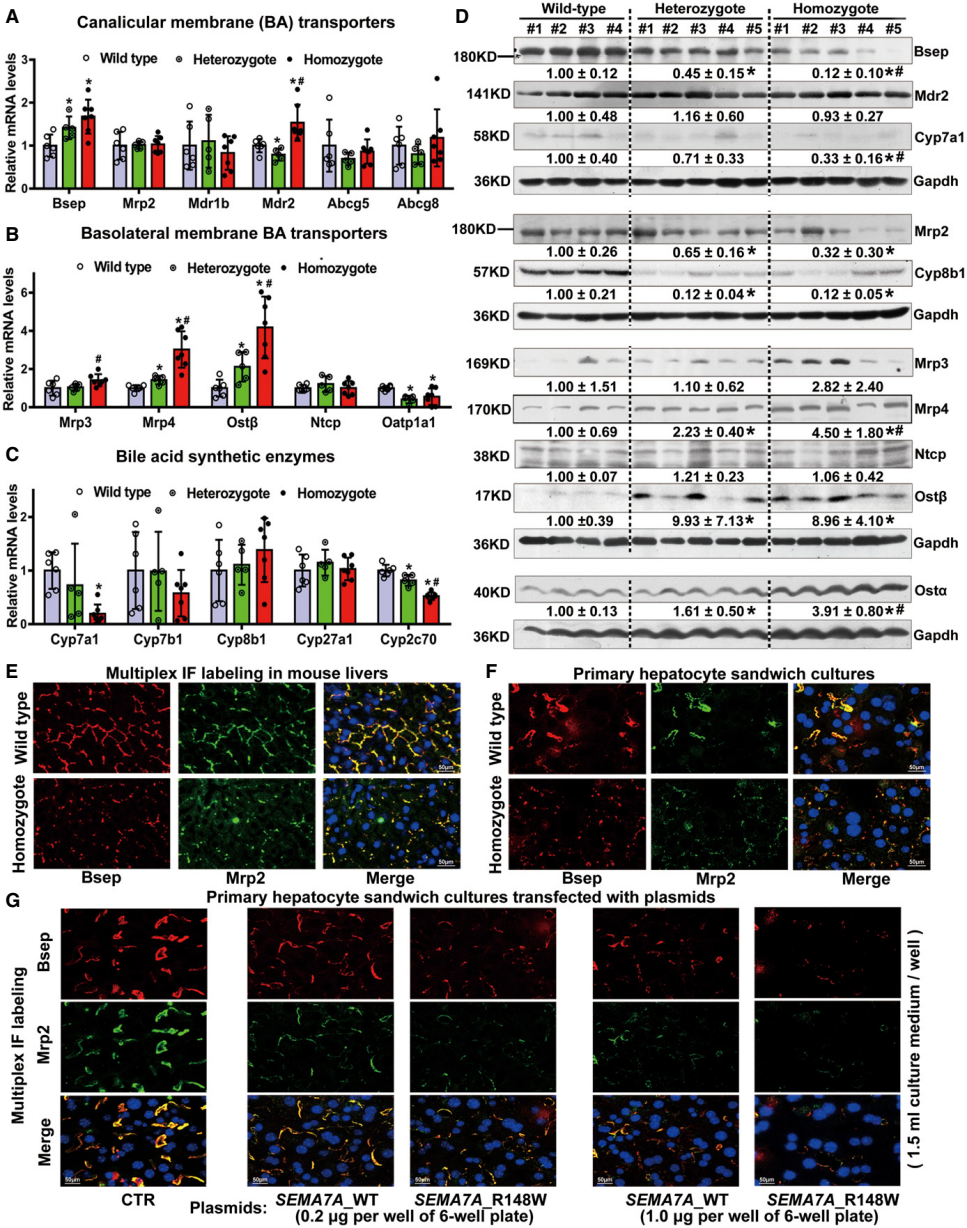
The R145W mutation in *Sema7a* markedly reduces the levels of canalicular membrane bile acid (BA) efflux transporters, Bsep and Mrp2 in hepatocytes A–CThe relative levels of (A) canalicular membrane (BA) transporter Bsep, Mrp2, Mdr1b, Mdr2, Abcg5, and Abcg8 mRNA transcripts; (B) basolateral membrane BA transporter Mrp3, Mrp4, Ostα/β, Ntcp, and Oatp1a1 mRNA transcripts; and (C) BA synthetic enzyme Cyp7a1, Cyp7b1, Cyp8b1, Cyp27a1, and Cyp2c70 mRNA transcripts. Wild type, wild‐type mice, *n* = 6; Heterozygote, *Sema7a* ^R145W^ heterozygous mice, *n* = 5; Homozygote, *Sema7a* ^R145W^ homozygous mice, *n* = 7. Data are shown as means ± SD. *P < 0.05 versus the WT mice, ^#^ *P* < 0.05 versus the heterozygous mutant mice. The data were analyzed by the independent‐samples Student's *t*‐test and the Mann–Whitney *U*‐test.DWestern blot analysis of the relative levels of Bsep, Mrp2, Mdr2, Mrp3, Mrp4, Ostα/β, Ntcp, Cyp7a1, and Cyp8b1 protein expression in mouse livers. Contrary to the mRNA alterations, the levels of canalicular membrane Bsep and Mrp2 proteins were dramatically reduced in homozygous mouse livers, suggesting that the decreased proteins might be mediated by post‐translational regulation in the livers of *Sema7a* ^R145W^ mutant mice. Wild type, wild‐type mice, *n* = 4; Heterozygote, *Sema7a* ^R145W^ heterozygous mice, *n* = 5; Homozygote, *Sema7a* ^R145W^ homozygous mice, *n* = 5. *P < 0.05 versus the WT mice, ^#^ *P* < 0.05 versus the heterozygous mutant mice. The data were analyzed by the independent‐samples Student's *t*‐test and the Mann–Whitney *U*‐test.E, FMultiplex IF analyses of Bsep and Mrp2 expression in the livers (E) and primary hepatocytes in sandwich cultures (F) from WT and homozygous mice. Scale bar, 50 µm.GMultiplex IF analyses revealed that transfection with the *SEMA7A*_WT or *SEMA7A*_R148W construct decreased the levels of canalicular membrane Bsep and Mrp2 expression in mouse primary hepatocytes in collagen sandwich cultures in a dose‐dependent manner (0, 0.2, 1.0 μg per well of six‐well plate; 1.5 ml culture medium per well). Scale bar, 50 µm. Together, the *Sema7a* ^R145W^ mutation can reduce hepatic Bsep and Mrp2 protein expression and lead to intrahepatic BA accumulation and cholestasis (Appendix Table S16). Subsequently, cholestasis triggered an adaptive response in the liver by down‐regulating the expression of BA synthetic enzymes of Cyp7a1 and Cyp8b1 and up‐regulating the expression of BA efflux transporters Mrp3, Mrp4, and Ostα/β. Source data are available online for this figure.

LC‐MS/MS analysis of mouse liver extracts indicated that hepatic levels of major murine conjugated BAs, taurocholic acid (TCA), tauromuricholic acid (TMCA), taurochenodeoxycholic acid (TCDCA), and taurohyodeoxycholic acid (THDCA), were markedly higher in *Sema7a* ^R145W^ homozygous mice than WT mice (*P* < 0.05, Appendix Table S16), hallmarks of impaired hepatic excretion of BA. Furthermore, hepatic gene expression revealed that the mRNA transcripts and/or protein expression of BA synthetic enzymes Cyp7a1, Cyp8b1, and Cyp2c70 decreased while BA efflux transporters Mrp3, Mrp4, and Ostα/β each increased in homozygous mice, when compared to WT controls (Fig 2A–D). Most importantly, the levels of hepatic Bsep and Mrp2 protein expression in the *Sema7a* ^R145W^ homozygous mice were markedly reduced whereas their mRNA transcripts were not down‐regulated (Fig 2A and D). Multiplex immunofluorescent (IF) analysis further revealed that reduced levels of Bsep and Mrp2 expression were observed in the livers and primary hepatocytes in sandwich cultures from *Sema7a* ^R145W^ homozygous mice (Fig 2E and F), suggesting that these two proteins decreased by the post‐translational regulation in the livers of *Sema7a* ^R145W^ mutant mice. Interestingly, the levels of canalicular membrane Bsep and Mrp2 proteins were also markedly reduced in primary mouse hepatocytes in collagen sandwich cultures following transfection with *SEMA7A*_WT or *SEMA7A*_R148W construct in a dose‐dependent manner (Fig 2G). Furthermore, when the same amount of DNA was transfected into these cells, we observed greater reduction in *SEMA7A*_R148W construct‐transfected cells than in *SEMA7A*_WT construct‐transfected cells (Fig 2G). Together, these findings indicated that the *Sema7a* ^R145W^ mutation reduced the protein expression of hepatic Bsep and Mrp2, resulting in intrahepatic cholestasis.

### Administration with UDCA and a dietary supplement GSH corrects abnormal liver function in the child patient

**Figure 3:**
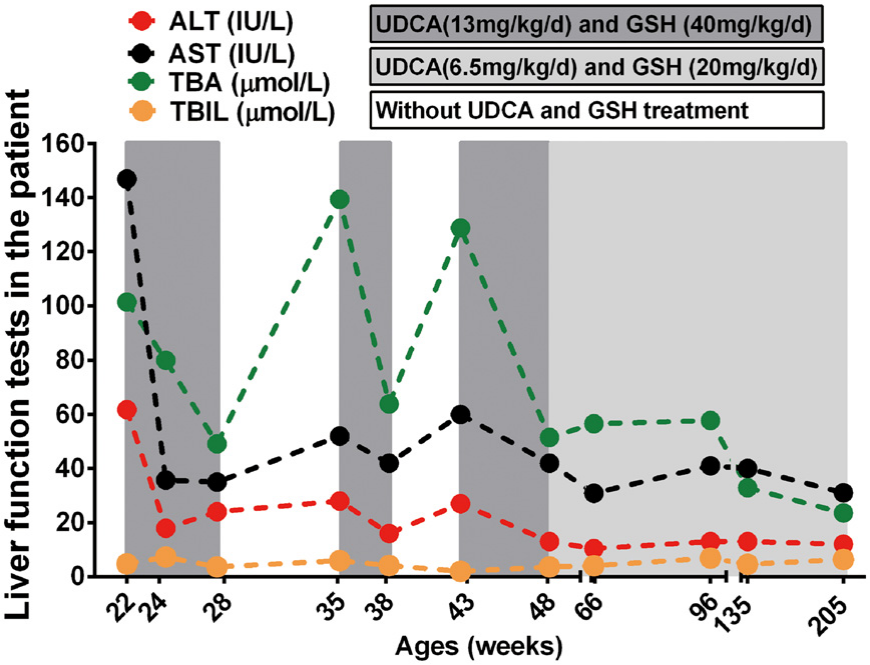
Administration of UDCA and GSH improved liver function in the child patient with this new type of PFIC Treatment with UDCA (13 mg/kg/day) and GSH (40 mg/kg/day) for 2 weeks restored normal liver function in the child. However, when the treatment was ceased the levels of serum ALT, AST, and TBA returned to abnormally high levels in the patient. Interestingly, treatment with half of the dose of UDCA and GSH also maintained the normal levels of serum ALT and AST and reduced serum TBA in the child patient. Source data are available online for this figure.

Initially, the child patient was administrated with UDCA (13 mg/kg/day) and GSH (40 mg/kg/day) as empiric therapy. Strikingly, as shown in Fig 3, treatment with UDCA and GSH for 2 weeks significantly restored the levels of serum ALT and AST to normal and reduced the levels of serum TBA in the patient. However, when the treatment was stopped, her serum ALT, AST, and TBA levels returned to be abnormally higher (Fig 3). Interestingly, treatment with half of the dose was also able to maintain normal levels of serum ALT and AST, and reduced serum TBA levels (Fig 3). Together, our data demonstrated that treatment with both UDCA and GSH significantly improved abnormal liver function in the patient with this new type of PFIC.

## Discussion

Here, we are the first to report a case of familial cholestasis caused by a homozygous p.R148W mutation in *SEMA7A* and uncover its preliminary pathophysiologic mechanism in a mouse model. This new genetic cholestatic disorder is characterized by elevated levels of serum ALT, AST, and TBA (Table 1 and Appendix Table S15). Administration with UDCA and GSH effectively improved liver function in the child patient (Fig 3). Analyses of the mouse model displayed hepatocyte hydropic degeneration (Fig EV3D), reduced protein expression of canalicular BA transporters BSEP and MRP2 (Fig 2D–F), and intrahepatic BA accumulation (Appendix Table S16).

**Figure EV4:**
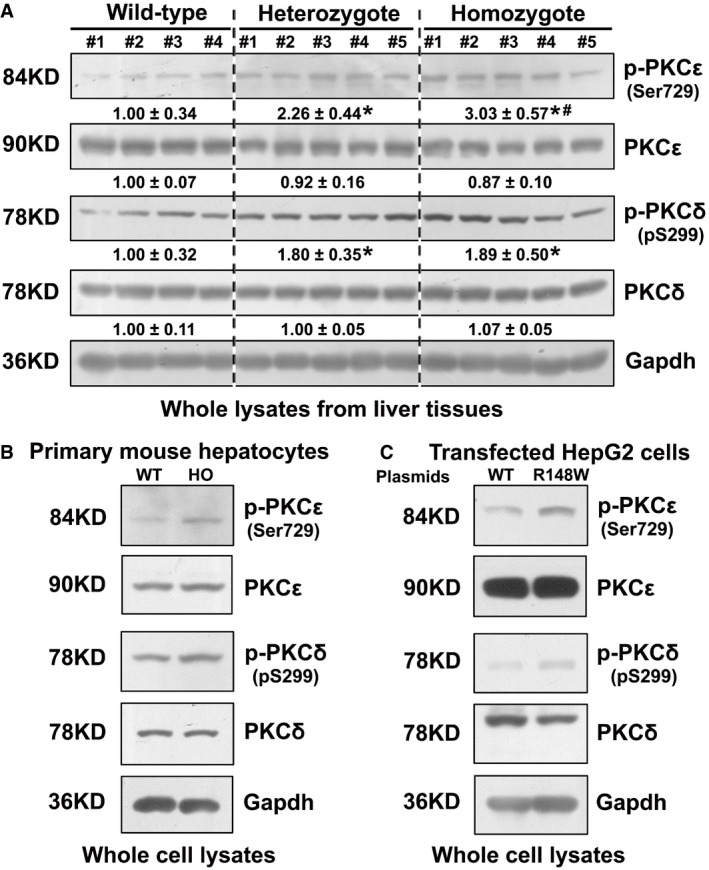
The *Sema7a* ^R145W^ mutation increases PKCδ/ε phosphorylation in hepatocytes AThe levels of hepatic PKCδ/ε phosphorylation were significantly higher in *Sema7a* ^R145W^ homozygous mice than in heterozygous mice and WT mice. Wild type, wild‐type mice, *n* = 4; Heterozygote, *Sema7a* ^R145W^ heterozygous mice, *n* = 5; homozygote, *Sema7a* ^R145W^ homozygous mice, *n* = 5. *P < 0.05 versus the WT mice, ^#^ *P* < 0.05 versus the heterozygous mutant mice. The data were analyzed by the independent‐samples Student's *t*‐test and the Mann–Whitney *U*‐test.B, CFurthermore, *Sema7a* ^R145W^ (human R148W) homozygous mutation increased PKCδ/ε phosphorylation in *Sema7a* ^R145W^ mouse primary hepatocytes (B) and human HepG2 cells following transfection with *SEMA7A*_R148W construct (C). Source data are available online for this figure.

SEMA7A has been associated with multiple biological processes and diseases (Yamada *et al*, 1999; Alto & Terman, 2017; Körner *et al*, 2021; Song *et al*, 2021). However, the detailed molecular events remain elusive. Here, we found that *SEMA7A* ^R148W^ mutation led to cholestatic liver injury in a patient and its mouse model (Table 1 and Appendix Table S1). Characterization of *Sema7a* ^R145W^ homozygous mouse livers and primary hepatocytes indicated that this mutation increased PKCδ/ε phosphorylation (Fig EV4A and B) and reduced expression of canalicular membrane Bsep and Mrp2 (Fig 2D–F). Cell culture experiments for *Sema7a* ^R145W^ primary mouse hepatocytes and human HepG2 cells transfected with *SEMA7A_*R148W construct confirmed the increase in PKCδ/ε phosphorylation by this mutation (Fig EV4B and C). Furthermore, over‐expression of *SEMA7A*_WT or *SEMA7A*_R148W protein markedly decreased canalicular Bsep and Mrp2 proteins in primary mouse hepatocytes in sandwich cultures in a dose‐dependent manner, particularly after over‐expression of the mutant protein (Fig 2G). Hence, *Sema7a* ^R145W^ mutation was like to be a gain‐of‐function mutation to reduce canalicular Bsep and Mrp2 expression in hepatocytes by increasing PKC activities, as PKC activation reduces canalicular Bsep and Mrp2 proteins in cholestatic hepatocytes (Kubitz *et al*, 2004; Perez *et al*, 2006; Crocenzi *et al*, 2008; Chai *et al*, 2015). Moreover, Sema7a is crucial for mediating inflammatory responses (Ghofrani *et al*, 2019; Körner *et al*, 2021; Song *et al*, 2021). Our data demonstrated that *Sema7a* ^R145W^ homozygous mutation in mice caused the accumulation of intrahepatic BA (Appendix Table S16), which can trigger inflammatory response to induce cholestatic liver injury (Cai & Boyer, 2021). These suggested that this mutation might also promote liver inflammation. In agree with above views, administration of UDCA supplied with GSH to the child patient significantly improved her liver function (Fig 3), because this treatment can recover the expression/function of Bsep and Mrp2 and mitigate hepatic inflammation in cholestasis (Kagawa *et al*, 2014; Gonzales *et al*, 2015). Nevertheless, the detail molecular mechanisms for this new PFIC remain to be extensively investigated in the future.

A recent study has reported that a loss‐of‐function mutation (p.R252H) in *SLC10A1* causes a hypercholanemia without liver injury (Vaz *et al*, 2015). In the current study, we also identified a homozygous p.S267F mutation in *SLC10A1* (Appendix Fig S2), an allele frequency of 8–12% in Southern China population (Liu *et al*, 2017), which may also contribute to elevated levels of serum TBA in the child patient. However, *Slc10a1* ^S267F^ homozygous mice displayed normal levels of serum TBA, ALT, and AST (Appendix Table S2). These data clearly demonstrated that the *Slc10a1* ^S267F^ homozygous mutation did not cause hypercholanemia and liver injury in mice. More importantly, the *Sema7a* ^R145W^ homozygous mice displayed elevated levels of serum ALT, AST, and TBA (Table 1), mimicking the clinical characteristics of the child patient (Appendix Table S1), and exhibited hydropic degeneration in hepatocytes and excessive accumulation of BA in the liver (Fig EV3C and Appendix Table S16). Therefore, we conclude that the homozygous *SEMA7A* ^R148W^ mutation is the cause of liver function abnormalities in the child patient, although we were not able to obtain liver specimen from this child patient for further analyses. In addition, pruritus is a common feature in many cholestatic patients, including PFIC1–3 patients (Chen *et al*, 2018). El‐Guindi *et al* (2016) reported a 35.3% incidence of pruritus in a PFIC2 cohort of 17 patients. However, we did not observe pruritus in the child patient. Whether this new PFIC is associated with pruritus, additional patients need to be further identified and characterized.

In conclusion, we reported a new type of PFIC, caused by a homozygous *SEMA7A* ^R148W^ mutation, and revealed its clinical features and preliminary pathogenic mechanisms in mice. These findings associate *SEMA7A* mutations with a novel form of PFIC. Administration with UDCA supplied with GSH was an effective therapy for this disease.

## Materials and Methods

### The patient and members of her family

The female patient (IV.4, Fig 1) was born at a gestational age of 40 weeks and 5 days with a body weight of 3,500 *g* through an ordinary pregnancy. Apgar scores were all 10 at 1, 5, and 10 min postbirth, respectively. She was the second child of Chinese Han parents (III.4 and III.5, Fig 1) in Liuyang city (Hunan, China) and had one healthy brother (IV.3, Fig 1). She displayed prolonged jaundice and flatulence during the first 2 weeks postbirth and was administered in a local hospital. After clearance of jaundice, she did not manifest pruritus or steatorrhea. However, her liver functional tests exhibited abnormally high levels of serum ALT, AST, and TBA in her subsequent visits (Appendix Table S1). Abdominal ultrasonography and MRI revealed normal liver size and parenchyma without biliary tract obstruction or other abnormalities (Appendix Fig S1). The biochemical characteristics of the patient and her family members are shown in Appendix Table S1 and Appendix Tables S3–S8. Further laboratory and radiological examinations excluded autoimmune hepatitis, viral hepatitis, infections with cytomegalovirus and Epstein–Barr virus, liver cirrhosis, Wilson's disease, α1‐antitrypsin deficiency, drug‐induced liver injury, alcoholic hepatitis, intra‐ductal obstruction or abnormalities, extra‐biliary malignancy, gastrointestinal diseases, hematological diseases, and other known liver diseases (Appendix Fig S1 and Appendix Tables S4–S8). Her neurological examinations were normal and her motor and cognitive developments progressed normally. She had a neonatal behavioral neurological assessment (NBNA) score of 38. This study was carried out in accordance with the Declaration of Helsinki (2013) of the World Medical Association and the principles set out in the Department of Health and Human Service Belmont Report. The study protocol was reviewed and approved by the Institutional Ethics Review Board of the Southwest Hospital, Chongqing, China. Written informed consent was obtained from individual subjects and their parents.

### Whole‐genome sequencing

Peripheral blood mononuclear cells (PBMCs) were isolated from the patient and members of her nuclear family (III.4, III.5, IV.3, and Patient IV.4). Their genomic DNA was extracted using standard protocols. The DNA samples were sonicated and separated by electrophoresis. The resulting DNA fragments were used for establishment of DNA sequencing libraries using the TruSeq DNA LT Sample Prep kit (Illumina, San Diego, CA, USA). The libraries were subjected to massively parallel sequencing in the Illumina HiSeq X (Shanghai OE Biotech, China) to generate 150bp paired‐end reads.

### WGS bioinformatics

The quality of WGS data was controlled using NGS QC Tookit (Patel & Jain, 2012). The sequencing reads were aligned to the reference genome (GRCh37/hg19) using Burrows–Wheeler Aligner (BWA) to create compressed sequence alignment/map (SAM) files, which were transferred by the SAMtools (Li *et al*, 2009) into compressed Binary Alignment Maps (BAM) files and used for sorting and indexing. To ensure accurate SNP and InDel calling, we used the option of HaplotypeCaller of the Genome Analysis Tookit (GATK) (McKenna *et al*, 2010; DePristo *et al*, 2011) following the recommended best practices. The duplicate reads were removed using Picard tools (http://broadinstitute.github.io/picard). The annotation for these variants was analyzed by ANNOVAR (Wang *et al*, 2010). PLINK (Abecasis *et al*, 2010) was used to calculate summary statistics for each subject for control of the variants. These included the total numbers of variants, heterozygous genotypes, genotyping rate, singleton variants, and transition/transversion ratio.

### Analysis of variants from WGS

The ANNOVAR‐annotated files of the studied family were processed following the predicted mode of disease inheritance. The potential disease‐related variants in each mode were identified by a series of filtering steps based on the effect of variant on the gene: minor allele frequency (Merico *et al*, 2015) using the variant frequencies of multiple control databases (1,000 genomes (Abecasis *et al*, 2010), ExAC (Lek *et al*, 2016), ESP6500 (Fu *et al*, 2013)) and pathogenicity predictions of multiple bioinformatic algorithms (i.e., Polyphen‐2 (Adzhubei *et al*, 2010), SIFT (Vaser *et al*, 2016), MutationTaster (Schwarz *et al*, 2014)). Manual verification of variant calls was performed by visual inspection of the sequence in question using the Integrative Genomics Viewer (IGV) (Robinson *et al*, 2011).

### Sanger sequencing

Both DNA strands were sequenced using ABI 3730XL sequencing reagents (Applied Biosystems) and characterized by capillary electrophoresis on a 3730 DNA Analyzer (Applied Biosystems) at the OE Biotech (China). The PCR primers for Sanger sequencing analysis were as follows: *SLC10A1* (c.C800T) variant (F1: 5′‐GAGTGCAGTTGA TGGAAGT‐3′; R1: 5′‐CTGAGTGTATGTGGGGTTT‐3′) and *SEMA7A* (c.C442T) variant (F1: 5′‐CACTGCCCTACCTTCAAAC‐3′; R1: 5′‐TCTCCTCCCTTCTCTCTTC‐3′).

### Modeling of the R148W mutant

The X‐ray crystal structure of wild‐type SEMA7A was downloaded from PDB database (chain A in PDB entry 3NVQ). The in silico mutagenesis was carried out using the mutagenesis tool imbedded in PyMoL (v2.0; Schrödinger). The side chain rotamers of Trp replacing R148 were manually chosen to minimize spatial clashes. Surface electrostatic potential maps were calculated using the APBS (Adaptive Poisson‐Boltzmann Solver) plug‐in of PyMoL (V2.0; Schrödinger), and all figures were prepared using the PyMoL (V2.0; Schrödinger) molecular visualization program.

### Generation, characterization, and sample collection of Sema7a^R145W^ mutant mice

The C57BL/6J mice with c.433C>T mutation (p.R145W) in *Sema7a* were designed and generated by Shanghai Model Organisms Center (Shanghai, China), using the Cas9‐targeted guide RNA (sgRNA) of 5′‐ATGCCCGGAAGCCCAGCTGCTGG‐3′. The transcribed Cas9 mRNA and sgRNA as well as a 120‐base single‐stranded oligodeoxynucleotide (ssODN) were co‐injected into zygotes of C57BL/6J mice (Fig EV2A). The obtained F0 mice were characterized by PCR and sequencing using primer pairs: F1: 5′‐GGAGGGAACATGAGTTTGCT‐3′; R1: 5′‐CCACATGA CCACCGGCTACT‐3′. The F0 mice with the expected point mutation were bred to produce F1 mice (Fig EV2B). The genotype of F1 mice was characterized by PCR and confirmed by sequencing. The sequence of the ssODN for generation of point mutation mice was as follows: 5′‐AATTACATCACTCTTCTAGAAAGGCGGGGTAATGGGCTGCTGGTC TGTGGCACAAATGCCTGGAAGCCCAGCTGCTGGAACTTGGTAAGAACCCTTCCCATGTGCCTGAGTAGTCCCCAT‐3′ (Fig EV2C). *Sema7a* ^R145W^ homozygous and heterozygous mice were born without gross abnormalities and behavioral abnormalities. Blood samples from the tail vein of 4‐week‐old WT (*n* = 4, two male/two female), *Sema7a* ^R145W^ heterozygous (*n* = 5, three male/two female), and homozygous mice (*n* = 5, two male /three female) were collected to prepare individual serum samples for testing their liver function. Serum and liver samples from 8‐week‐old WT (*n* = 6, two male/four female), *Sema7a* ^R145W^ heterozygous (*n* = 10, six male/four female), and homozygous mice (*n* = 12, seven male/five female) were prepared. There was no significant difference in body weights, liver weights, and liver index among homozygous, heterozygous, and WT mice.

### Generation, characterization, and treatment of Slc10a1^S267F^ mutant mice

The mice with c.800C>T mutation (p.S267F) in murine *Slc10a1* were designed and generated by Shanghai Model Organisms Center (C57BL/6J mice, Shanghai, China). In brief, Cas9 mRNA was *in vitro* transcribed with mMESSAGE mMACHINE T7 Ultra Kit (Ambion, TX, USA), according to the manufacturer's instructions, and subsequently purified using the MEGAclearTM Kit (Thermo Fisher, USA). The Cas9 targeted guide RNA (sgRNA) of 5′‐CAGGGGGGAAGGTGACATTGAGG‐3′ was designed and transcribed *in vitro* using the MEGAshortscript Kit (Thermo Fisher), followed by purification using MEGAclearTM Kit. The obtained F0 mice were identified by PCR and sequencing using primers F1: 5′‐TTCAATCTCCCACTGTCTGCTCAA‐3′; R1: 5′‐GGCCTGTTTCAAGTTTTCCCTATG‐3′. The genotype of F1 mice was characterized by PCR and confirmed by sequencing. The sequence of the ssODN for generation of point mutation mice was 5′‐CTGCAGACGCACCATCAGCATGGAAACAGGATTCC AAAACGTCCAACTCTGTTTTACCATCCTGAATGTCACCTTCCCCCCTGAAGTCATTGGACCACTGTTCTTCTTTCCTCTCCTTTA‐3′. Serum and liver samples from 8‐week‐old WT (*n* = 4, two male/two female), *Slc10a1* ^S267F^ heterozygous (*n* = 4, two male/two female), and homozygous mice (*n* = 7, three male/four female) were prepared. Subsequently, the animals were fasted overnight and euthanized. Their blood samples were collected for preparing serum samples that were immediately stored at −80°C. Serum biochemistry was assayed in the Clinical Laboratory of the Southwest Hospital (Chongqing, China). Their liver tissues were quickly perfused with phosphate‐buffered saline (PBS) and immediately cut into small pieces, followed by frozen in liquid nitrogen.

All animal experiments were performed according to the guidelines of the Animal Care and Use Committees at the Medical Research Center. The study protocol was reviewed and approved by the Institutional Animal Care and Use Committee of the Southwest Hospital, Chongqing, China.

### Liquid chromatography/tandem mass spectrometry (LC‐MS/MS) analysis of bile acids in mouse liver tissue extracts

Mouse liver samples were prepared from WT, *Sema7a* ^R145W^ heterozygous, and homozygous mice and subjected to LC‐MS/MS analysis of bile acids (BA), as described previously (Liu *et al*, 2017; Lan *et al*, 2020). For analysis of BAs, 60 mg of each mouse liver sample (*n* = 5 per group) was homogenized in 100 μl Milli‐Q water and extracted with 500 μl of methanol. The solution was centrifuged at 14,000 *g* for 15 min at 4°C. The supernatant was dried in a freezer and re‐dissolved with 500 μl of acetonitrile/water (6:4, v/v). BA in the extracted mixture was separated using an ACQUITY BEH C18 column (1.7 µm, 100 mm × 2.1 mm internal dimensions; Waters, Milford, MA). Subsequently, the contents of BA were determined by LC‐MS/MS using an ACQUITY ultra‐performance liquid chromatography coupled with a XEVO TQ‐S mass spectrometer with an ESI source (Waters). The gradient elution procedure was as follows: 60–65% Buffer B during 0–6 min, 65–80% Buffer B during 6–13 min, 80–90% Buffer B during 13–13.5 min, and 90% Buffer B during 13.5–15 min (Buffer A = 0.1% formic acid; Buffer B = methanol). A mixture of standard BA was analyzed at intervals. The concentrations of each type of BA were calculated from the corresponding standard curves using the UPLC‐MS raw data. The LC‐MS/MS analysis of BAs was technically supported by Shanghai Applied Protein Technology.

### Sandwich culture of primary mouse hepatocytes

The isolation and sandwich culture of primary mouse hepatocytes were performed as described previously (Boyer *et al*, 1990; Wang *et al*, 2006). Briefly, primary hepatocytes were isolated from *Sema7a* ^R145W^ homozygous mice and WT mice. Next, these cells were cultured on 22‐mm round glass coverslips that had been coated with collagen type I (Corning, NY, USA, #354089) in William's E medium supplemented with 5% FBS, 1 μM dexamethasone, and 4 μg/ml insulin at 37°C in 5% CO_2_ for 9 h (1.5 ml per well of six‐well plate). The cultured cells were overlaid with 0.5% rat tail collagen I (Corning, NY, USA, #354236), according to the manufacturer's coating procedures. Two days later, the sandwich cultured primary hepatocytes were fixed in iced acetone and stored at −80°C until for further use.

### Transfection of primary mouse hepatocytes and human hepatoma HepG2 cells

The plasmids of pcDNA3.1‐*SEMA7A*_WT and *SEMA7A*_R148W for the expression of WT and non‐fusion mutant SEMA7A proteins were generated by Chongqing Yueqin Biotechnology (China). The cultured primary mouse hepatocytes before overlaying with collagen type I and human HepG2 cells were transfected with these constructs using transfection reagents (Effectene Transfection Reagents, Qiagen, Hilden, Germany, #301425 or FuGENE HD Transfection Reagents, Promega, Madison, WI, USA, #E2311, respectively), according to the manufacturer's protocol.

### RNA extraction, reverse transcription, and quantitative real‐time polymerase chain reaction (RT–qPCR)

Total RNA was extracted from mouse liver tissues using TRIzol reagent (Invitrogen, Carlsbad, CA, USA) according to the manufacturer's instructions. The RNA samples were reversely transcribed into cDNA as described previously (Liu *et al*, 2017) using the TaqMan probes (Life Technologies, Carlsbad, CA, USA). The PCRs were performed in duplicate using the SYBR Green Kit and specific primers (Appendix Table S17). The data were analyzed by $2^{‐\Delta \Delta \text{Ct}}$.

### Western blot analysis

Total liver tissue homogenates and whole cell lysates were prepared, as previously described (Chai *et al*, 2015; Cai *et al*, 2017, 2021; Liu *et al*, 2017). The lysate samples were separated by SDS–PAGE and transferred onto PVDF membranes (0.22 μm). After being blocked, the membranes were incubated with primary antibodies (Appendix Table S18). The bound antibodies were detected with HRP‐conjugated secondary antibodies and visualized with ECL. The data were analyzed by densitometric scanning using ImageJ software.

### Immunohistochemistry analysis

Immunohistochemistry (IHC) was performed as previously described (Chai *et al*, 2015; Liu *et al*, 2017) using the primary antibodies (Appendix Table S18).

### Multiplex immunofluorescence staining

Multiplex IF staining of liver sections and cells on the coverslips was performed as described previously (Chang *et al*, 2020) using anti‐Bsep and anti‐Mrp2 (Appendix Table S18) and multiplex immunohistochemistry/immunofluorescence staining kits (Absin, Shanghai, China, Cat# abs50030), according to the manufacturer's instructions.

### Liver histology

Liver sections were routine‐stained with H&E, and Sirius Red, as previously described (Chai *et al*, 2015; Liu *et al*, 2017).

### Statistical analysis

As indicated in the figure legends or table notes, all experimental data representing biological replicates (*n*) are expressed as mean ± SD and were analyzed by the independent‐samples Student's *t*‐test or the Mann–Whitney *U*‐test when applicable, using SPSS software (PASW Statistics 18, IBM; SPSS, Chicago, IL, USA). A two‐tailed *P*‐value of < 0.05 was considered statistically significant.

## Author contributions

JC conceived the studies; JC and S‐YC designed the experiments; QP, GL, JQ, XiZ, NZ, JD, ML, LL, YC, and XL performed the experiments; HY and LZ performed the bioinformatics analysis of WGS; WL performed structure modeling of the *SEMA7A* R148W mutant; JC, QP, SC, XiZ, JD, and QX analyzed the data; SC, XuZ, HZ, SF, QL, and GD contributed to specific reagents/materials/analysis tools; JC, S‐YC, and JLB wrote the manuscript.

## Conflict of interest

The authors declare that they have no conflict of interest.

## For more information

Research Group Website: www.cldcsw.org.

## Supporting information



Appendix

Expanded View Figures PDF

Source Data for Expanded View

Source Data for Figure 1

Source Data for Figure 2

Source Data for Figure 3

## Data Availability

This study generated data deposited in the following databases: [WGS data]: [BioProject] [accession number: PRJNA487655] (https://www.ncbi.nlm.nih.gov/bioproject/?term=PRJNA487655).
