# Supplementary material for: A homozygous R148W mutation in Semaphorin 7A causes progressive familial intrahepatic cholestasis
Source: EMBO Mol Med. 2021 Sep 29;13(11):e14563. doi: 10.15252/emmm.202114563 (PMC8573601; doi:10.15252/emmm.202114563)
Supplement: Supplementary file 1 — Appendix [file EMMM-13-e14563-s004.pdf]

Table of contents

Content	Page
**Appendix Figures. S1-3 and Appendix Tables. S1-18**	
**Appendix Figure S1.** Abdominal MRI (Patient IV.4) reveals normal liver size and parenchyma without any biliary tract obstruction and abnormalities.	3
**Appendix Figure S2.** Identification of c.C800T mutation in *SLC10A1* in the studied family.	4
**Appendix Figure S3.** Genomic organization of *SEMA7A* and mutation location.	5
**Appendix Table S1.** Clinical features of the child patient and her nuclear families	6
**Appendix Table S2.** Serum biochemistry of *Slc10a1*^{S267F}mutant mice (8-week-old)	7
**Appendix Table S3.** Clinical features of the child patient's family members.	8
**Appendix Table S4.** The autoantibody profile in family members.	11
**Appendix Table S5.** Blood viral hepatitis tests in family members.	12
**Appendix Table S6.** Biomarkers of liver fibrosis in family members.	13
**Appendix Table S7.** α1-antitrypsin and CER tests in the child patient.	13
**Appendix Table S8.** Blood lipid tests in family members.	13
**Appendix Table S9.** Variant filtering based on recessive inheritance model.	14
**Appendix Table S10.** Variant analysis based on recessive inheritance model.	15
**Appendix Table S11.** Variant filtering based on compound heterozygous inheritance model.	16
**Appendix Table S12.** Variant analysis based on compound heterozygous inheritance model.	17
**Appendix Table S13.** Variant filtering based on De novo model	18

**Appendix Table S14.** Variant filtering based on dominant inheritance model.	19
**Appendix Table S15.** Serum biochemistry of *Sema7a*^{R145W} mutant mice (4-week-old).	20
**Appendix Table S16.** LC-MS/MS analysis of bile acids in *Sema7a*^{R145W} mutant mouse livers	21
**Appendix Table S17.** Real time qPCR probes (TaqMan) and primers (SYBR).	22
**Appendix Table S18.** Antibodies used in western blot, immunohistochemistry and multiplex immunofluorescence.	23

Appendix Figures.S1-3 and their Figure legends

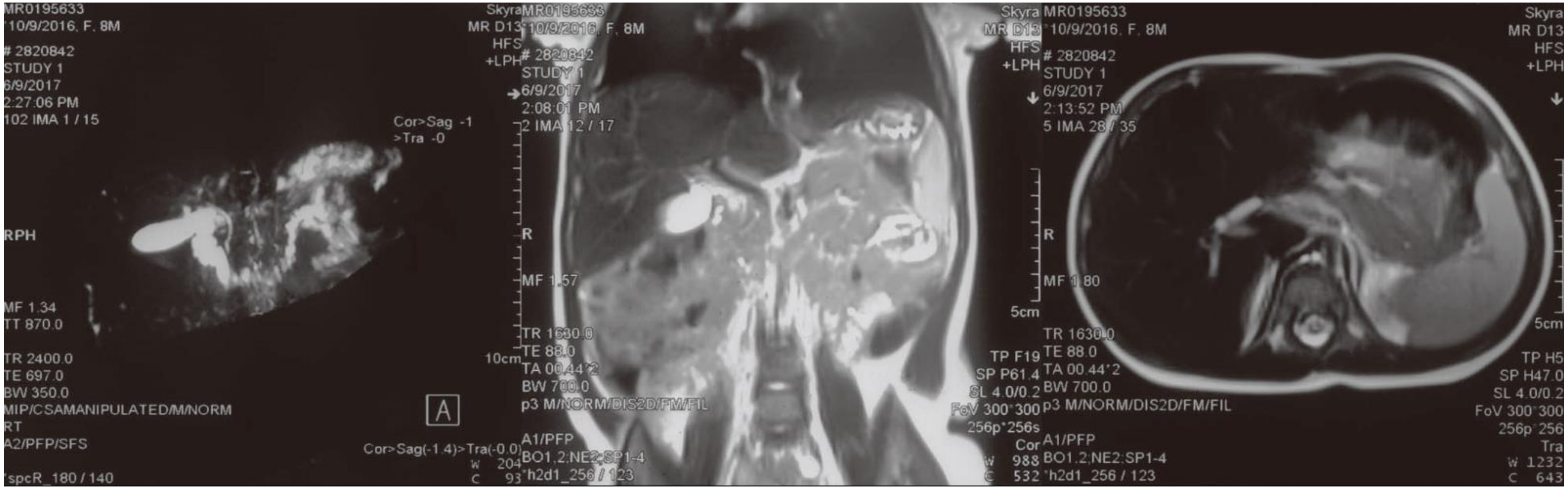

Appendix Figure S1. Abdominal MRI (Patient IV.4) reveals normal liver size and parenchyma without any biliary tract obstruction and abnormalities.

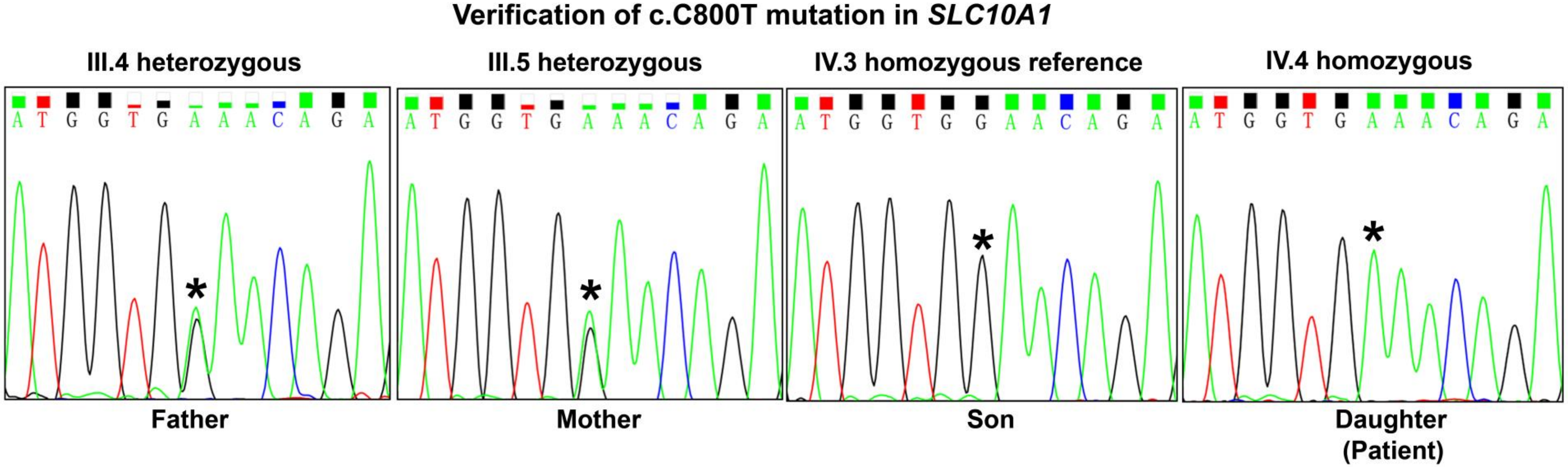

Appendix Figure S2. Identification of c.C800T mutation in *SLC10A1* in the studied family. Sanger DNA sequencing demonstrated that *SLC10A1* mutation was homozygous in the child patient (Patient IV.4). Her nuclear family members were homozygous reference or heterozygous for the mutation. The variable nucleotide(s) is specified (*).

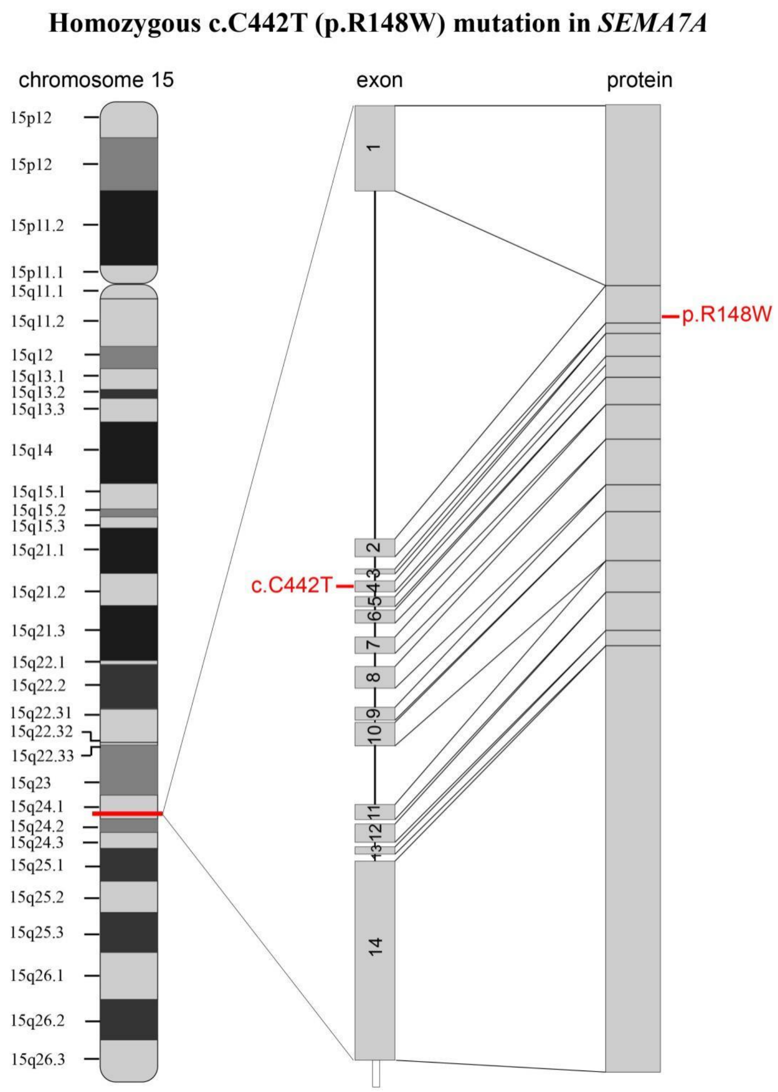

Appendix Figure S3. Genomic organization of *SEMA7A* and mutation location. Genomic organization of *SEMA7A* and the DNA and protein variants were identified in the patient IV.4 from the studied family.

Appendix Tables. S1-18

Appendix Table S1. Clinical features of the child patient and her nuclear families

**Family members parameters**		**Patient (IV.4)**		**Father (III.4)**	**Mother (III.5)**	**Brother (IV.3)**
**Sex, age**		**F, 2m**	**5m**	**M, 32y**	**F, 29y**	**M, 3y**
**Neurocognitive impairment**		-	-	-	-	-
**Hypotonia**		-	-		-	-
**Growth retardation**		-	-		-	-
**Serum biochemistry**	**Ref.**	**Patient-Test1**	**Patient-Test2**	**Father**	**Mother**	**Brother**
**ALT** (IU/L)	0-40	40.0	61.8	26.5	17.4	13.1
**AST** (IU/L)	0-37	76.0	146.9	23.9	20.3	24.3
**GGT** (IU/L)	4-50	N.D.	20.0	27.0	10.0	14.0
**ALP** (IU/L)	40-160 (Child<350)	N.D.	260.0	145.0	94.0	N.D.
**ALB** (g/L)	38-51	41.4	N.D.	N.D.	N.D.	N.D.
**TBIL** (μmol/l)	6-21	10.2	4.7	11.0	6.9	5.1
**DBIL** (μmol/l)	0-6	2.6	1.8	2.4	3.1	2.4
**TBA** (μmol/l)	0-10	154.1	101.4	4.3	2.9	1.9

Abbreviations: F, Female; M, Male; ALT, alanine aminotransferase; AST, aspartate aminotransferase; GGT, gamma-glutamyl transferase; ALP, alkaline phosphatase; ALB, albumin; TBIL, total bilirubin; DBIL, direct bilirubin; TBA, total bile acids.

Notes: N.D., not detected; "-" denotes negative.

Appendix Table S2. Serum biochemistry of *Slc10a1*^{S267F}mutant mice (8-week-old)

	**Wild type (n = 4)**	**Heterozygote (n = 4)**	**Homozygote (n = 7)**
**Gender (Male / Female)**	2/2	2/2	3/4
**Serum ALT (IU/L)**	22.98±8.71	37.18±19.65	29.95±7.89
**Serum AST (IU/L)**	76.54±13.33	81.60±20.74	75.15±22.04
**Serum ALP (IU/L)**	47.36±14.71	34.56±6.09	30.35±8.67
**Serum TBA (μmol/L)**	2.65±3.64	2.33±1.52	3.06±1.78
**Serum TBIL (μmol/L)**	5.00±0.80	7.30±4.14	6.55±2.44
**Serum DBIL (μmol/L)**	2.66±0.94	5.66±3.37	4.75±2.61

Notes: Values are mean ± SD. The data were analyzed by the independent-samples Student's *t*-test.

Abbreviations: ALT, alanine aminotransferase; AST, aspartate aminotransferase; ALP, alkaline phosphatase; TBA, total bile acids; TBIL, total bilirubin; DBIL, direct bilirubin.

Appendix Table S3. Clinical features of the child patient's family members

Family members		Serum biochemistry indexes							
		ALT (IU/L)	AST (IU/L)	GGT (IU/L)	ALP (IU/L)	ALB (g/L)	TBIL (μmol/l)	DBIL (μmol/l)	TBA (μmol/l)
**Ref.**		**0-40**	**0-37**	**4-50**	**40-160**	**38-51**	**6-21**	**0-6**	**0-10**
**I-1**	**M, 89y**	10.9	37.8	N.D.	152.0	54.8	14.4	1.5	5.7
**II-1**	**M, 63y**	11.2	21.7	N.D.	81.0	42.7	12.2	2.8	11.0
**II-2**	**F, 63y**	16.0	22.0	N.D.	N.D.	70.9	8.4	1.9	2.4
**II-3**	**M, 55y**	34.9	40.0	N.D.	126.0	63.5	14.6	6.0	2.4
**II-4**	**F, 54y**	23.1	31.3	N.D.	179.0	60.7	13.4	0.6	2.9
**II-5**	**M, 53y**	28.8	31.4	N.D.	142.0	62.1	20.3	6.9	2.0
**II-6**	**F, 52y**	24.6	26.9	N.D.	77.0	48.1	17.0	2.9	6.5

**II-7***	**M, 51y**	79.8	63.2	N.D.	170.0	68.4	16.7	3.8	1.9
**II-8**	**F, 53y**	27.3	28.8	N.D.	74.0	53.4	11.5	2.5	3.2
**II-9**	**F, 49y**	16.7	27.6	N.D.	84.0	48.4	11.1	2.2	1.3
**II-10**	**M, 48y**	33.1	26.8	N.D.	133.0	53.2	13.4	1.4	1.5
**III-1**	**M, 34y**	7.4	25.8	N.D.	96.0	52.4	13.5	3.0	6.1
**III-2**	**F, 34y**	8.2	20.8	N.D.	100.0	44.5	11.2	3.5	14.3
**III-3**	**F, 32y**	25.3	30.3	N.D.	122.0	67.8	15.4	4.8	1.9
**III-6**	**M, 31y**	29.5	30.4	N.D.	53.5	10.4	1.8	8.6	7.9
**III-7**	**M, 33y**	23.4	28.4	N.D.	129.0	59.1	15.5	3.2	1.8
**III-8**	**M, 32y**	26.5	34.9	N.D.	103.0	61.4	10.8	2.6	4.6
**III-9#**	**M, 28y**	68.4	45.2	N.D.	115.0	63.6	14.1	3.2	3.4

**III-10**	**M, 24y**	N.D.	N.D.	N.D.	N.D.	N.D.	N.D.	N.D.	N.D.
**IV-1**	**M, 8y**	10.2	12.1	N.D.	N.D.	49.0	7.7	1.6	2.2
**IV-2**	**M, 4y**	22.1	24.9	N.D.	N.D.	39.9	8.6	4.3	3.8

***Abbreviations*:** F, Female; M, Male; ALT, alanine aminotransferase; AST, aspartate aminotransferase; GGT, gamma-glutamyl transferase; ALP, alkaline phosphatase; ALB, albumin; TBA, total bile acids; TBIL, total bilirubin; DBIL, direct bilirubin; N.D., not detected.

Notes: *HBV patient; $^{\#}$ NASH patient

Appendix Table S4. The autoantibody profile in family members

	Family members	
	Patient IV.4	IV.3 (Brother)
U1RNP	-	-
Anti-Sm	-	-
SSA	-	-
Ro-52	-	-
SSB	-	-
Scl-70	-	-
PM-Scl	-	-
Jo-1	-	-
CENP	-	-
PCNA	-	-
ds-DNA	-	-
Nucleosome	-	-
Histone	-	-
Ribosome P protein	-	-
AMA type M2	-	-

Abbreviations**:** U1RNP, U1 ribonucleoprotein; Anti-Sm, Anti-Smith; SSA, Sjogren's Syndrome A; Ro-52, 52 kDa Ro protein; SSB, Sjogren's Syndrome B; Scl-70, Scleroderma-70; PM-Scl, polymyositis-Scleroderma; CENP, Centromere Protein; PCNA, Proliferating Cell Nuclear Antigen; ds-DNA, double stranded deoxyribonucleic acid; AMA, anti-mitochondrial antibody.

Notes: “-” denotes negative.

Appendix Table S5. Blood viral hepatitis tests in family members

	Family members	
	**Patient IV.4**	**IV.3 (Brother)**
HAV IgM	-	-
HBsAg	-	-
HBsAb	+	+
HBeAb	-	-
HBeAg	-	-
HBcAb	-	-
HBc IgM	-	-
HCV Ab	-	-
EBV Antibody	-	-
CMV Antibody	-	-

Abbreviations**:** HAV lgM, hepatitis A lgM; HBsAg, hepatitis B surface antigen; HBsAb, hepatitis B surface antibody; HBeAb, hepatitis B e antibody; HBeAg, hepatitis B e antigen; HBcAb, hepatitis B core antibody; HBc IgM, anti-hepatitis B core IgM; HCV Ab, hepatitis C virus antibody; EBV, Epstein-Barr virus; CMV, cytomegalovirus;

Notes: "+" denotes positive; "-" denotes negative.

Appendix Table S6. Biomarkers of liver fibrosis in family members

Biomarkers	Family members		
	Ref.	Patient IV.4	IV.3 (Brother)
Hyaluronic acid (ng/ml)	0-100	50.0	50.0
Procollagen type III (ng/ml)	0.021-30	45.5	36.4
Type IV collagen (ng/ml)	0.021-30	39.3	18.0
Laminin (ng/ml)	0.021-30	20.0	20.0

Appendix Table S7. α1-antitrypsin and CER tests in the child patient

Biomarkers	The child patient	
	Ref.	Patient IV.4
α1-antitrypsin (mg/dl)	88-174	108.0
Ceruloplasmin (CER) (mg/dl)	21-53	20.3

Appendix Table S8. Blood lipid tests in family members

Biomarkers	Family members		
	Ref.	Patient IV.4	IV.3 (Brother)
TG (mmol/L)	0.40-1.70	1.11	1.14
Tch (mmol/L)	3.00-5.20	3.85	3.45
LDL-c (mmol/L)	1.62-3.36	2.03	1.94
HDL-c (mmol/L)	0.91-1.55	1.33	1.11

Abbreviations**:** TG, triglycerides; Tch, total cholesterol; LDL-c, Cholesterol LDL; HDL-c, High-density lipoprotein.

Appendix Table S9. Variant filtering based on recessive inheritance model

Steps for filtering variants	The nuclear family (III.4, III.5, IV.3, and Patient IV.4)
Total number of SNPs/Indels after quality control and coverage.	4.88 million
Number of SNPs/Indels for model.	155 053
Number of non-synonymous or frameshift (deletion/insertion) SNPs/Indels in exons or splicing sites.	384
Rare (1) non-synonymous or frameshift (deletion/insertion) SNPs/Indels in exons or splicing sites.	1 (*SEMA7A*)
Rare (1) non-synonymous or frameshift (deletion/insertion) SNPs/Indels in exons or splicing sites were predicted as "damaging" with PolyPhen-2 (Adzhubei *et al*, 2010) or "disease-causing" with MutationTaster (Schwarz *et al*, 2014).	1 (*SEMA7A*)

Notes: (1) The minor allele frequency (MAF) of rare variant less than 0.01 (1%) (Merico *et al*, 2015). MAF sourced from the Asian cohort in Exome Aggregation Consortium (ExAC) database (Lek *et al*, 2016) and/or 1000 Genomes database (Abecasis *et al*, 2010). Human genome assembly used in this analysis was Genome Reference Consortium GRCh37(hg19).

Appendix Table S10. Variant analysis based on recessive inheritance model

Gene	Variant (type)	Mouse Model (phenotype)[a]	Human disease (OMIM)[b]	Clinical significance (ClinVar database)[c]	Reference
SEMA7A NM_003612	c.C442T missense	no	no	no	SEMA7A servers as an effect or molecule in T-cell-mediated inflammation (Alto & Terman, 2017; Suzuki *et al*, 2007).

Notes: [a] Mouse model sourced from http://www.informatics.jax.org/

[b] OMIM, online Mendelian inheritance in man, https://www.ncbi.nlm.nih.gov/omim

[c] ClinVar database, https://www.ncbi.nlm.nih.gov/clinvar/

Human genome assembly used in this analysis was Genome Reference Consortium GRCh37(hg19).

Appendix Table S11. Variant filtering based on compound heterozygous inheritance model

Steps for filtering variants	The nuclear family (III.4, III.5, IV.3, and Patient IV.4)
Total number of SNPs/Indels after quality control and coverage.	4.88 million
Number of SNPs/Indels for model.	17 308
Number of non-synonymous or frameshift (deletion/insertion) SNPs/Indels in exons or splicing sites.	405
Rare (1) non-synonymous or frameshift (deletion/insertion) SNPs/Indels in exons or splicing sites.	33
Rare (1) non-synonymous or frameshift (deletion/insertion) SNPs/indels in exons or splicing sites, and at least one SNPs/Indels were predicted as "damaging" with PolyPhen-2 (Adzhubei *et al.*, 2010) or "disease-causing" with MutationTaster (Schwarz *et al.*, 2014).	2 (*CC2D2B*)

Notes: (1) The minor allele frequency (MAF) of rare variant less than 0.05 (5%) (Merico *et al.*, 2015). MAF sourced from the Asian cohort in Exome Aggregation Consortium (ExAC) database (Lek *et al.*, 2016) and/or 1000 Genomes database (Abecasis *et al.*, 2010) Human genome assembly used in this analysis was Genome Reference Consortium GRCh37(hg19).

Appendix Table S12. Variant analysis based on compound heterozygous inheritance model

Gene	Variant (type)	Mouse Model (phenotype)[a]	Human disease (OMIM)[b]	Clinical significance (ClinVar database)[c]	Reference
CC2D2B NM_001159747	c.A190G missense	no	no	no	The function of CC2D2B, especially in cancer, is virtually unknown; CC2D2B is a top upregulated gene in papillary thyroid carcinomas (Schulten *et al*, 2016).
	c.T818A missense			no	

Notes: [a] Mouse model sourced from http://www.informatics.jax.org/

[b] OMIM, online Mendelian inheritance in man, https://www.ncbi.nlm.nih.gov/omim

[c] ClinVar database, https://www.ncbi.nlm.nih.gov/clinvar/

Human genome assembly used in this analysis was Genome Reference Consortium GRCh37(hg19).

Appendix Table S13. Variant filtering based on De novo model

**Steps for filtering variants**	**The nuclear family (III.4, III.5, IV.3, and Patient IV.4)**
Total number of SNPs/indels after quality control and coverage.	4.88 million
Number of SNPs/Indels for model	17 129
Number of non-synonymous or frameshift (deletion/insertion) SNPs/Indels in exons or splicing sites.	29
Rare (1) non-synonymous or frameshift (deletion/insertion) SNPs/Indels in exons or splicing sites.	5
Rare (1) non-synonymous or frameshift (deletion/insertion) SNPs/Indels in exons or splicing sites were predicted as "damaging" with PolyPhen-2 (Adzhubei *et al.*, 2010) or "disease-causing" with MutationTaster (Schwarz *et al.*, 2014).	0

Notes: (1) The minor allele frequency (MAF) of rare variant less than 0.01 (1%) (Merico *et al.*, 2015). MAF sourced from the Asian cohort in Exome Aggregation Consortium (ExAC) database (Lek *et al.*, 2016) and/or 1000 Genomes database (Abecasis *et al.*, 2010). Human genome assembly used in this analysis was Genome Reference Consortium GRCh37(hg19).

Appendix Table S14. Variant filtering based on dominant inheritance model

Steps for filtering variants	The nuclear family (III.4, III.5, IV.3, and Patient IV.4)
Total number of SNPs/Indels after quality control and coverage.	-
Number of SNPs/Indels for model	-
Number of non-synonymous or frameshift (deletion/insertion) SNPs/Indels in exons or splicing sites.	-
Rare (1) non-synonymous or frameshift (deletion/insertion) SNPs/Indels in exons or splicing sites.	-
Rare (1) and non-synonymous or frameshift (deletion/insertion) SNPs/Indels in exons or splicing sites were predicted as "damaging" with PolyPhen-2 (Adzhubei *et al.*, 2010) or "disease-causing" with MutationTaster (Schwarz *et al.*, 2014).	-

Notes: (1) The minor allele frequency (MAF) of rare variant less than 0.01 (1%) (Merico *et al.*, 2015). MAF sourced from the Asian cohort in Exome Aggregation Consortium (ExAC) database (Lek *et al.*, 2016) and/or 1000 Genomes database (Abecasis *et al.*, 2010). Human genome assembly used in this analysis was Genome Reference Consortium GRCh37(hg19).

Appendix Table S15. Serum biochemistry of *Sema7A*^{R145W} mutant mice (4-week-old)

	Wild type (n=4)	Heterozygote (n=5)	Homozygote (n=5)
**Gender (Male / Female)**	2/2	3/2	2/3
**Serum ALT (IU/L)**	22.60±4.59	23.36±3.46	37.92±11.81*,#
**Serum AST (IU/L)**	97.20±29.26	124.80±28.76	170.72±65.18$^{*(0.067)}$
**Serum ALP (IU/L)**	278.00±36.00	267.20±41.03	304.00±43.08
**Serum TBA (μmol/L)**	3.70±2.62	3.58±1.75	9.54±4.67$^{*(0.062)}$, #
**Serum TBIL (μmol/L)**	8.40±3.05	6.69±4.95	8.91±2.83
**Serum DBIL (μmol/L)**	5.42±1.88	4.16±4.18	5.63±1.63

Notes: Values are mean ± SD. *P < 0.05 versus the WT mice, #P < 0.05 versus the heterozygote mutant mice. The data were analyzed by the independent-samples Student's *t-test*.

Abbreviations: ALT, alanine aminotransferase; AST, aspartate aminotransferase; ALP, alkaline phosphatase; TBA, total bile acids; TBIL, total bilirubin; DBIL, direct bilirubin.

Appendix Table S16. LC-MS/MS analysis of bile acids in *Sema7a*^{R145W} mutant mouse livers

	**Wild type (n = 5)**	**Heterozygote (n = 5)**	**Homozygote (n = 5)**
Taurocholic acid (TCA)	154870.65 ±95237.73	293970.65 ±68121.16*	512596.59 ±139355.52*,#
Tauromuricholic acid (TMCA)	4301.90 ±3837.45	32405.68 ±21228.12*	73559.29 ±40057.31*
Taurodeoxycholic acid (TDCA)	6406.14 ±3728.14	4141.65 ±1438.89	7975.04 ±2677.18#
Taurochenodeoxycholic acid (TCDCA)	3590.60 ±2389.83	150752.03 ±315159.52*	157225.77 ±135314.00*
Taurohyodeoxycholic acid (THDCA)	213.27 ±187.24	1911.45 ±1611.67*	6026.29 ±4735.07*
Taurolithocholic acid (TLCA)	145.66 ±142.34	85.99 ±44.23	239.56 ±129.62#

Notes: Values are mean ± SD (ng/g of liver). *P < 0.05 versus the WT mice, #P < 0.05 versus the heterozygous mutant mice. The data were analyzed by the independent-samples Student's *t*-test or the Mann-Whitney *U* test when applicable.

Appendix Table S17. The sequences of real time qPCR probes (TaqMan) and primers (SYBR)

Gene	Sequences (5’→3’)	Species/Source
Bsep (Abcb11)	Proprietary to ABI	Mouse/Mm00445168_m1
Mrp2 (Abcc2)	Proprietary to ABI	Mouse/Mm00496899_m1
Mdr1b (Abcb1b)	Proprietary to ABI	Mouse/Mm 00440736 _m1
Mdr2 (Abcb4)	Proprietary to ABI	Mouse/Mm00435630_m1
Abcg5	Proprietary to ABI	Mouse/Mm00446241_m1
Abcg8	Proprietary to ABI	Mouse/Mm00445980_m1
Mrp3 (Abcc3)	Proprietary to ABI	Mouse/Mm00551550_m1
Mrp4 (Abcc4)	Proprietary to ABI	Mouse/Mm01226381_m1
Ostβ (Slc51b)	Proprietary to ABI	Mouse/Mm01175040_m1
Ntcp (Slc10a1)	Proprietary to ABI	Mouse/Mm00441421_m1
Oatp1a1 (Slco1a1)	Proprietary to ABI	Mouse/Mm01267415_m1
Cyp7a1	Proprietary to ABI	Mouse/Mm00484150_m1
Cyp7b1	Proprietary to ABI	Mouse/Mm00484157_m1
Cyp8b1	Proprietary to ABI	Mouse/Mm00501637_s1
Cyp27a1	Proprietary to ABI	Mouse/Mm00470430_m1
Gapdh	Proprietary to ABI	Mouse/Mm99999915_g1
Gapdh	Forward: 5’-CTTTGTCAAGCTCATTTCCTGG-3’	Mouse/ Primers (SYBR)
	Reverse: 5’-TCTTGCTCAGTGTCCTTGC-3’	NM_008084.3
Cyp2c70	Forward: 5’-TTGACCAGGGAGATGAGTTTTC-3’	Mouse/ Primers (SYBR)
	Reverse: 5’-CCCCATAGACCTTAAGACCATG-3’	NM_145499.2

Appendix Table S18. Antibodies used in western blot, immunohistochemistry and multiplex immunofluorescence

Protein	Host	Company / Catalog	Antibody dilution
Gapdh	Rabbit	Proteintech, Chicago, IL/10494-1-AP	WB 1:3000
CK19	Rabbit	Abcam, Cambridge, MA/ab52625	IHC 1:50
Bsep	Mouse	Santa Cruz, Dallas, CA/sc-74500	WB 1:1000
Mrp2	Mouse	Abcam, Cambridge, MA/ab3373	WB 1:2000
Mdr2	Rabbit	Invitrogen, Carlsbad, CA/PA5-78692	WB 1:3000
Mrp3	Mouse	Santa Cruz, Dallas, CA/sc-5776	WB 1:1600
Mrp4	Rat	Abcam, Cambridge, MA/ab15602	WB 1:1600
Ostα	Rabbit	Santa Cruz, Dallas, CA/sc-100078	WB 1:2000
Ostβ	Goat	Santa Cruz, Dallas, CA/sc-163192	WB 1:500
Ntcp	Rabbit	Boster, Pleasanton,CA/BA1674	WB 1:1000
Cyp7a1	Rabbit	Santa Cruz, Dallas, CA/sc-25536	WB 1:1000
Cyp8b1	Goat	Santa Cruz, Dallas, CA/sc-23515	WB 1:2000
p-PKCδ(pS299)	Rabbit	Epitomics, Burlingme, CA/5488-S	WB 1:5000
PKCδ(C-20)	Rabbit	Santa Cruz, Dallas, CA/sc-937	WB 1:2000
p-PKCε(Ser729)	Rabbit	Bioss Antibodies, Woburn, MA/bs-6948	WB 1:1000
PKCε(C-15)	Rabbit	Santa Cruz, Dallas, CA/sc-214	WB 1:2000
Bsep	Rabbit	Proteintech, Chicago, IL/18990-1-AP	IF 1:100
Mrp2	Rabbit	LifeSpan, Seattle, WA/LS-B1428	IF 1:50
