# Supplementary material for: A homozygous R148W mutation in Semaphorin 7A causes progressive familial intrahepatic cholestasis
Source: EMBO Mol Med. 2021 Sep 29;13(11):e14563. doi: 10.15252/emmm.202114563 (PMC8573601; doi:10.15252/emmm.202114563)
Supplement: Supplementary file 2 — Expanded View Figures PDF [file EMMM-13-e14563-s005.pdf]

Expanded View Figures

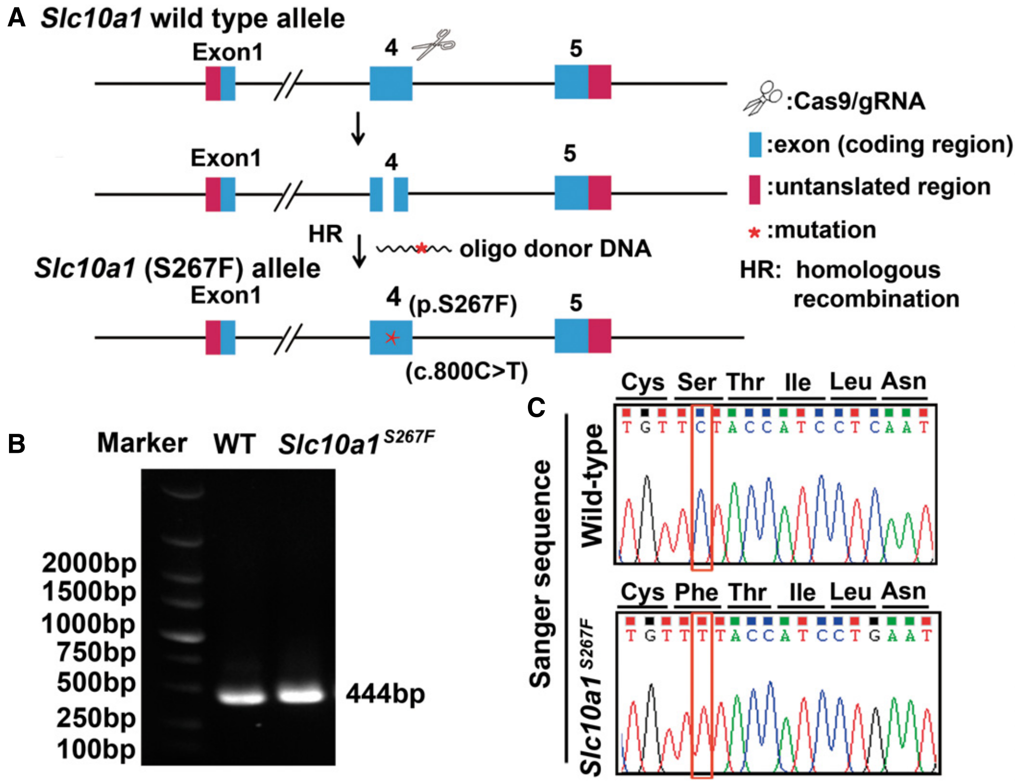

Figure EV1. Generation and characterization of *Slc10a1*^{S267F} mutant mice.

A Schematic diagram for the generation of *Slc10a1*^{S267F} mutant mice.

B Genotyping of *Slc10a1*^{S267F} mutant mice. Genomic DNA was extracted from mouse tails, and the specific region covering the mutation was amplified by PCR.

C Identification of *Slc10a1*^{S267F} homozygous mice by Sanger sequencing analysis.

Source data are available online for this figure.

 © 2021 The Authors

12 of 11 EMBO Molecular Medicine 13: e14563 | 2021 © 2021 The Authors

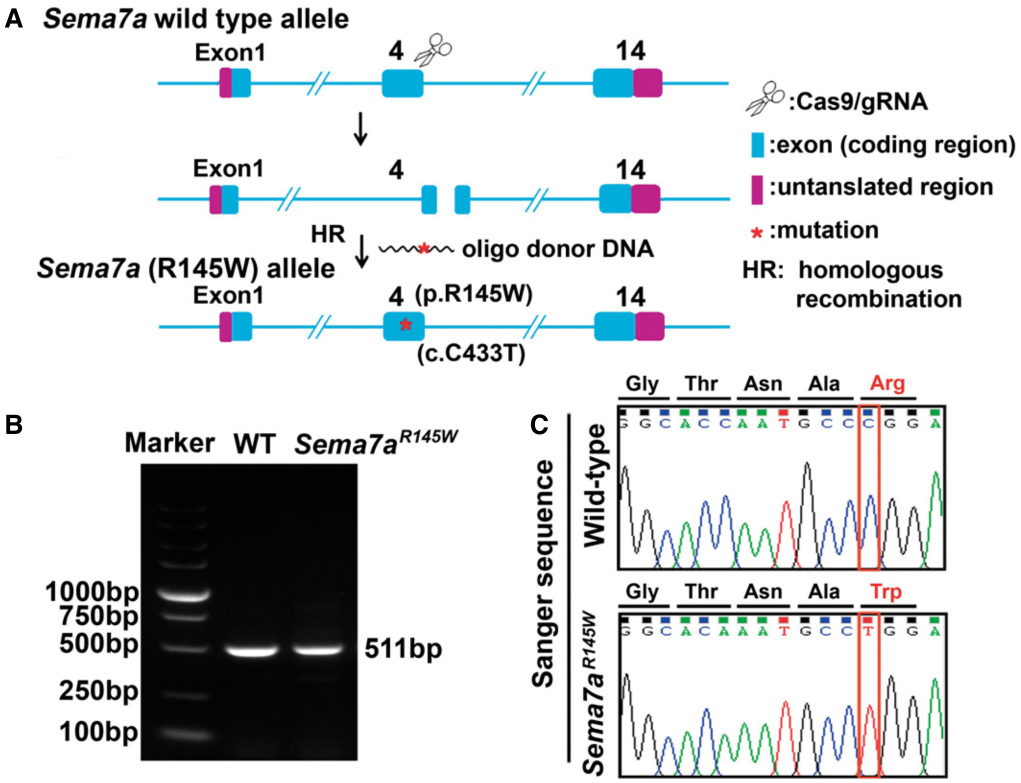

Figure EV2. Generation and characterization of *Sema7a*^{R145W} mutant mice.

A Schematic diagram for the generation of *Sema7a*^{R145W} mutant mice.
B Genotyping *Sema7a*^{R145W} mutant mice. Genomic DNA was extracted from mouse tails, and the specific region was amplified by PCR.
C Identification of *Sema7a*^{R145W} homozygous mice by Sanger sequencing analysis.

Source data are available online for this figure.

© 2021 The Authors EMBO Molecular Medicine 13: e14563 | 2021 13 of 11

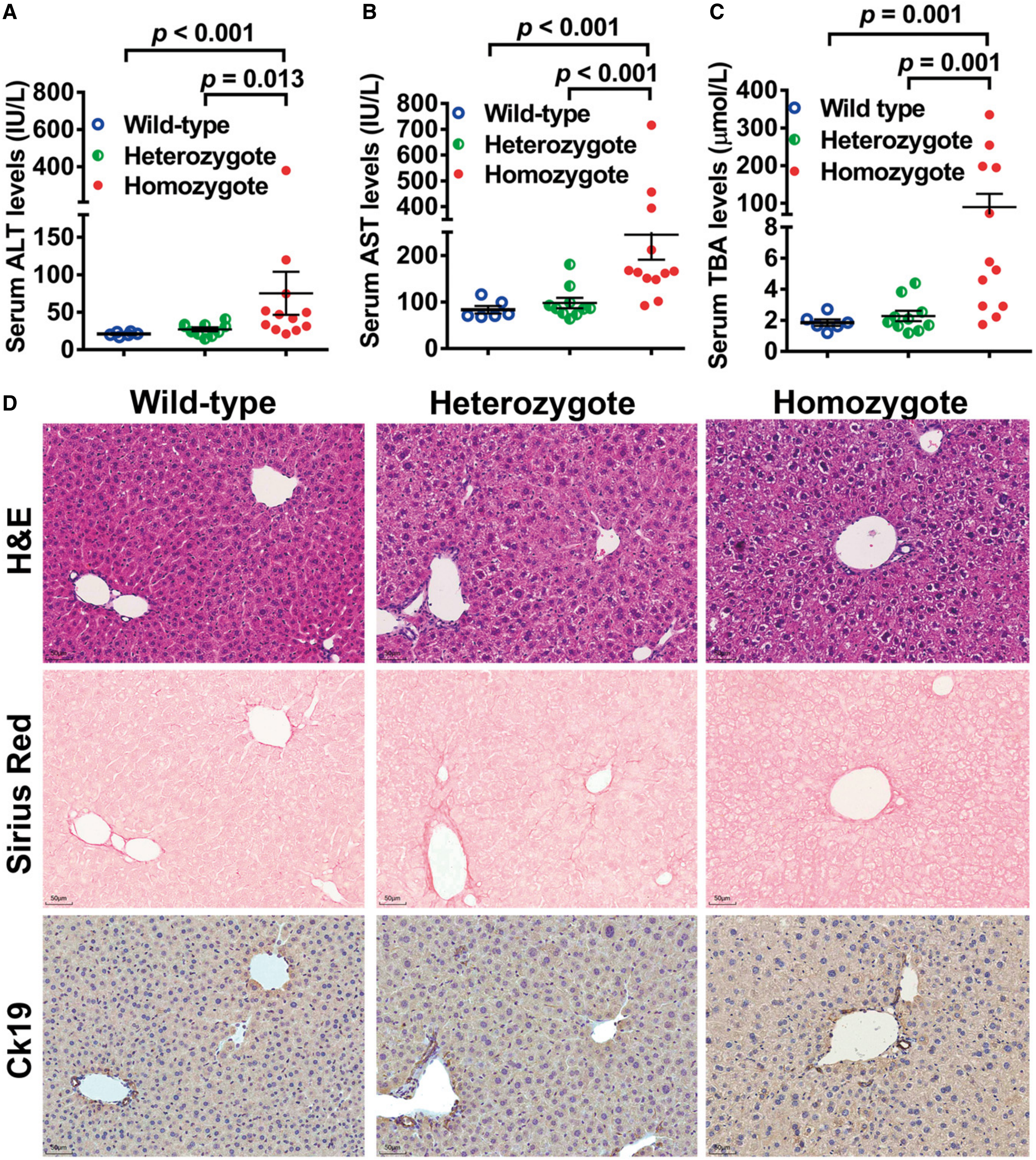

Figure EV3.

© 2021 The Authors

© 2021 The Authors

Figure EV3. A *Sema7a*^{R145W} homozygous mutation causes the elevated serum ALT, AST, and TBA levels and remarkable hydropic degeneration in mouse livers.

A–C Serum ALT, AST, and TBA in *Sema7a*^{R145W} WT (n = 6, two male/four female), heterozygous (n = 10, six male/four female) and homozygous mice (n = 12, seven male/five female). Data are shown as means ± SD.

D Representative images of H&E staining, *Sirius Red* staining, and IHC analysis of CK19 expression in WT, *Sema7a*^{R145W} heterozygous and homozygous mice. The *Sema7a*^{R145W} homozygous mutation caused significant liver injury with elevated levels of serum ALT, AST, and TBA and striking hydropic degeneration in hepatocytes. Scale bar, 50 μm.

Data information: The data were analyzed by the Mann–Whitney *U*-test.

Source data are available online for this figure.

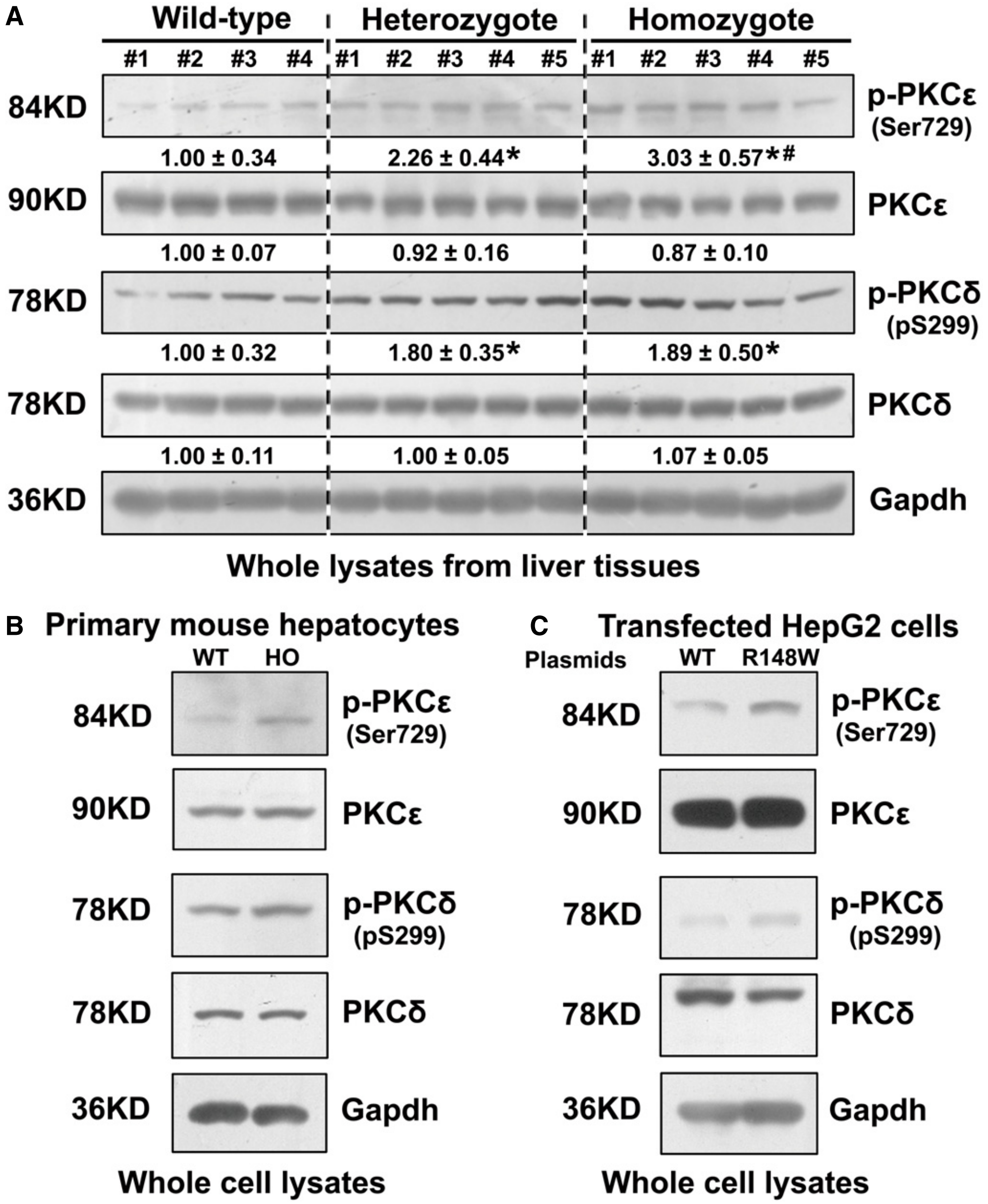

Figure EV4.

Figure EV4. The *Sema7a*^{R145W} mutation increases PKCδ/ε phosphorylation in hepatocytes.

A The levels of hepatic PKCδ/ε phosphorylation were significantly higher in *Sema7a*^{R145W} homozygous mice than in heterozygous mice and WT mice. Wild type, wild-type mice, $n = 4$; Heterozygote, *Sema7a*^{R145W} heterozygous mice, $n = 5$; homozygote, *Sema7a*^{R145W} homozygous mice, $n = 5$. $^*P < 0.05$ versus the WT mice, $^\#P < 0.05$ versus the heterozygous mutant mice. The data were analyzed by the independent-samples Student's *t*-test and the Mann–Whitney *U*-test.

B, C Furthermore, *Sema7a*^{R145W} (human R148W) homozygous mutation increased PKCδ/ε phosphorylation in *Sema7a*^{R145W} mouse primary hepatocytes (B) and human HepG2 cells following transfection with *SEMA7A*_R148W construct (C).

Source data are available online for this figure.

 © 2021 The Authors
