# Supplementary figures and images for: A homozygous R148W mutation in Semaphorin 7A causes progressive familial intrahepatic cholestasis
Source: EMBO Mol Med. 2021 Sep 29;13(11):e14563. doi: 10.15252/emmm.202114563 (PMC8573601; doi:10.15252/emmm.202114563)

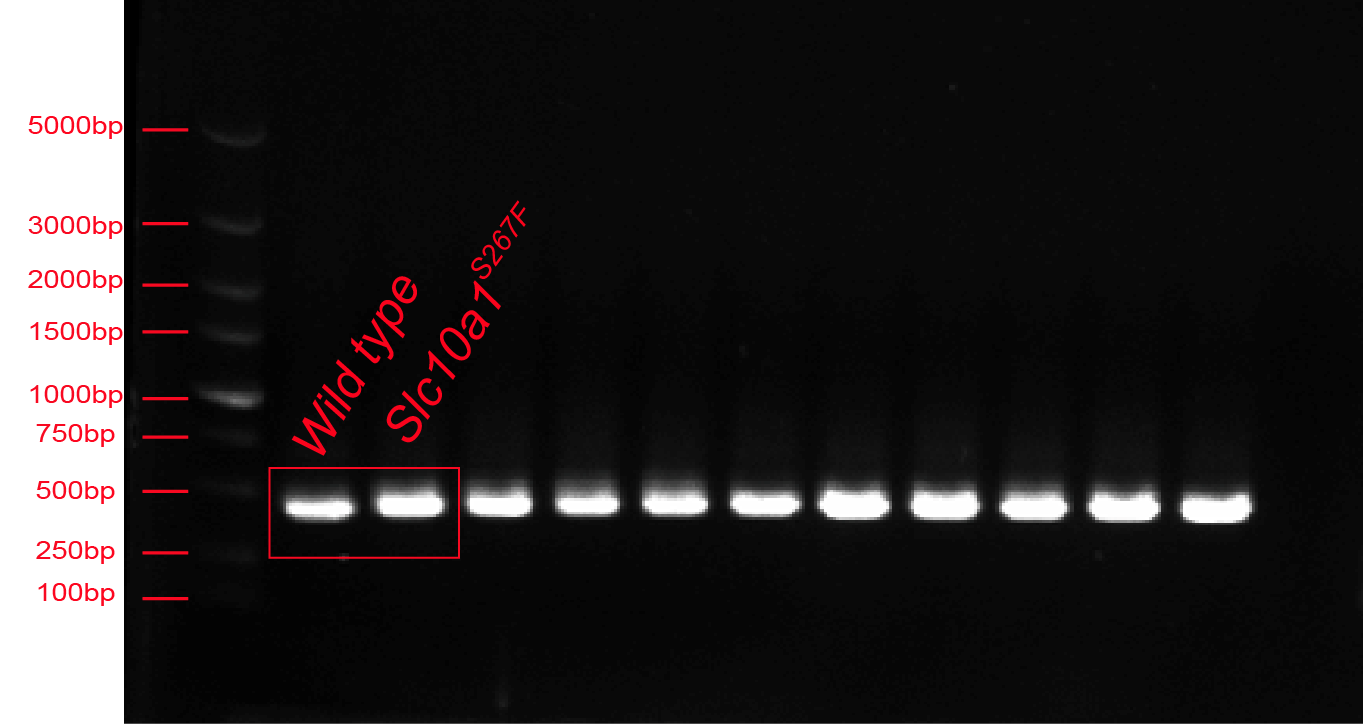

Supplement: Supplementary file 3 — Source Data for Expanded View [file EMMM-13-e14563-s002.zip › Source Data EV Figure 1B.tif]

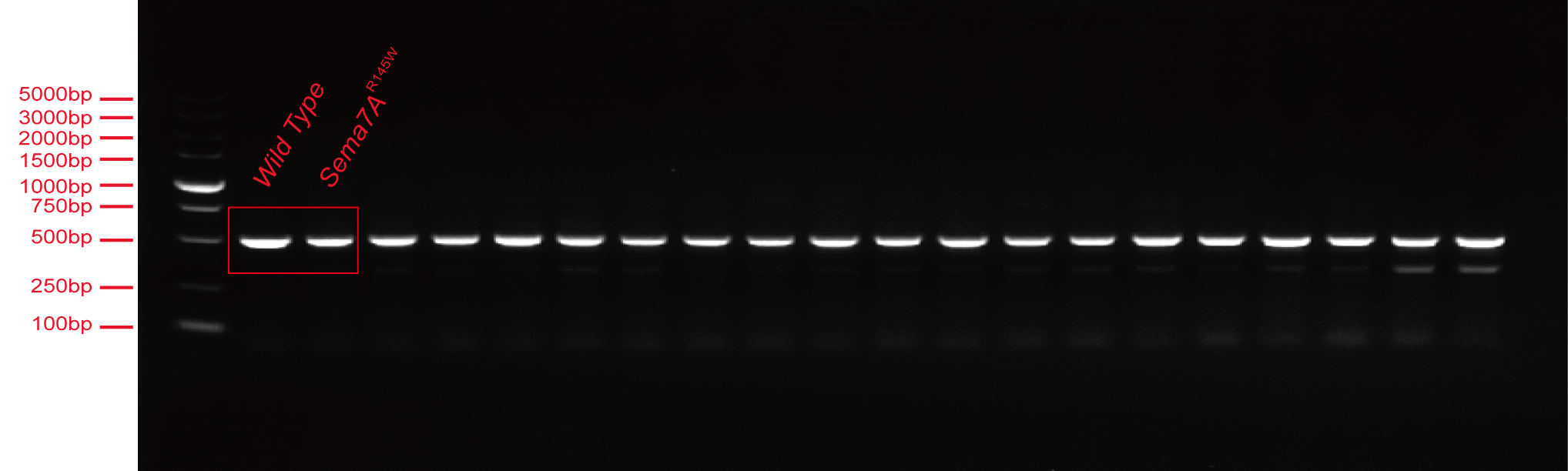

Supplement: Supplementary file 3 — Source Data for Expanded View [file EMMM-13-e14563-s002.zip › Source Data EV Figure 2B.tif]

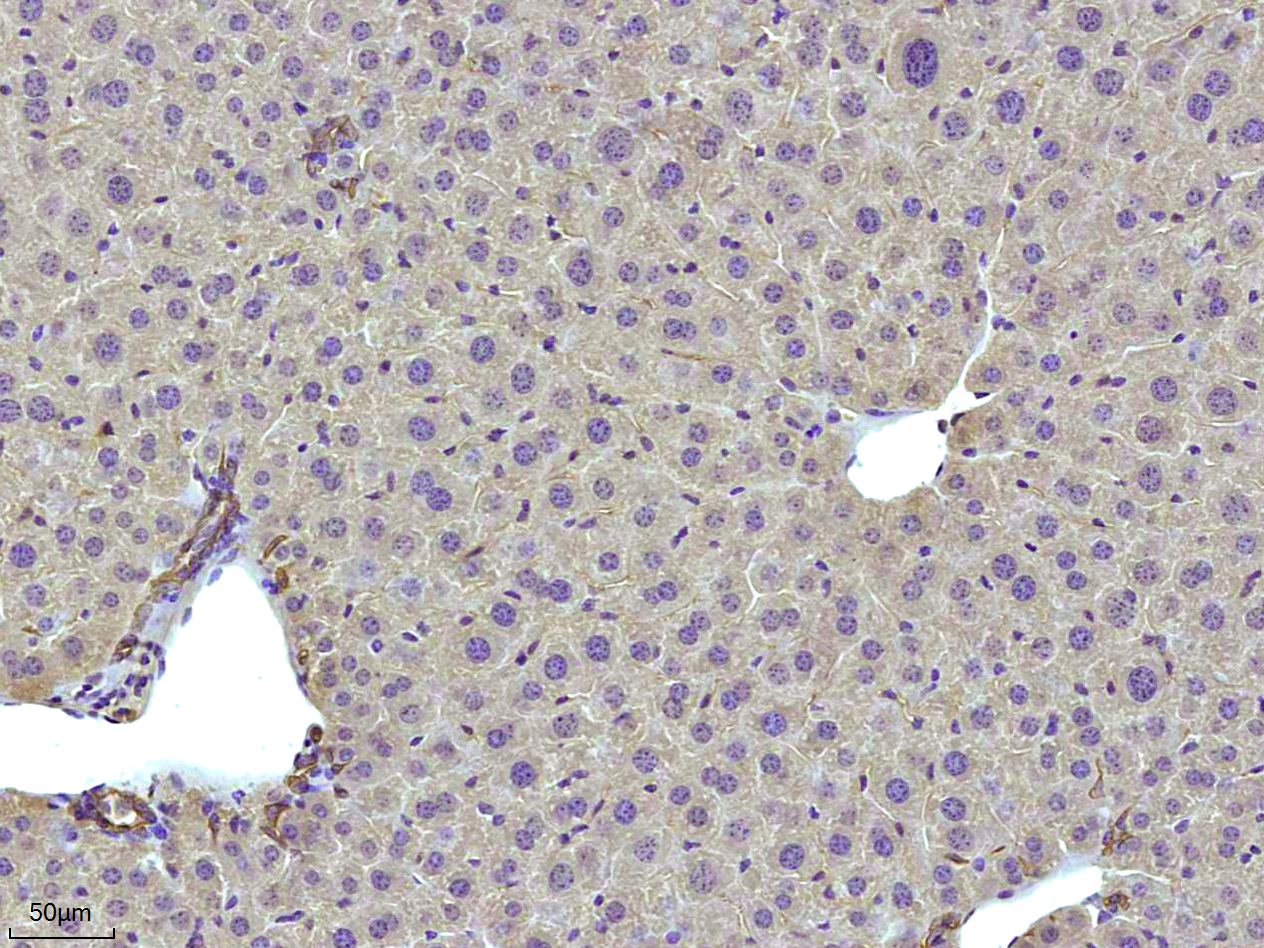

Supplement: Supplementary file 3 — Source Data for Expanded View [file EMMM-13-e14563-s002.zip › Source Data EV Figure 3/Source Data EV Figure 3D/Ck19/Heterozygote_Ck19.tif]

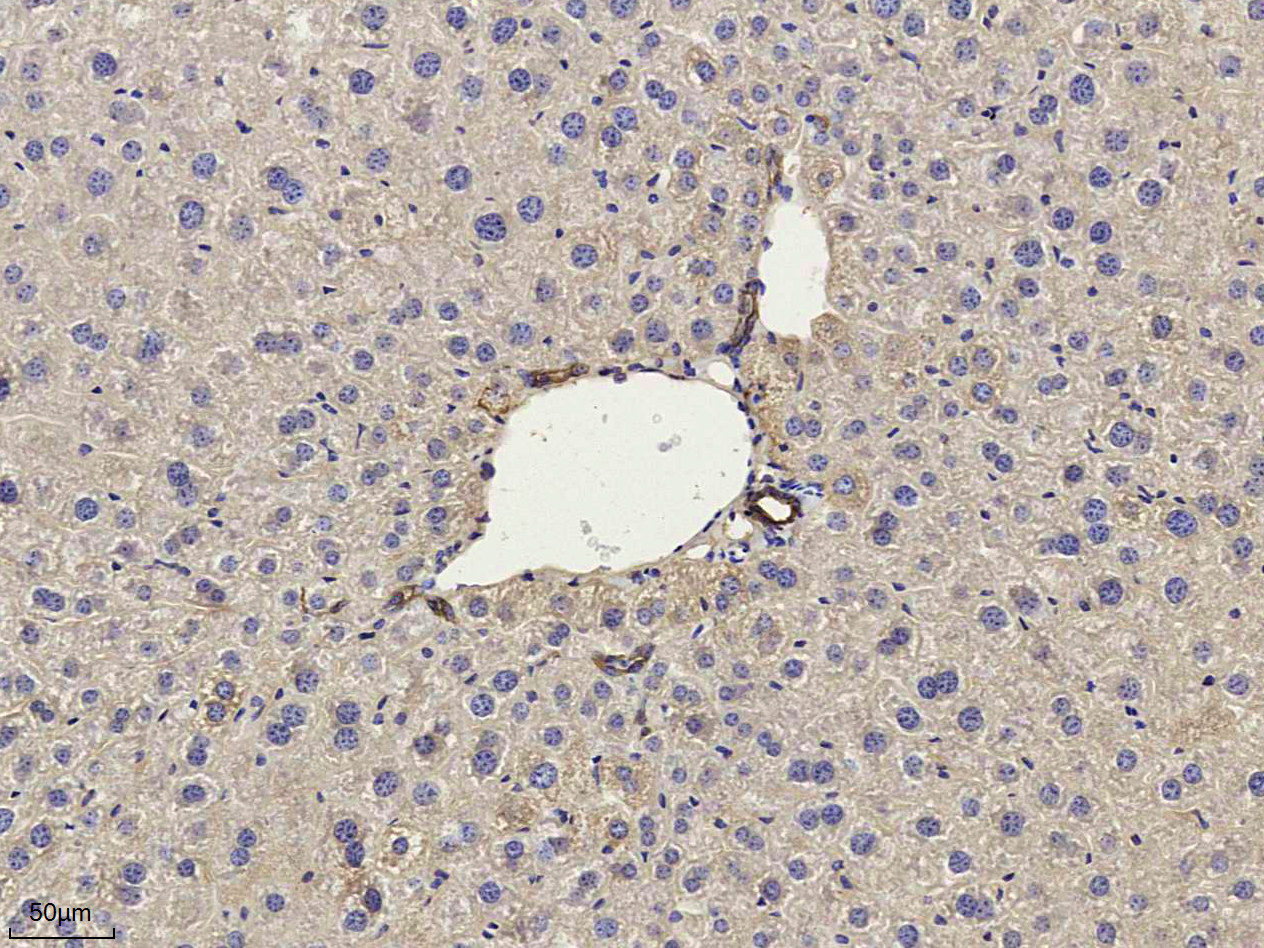

Supplement: Supplementary file 3 — Source Data for Expanded View [file EMMM-13-e14563-s002.zip › Source Data EV Figure 3/Source Data EV Figure 3D/Ck19/Homozygote_Ck19.tif]

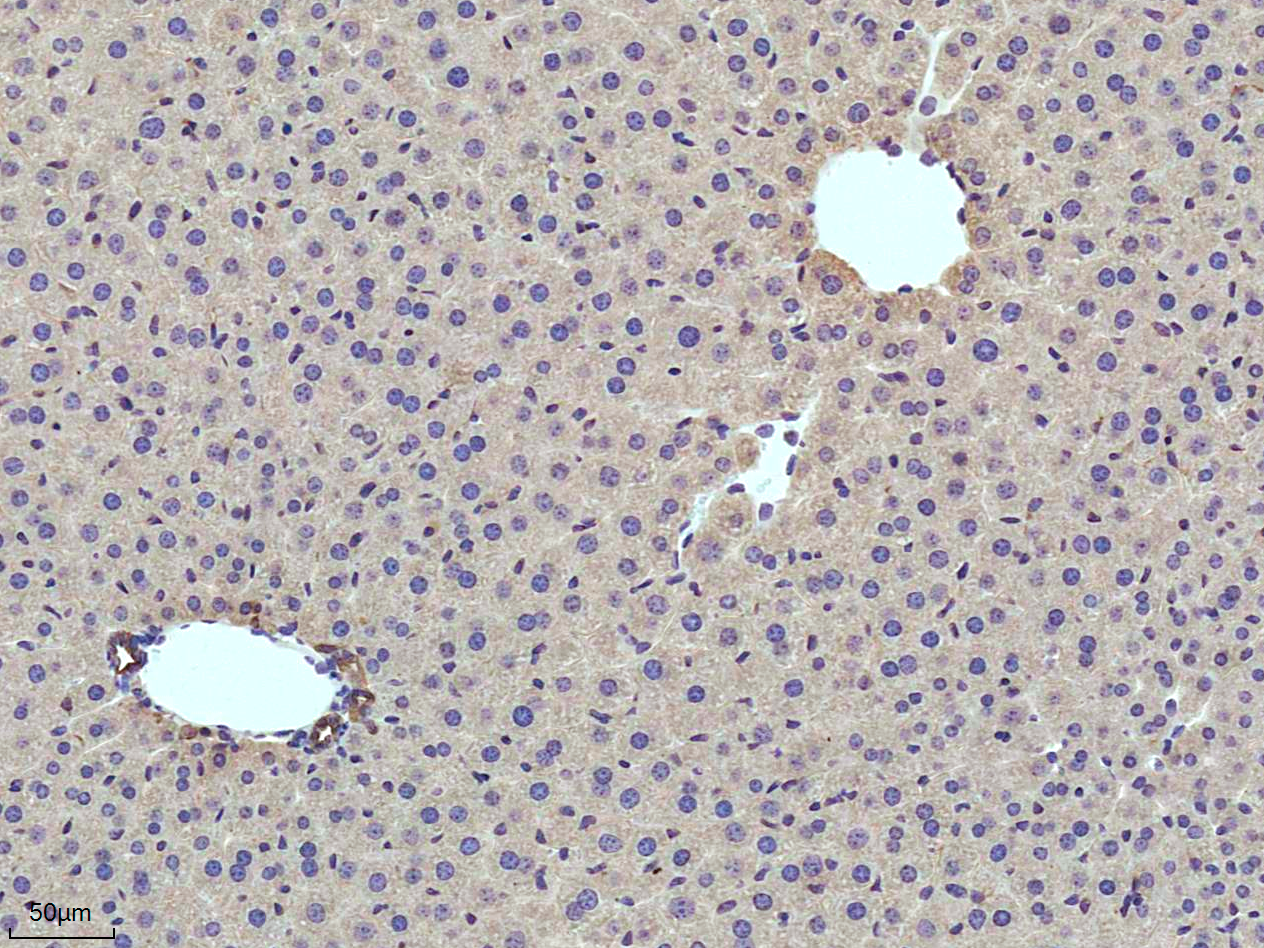

Supplement: Supplementary file 3 — Source Data for Expanded View [file EMMM-13-e14563-s002.zip › Source Data EV Figure 3/Source Data EV Figure 3D/Ck19/Wild type_Ck19.tif]

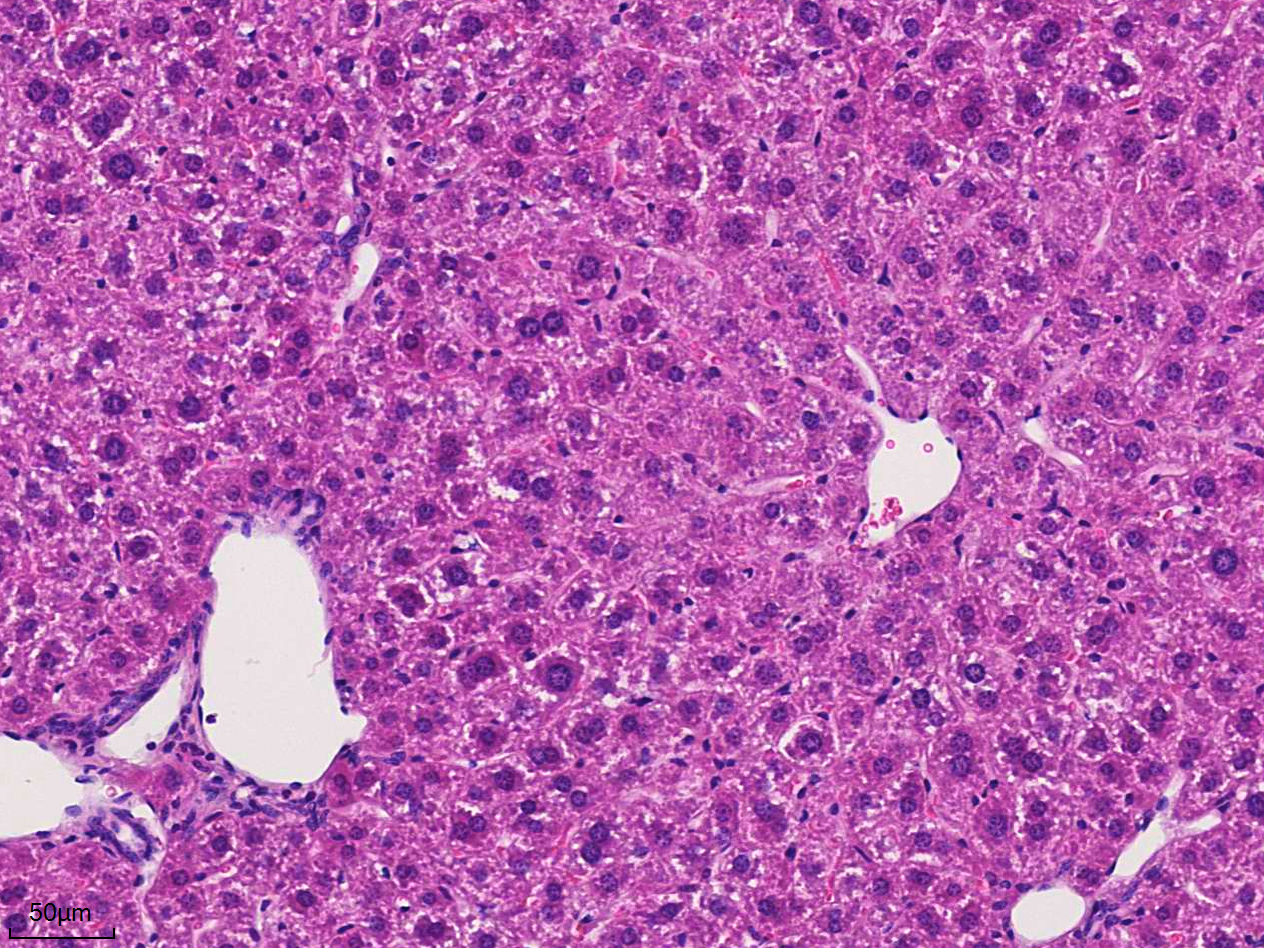

Supplement: Supplementary file 3 — Source Data for Expanded View [file EMMM-13-e14563-s002.zip › Source Data EV Figure 3/Source Data EV Figure 3D/H&E/Heterozygote_H&E.tif]

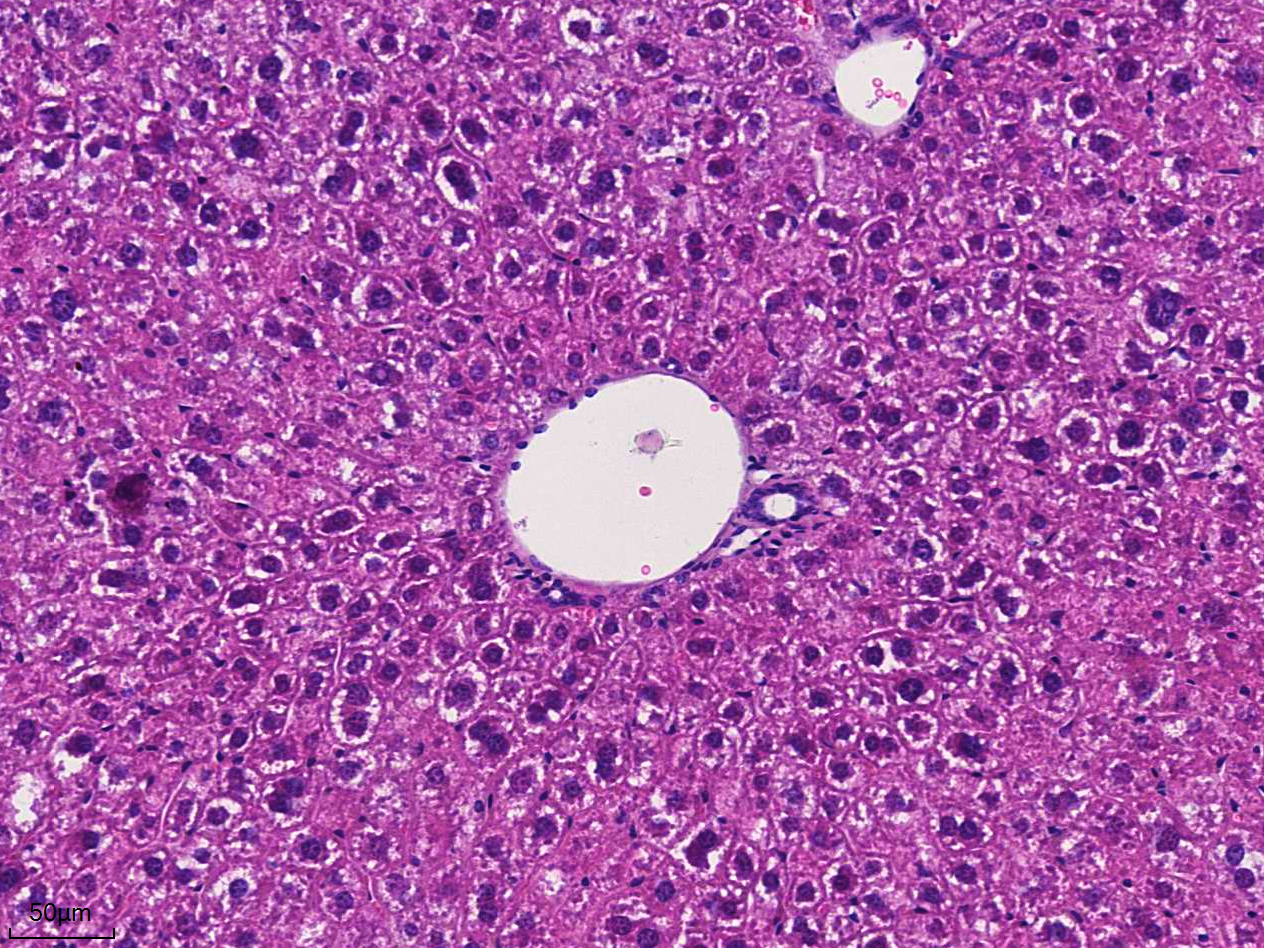

Supplement: Supplementary file 3 — Source Data for Expanded View [file EMMM-13-e14563-s002.zip › Source Data EV Figure 3/Source Data EV Figure 3D/H&E/Homozygote_H&E.tif]

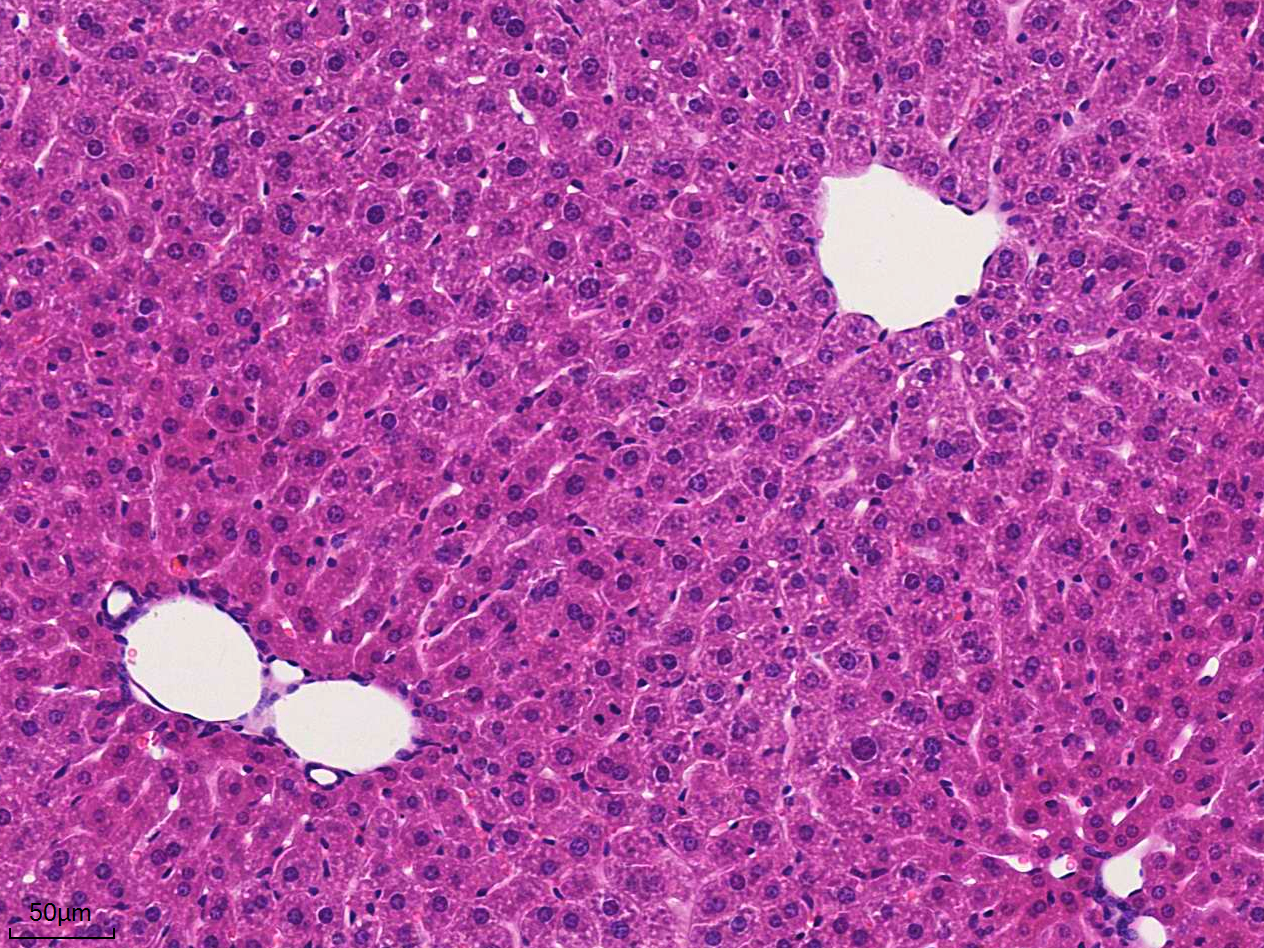

Supplement: Supplementary file 3 — Source Data for Expanded View [file EMMM-13-e14563-s002.zip › Source Data EV Figure 3/Source Data EV Figure 3D/H&E/Wild type_H&E.tif]

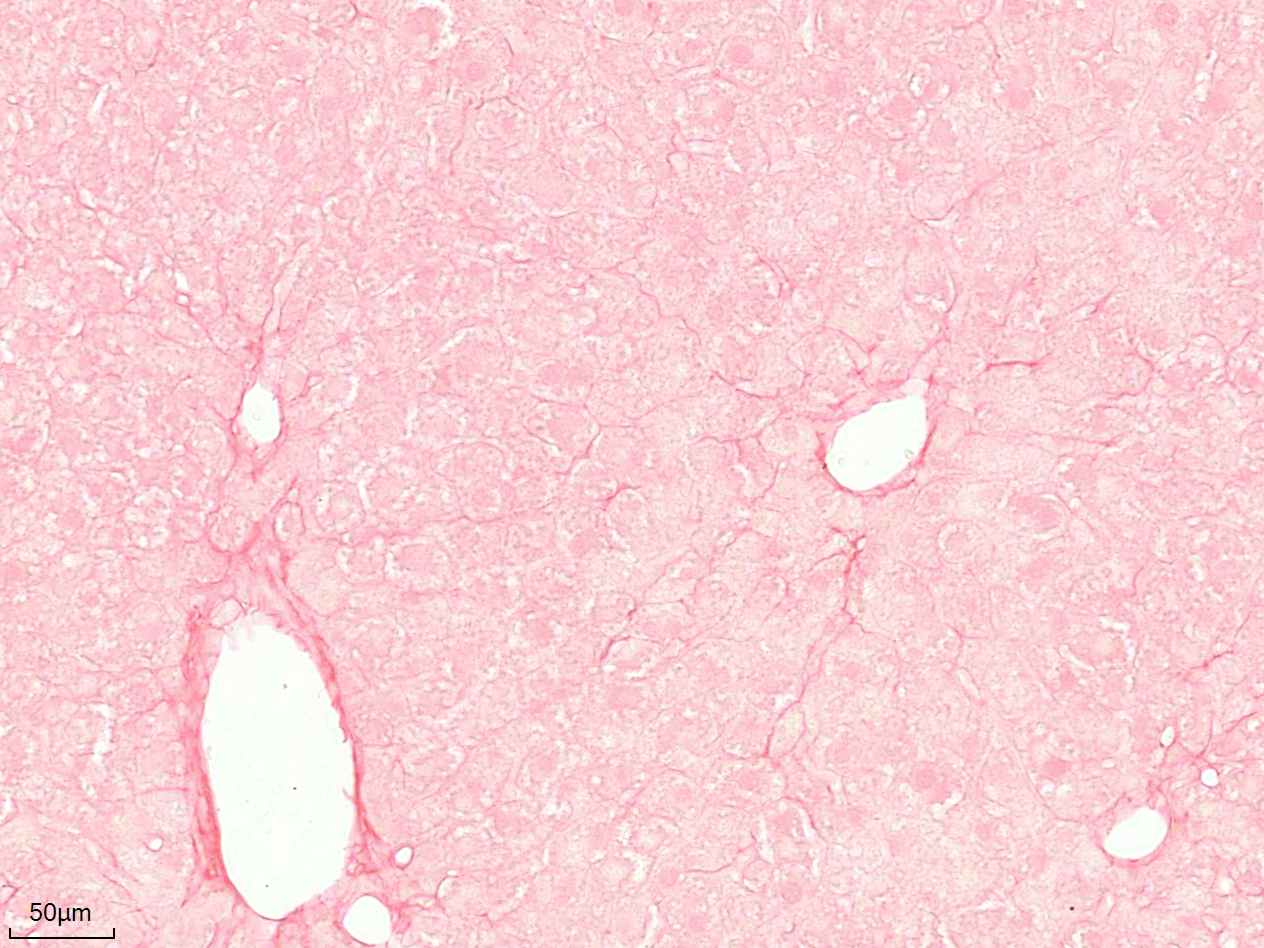

Supplement: Supplementary file 3 — Source Data for Expanded View [file EMMM-13-e14563-s002.zip › Source Data EV Figure 3/Source Data EV Figure 3D/Sirius Red/Heterozygote_Sirius Red.tif]

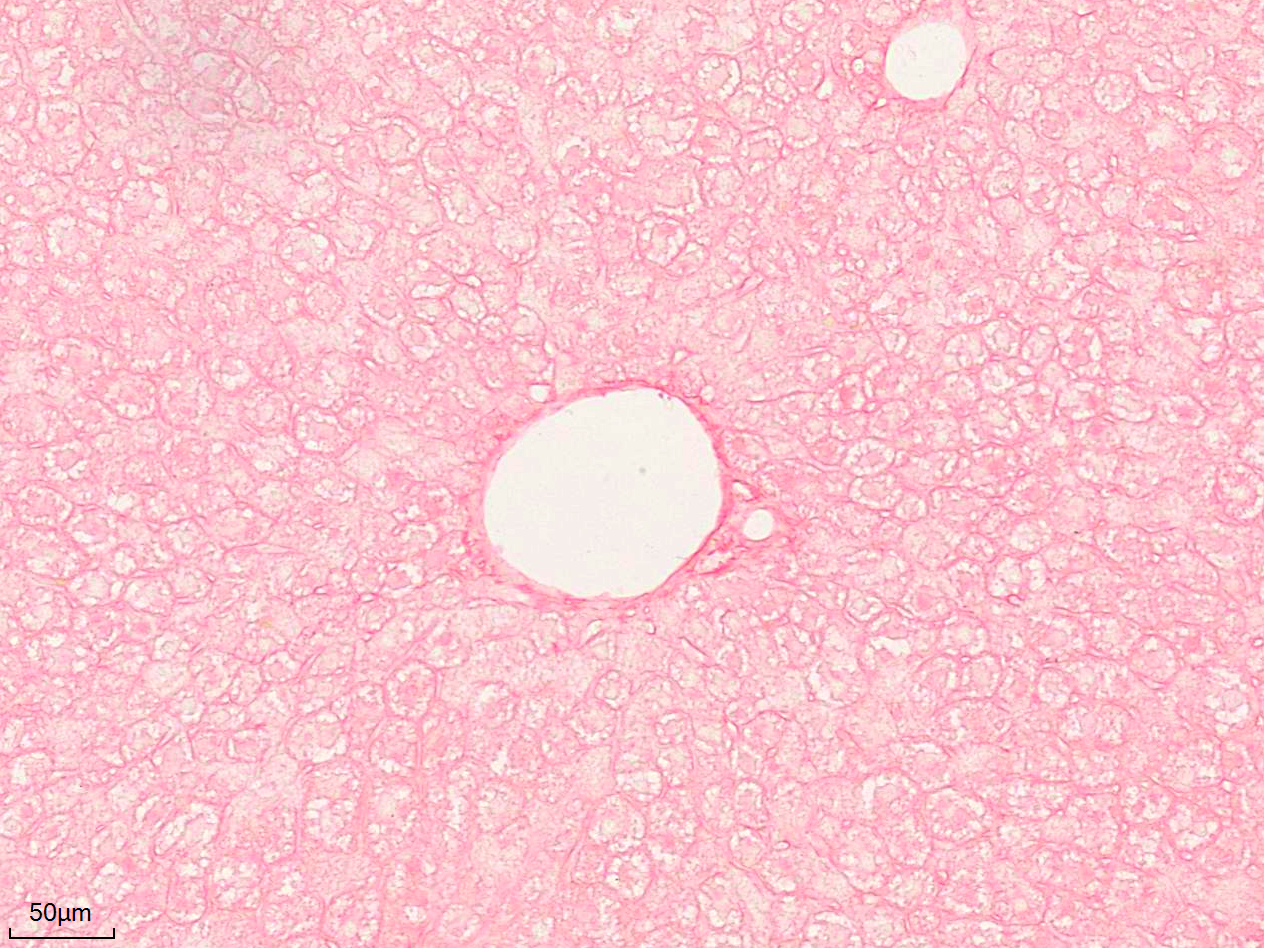

Supplement: Supplementary file 3 — Source Data for Expanded View [file EMMM-13-e14563-s002.zip › Source Data EV Figure 3/Source Data EV Figure 3D/Sirius Red/Homozygote_Sirius Red.tif]

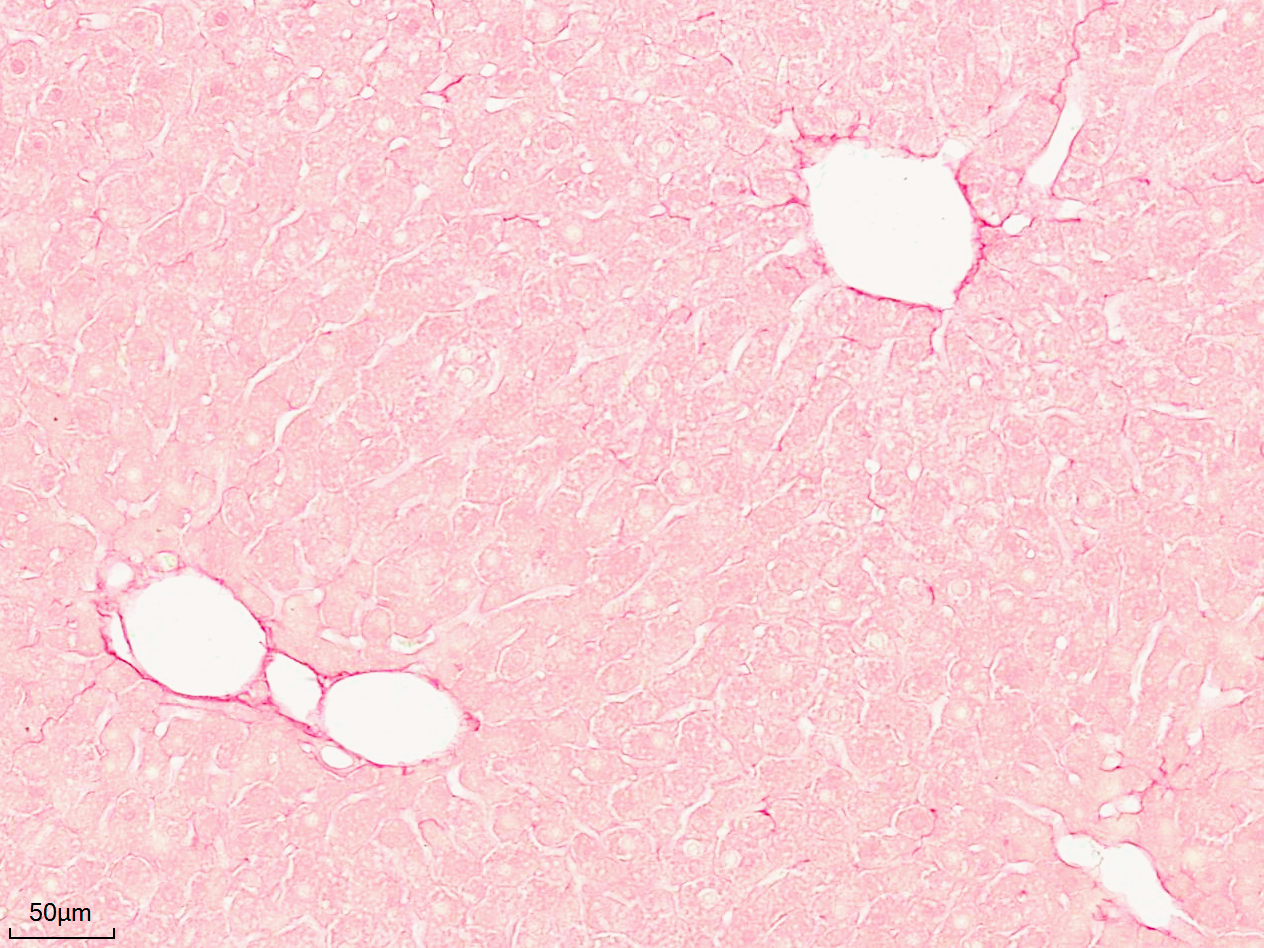

Supplement: Supplementary file 3 — Source Data for Expanded View [file EMMM-13-e14563-s002.zip › Source Data EV Figure 3/Source Data EV Figure 3D/Sirius Red/Wild type_Sirius Red.tif]

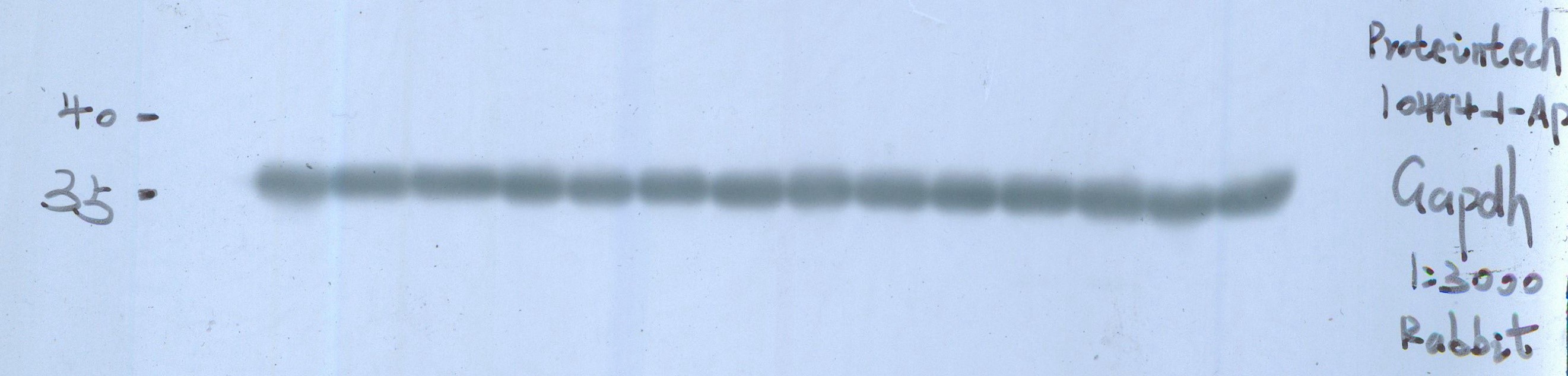

Supplement: Supplementary file 3 — Source Data for Expanded View [file EMMM-13-e14563-s002.zip › Source Data EV Figure 4/Source Data EV Figure 4A/Gapdh.jpg]

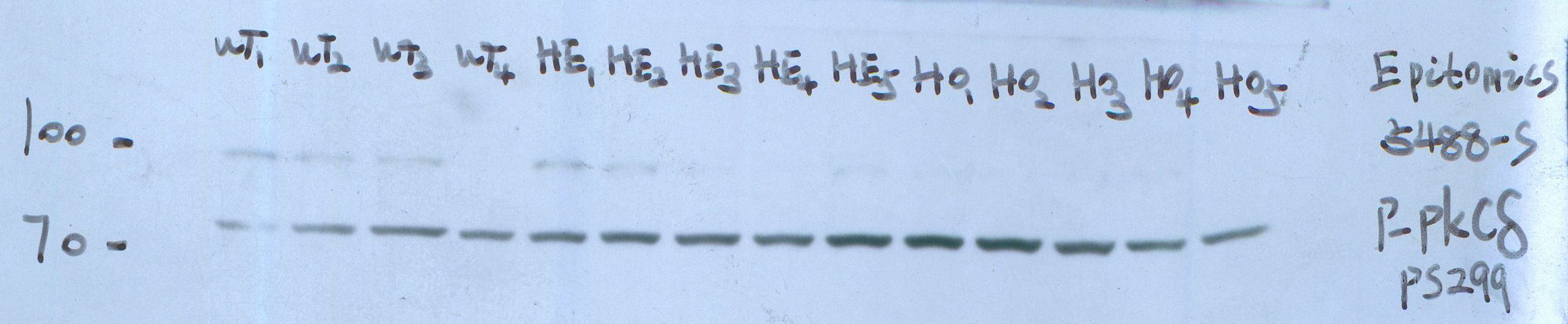

Supplement: Supplementary file 3 — Source Data for Expanded View [file EMMM-13-e14563-s002.zip › Source Data EV Figure 4/Source Data EV Figure 4A/p-PKCδ(pS299).jpg]

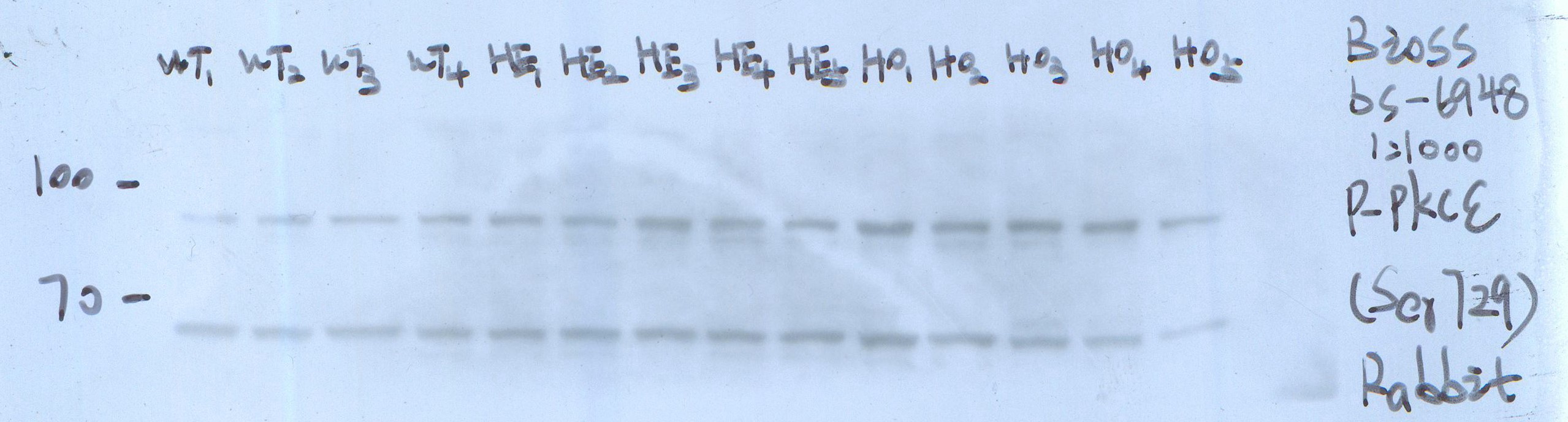

Supplement: Supplementary file 3 — Source Data for Expanded View [file EMMM-13-e14563-s002.zip › Source Data EV Figure 4/Source Data EV Figure 4A/p-PKCε(Ser729).jpg]

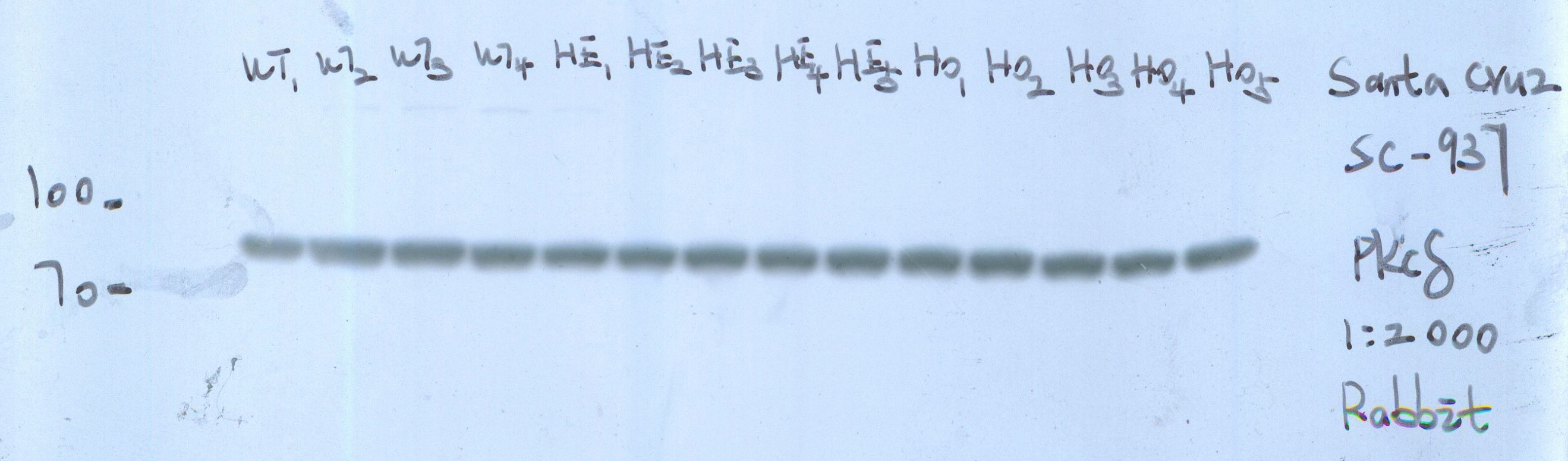

Supplement: Supplementary file 3 — Source Data for Expanded View [file EMMM-13-e14563-s002.zip › Source Data EV Figure 4/Source Data EV Figure 4A/PKCδ.jpg]

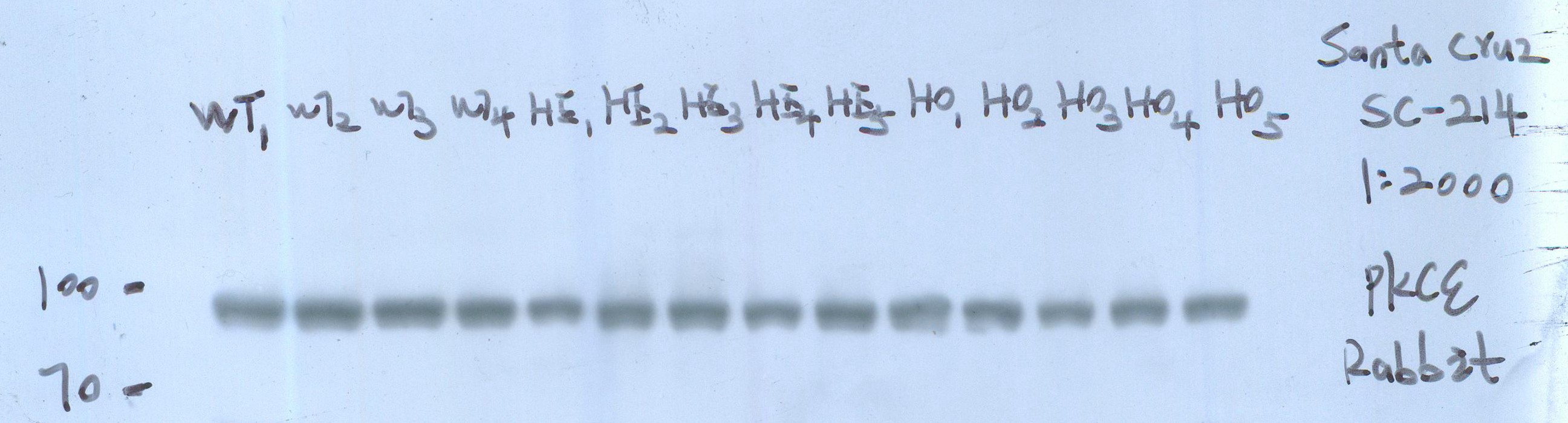

Supplement: Supplementary file 3 — Source Data for Expanded View [file EMMM-13-e14563-s002.zip › Source Data EV Figure 4/Source Data EV Figure 4A/PKCε.jpg]

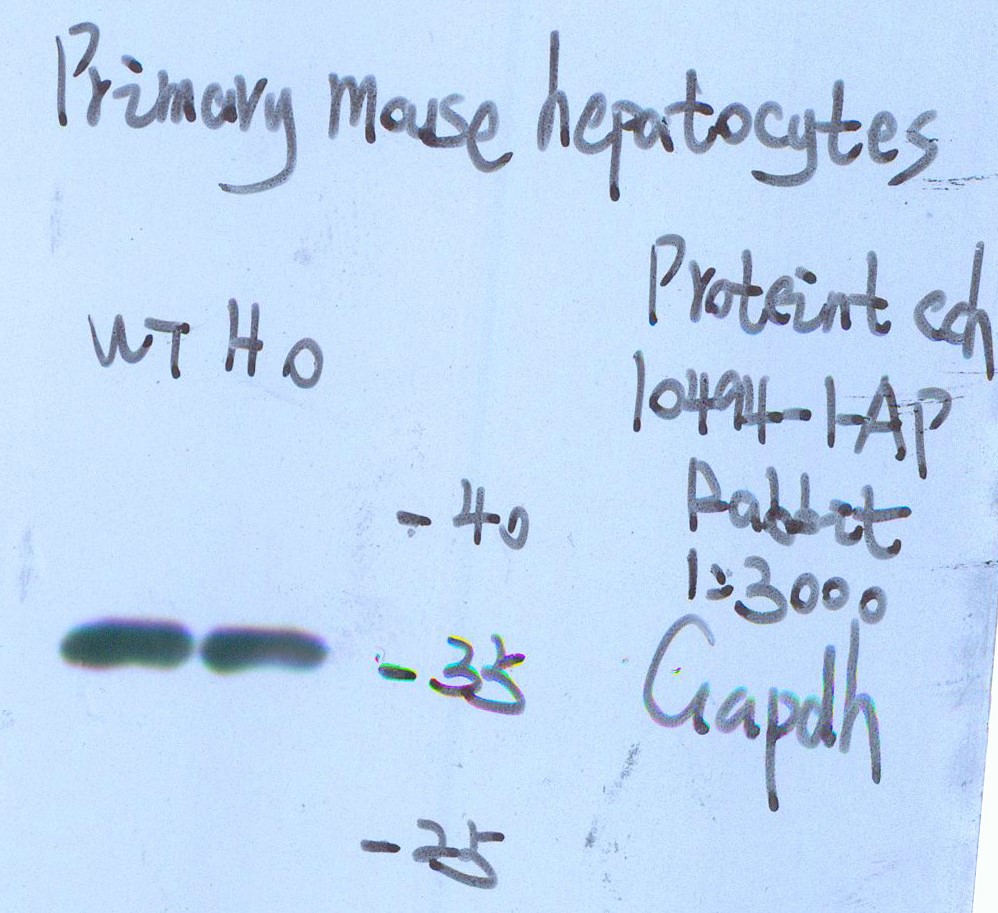

Supplement: Supplementary file 3 — Source Data for Expanded View [file EMMM-13-e14563-s002.zip › Source Data EV Figure 4/Source Data EV Figure 4B/Gapdh.jpg]

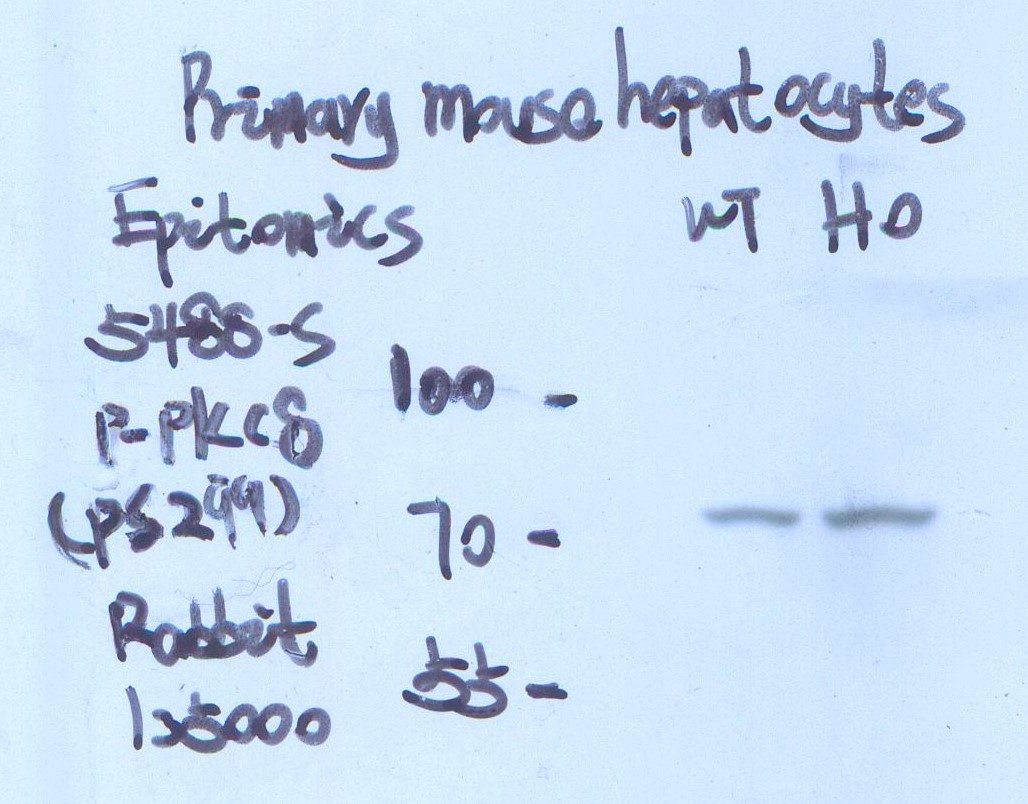

Supplement: Supplementary file 3 — Source Data for Expanded View [file EMMM-13-e14563-s002.zip › Source Data EV Figure 4/Source Data EV Figure 4B/p-PKCδ(pS299).jpg]

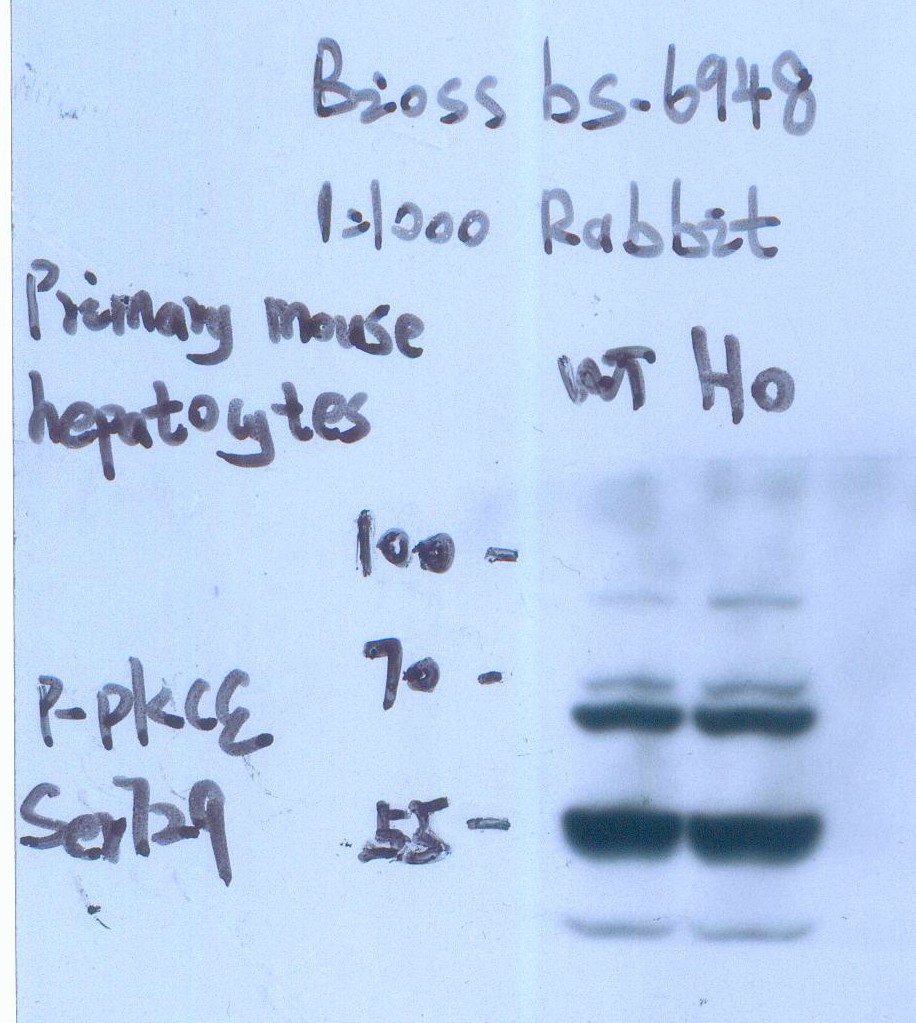

Supplement: Supplementary file 3 — Source Data for Expanded View [file EMMM-13-e14563-s002.zip › Source Data EV Figure 4/Source Data EV Figure 4B/p-PKCε(Ser729).jpg]

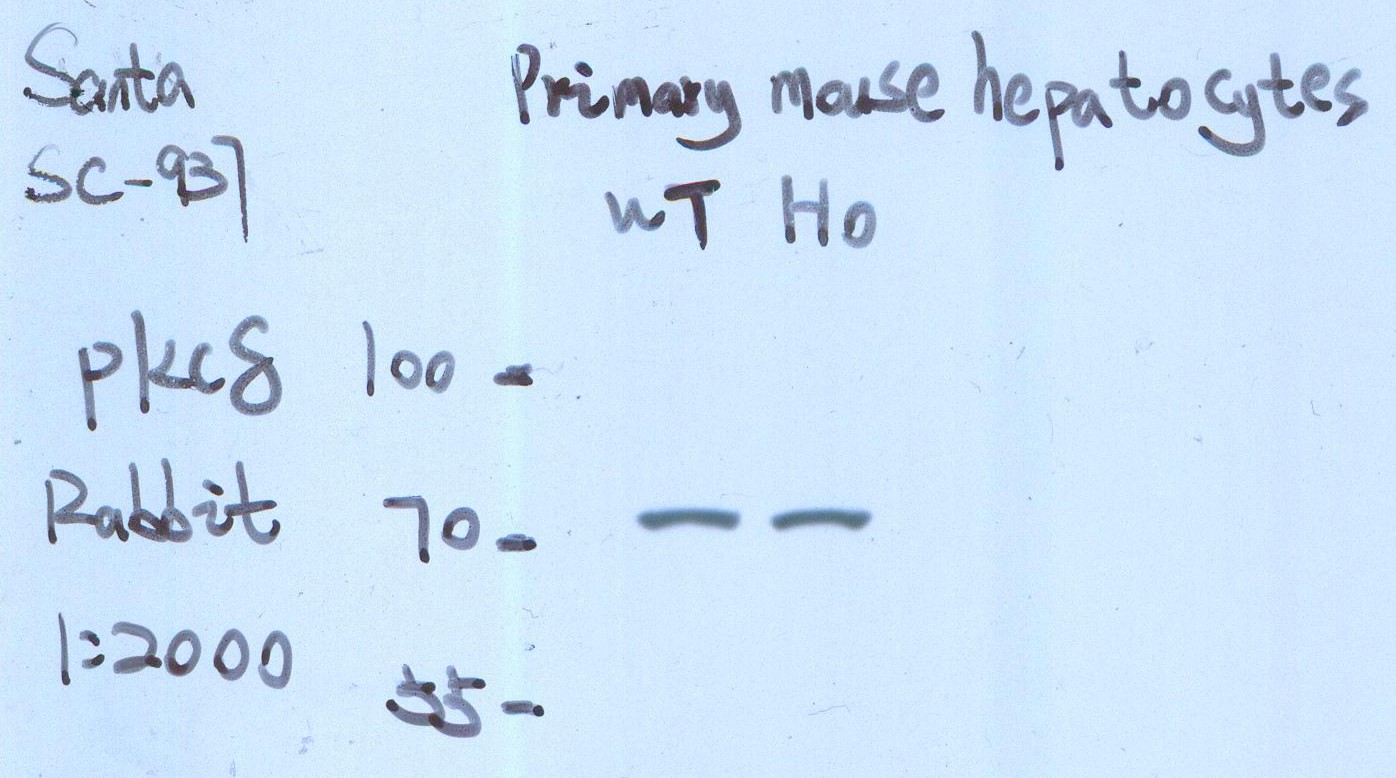

Supplement: Supplementary file 3 — Source Data for Expanded View [file EMMM-13-e14563-s002.zip › Source Data EV Figure 4/Source Data EV Figure 4B/PKCδ.jpg]

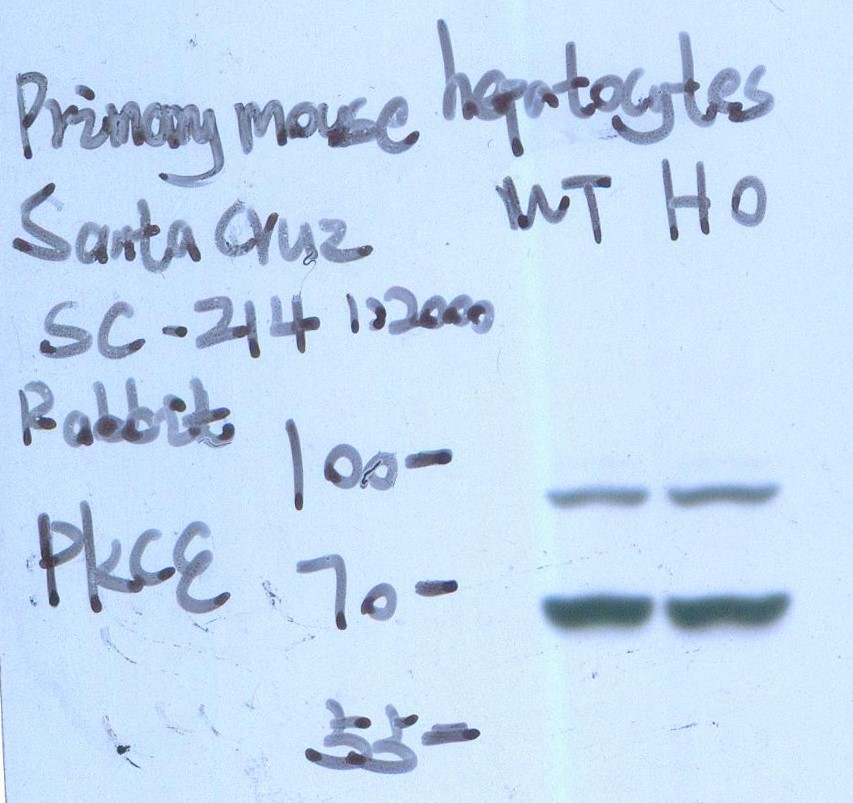

Supplement: Supplementary file 3 — Source Data for Expanded View [file EMMM-13-e14563-s002.zip › Source Data EV Figure 4/Source Data EV Figure 4B/PKCε.jpg]

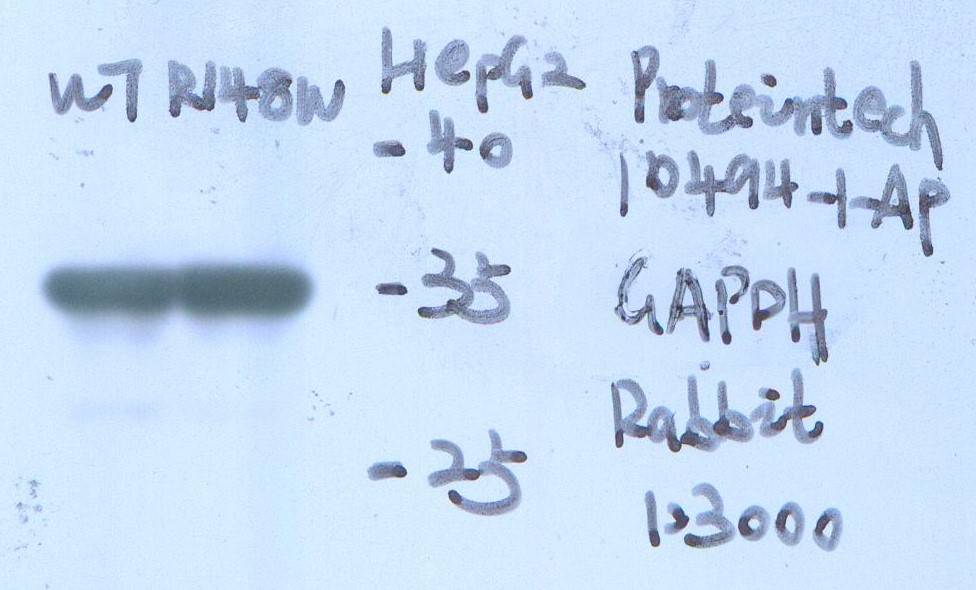

Supplement: Supplementary file 3 — Source Data for Expanded View [file EMMM-13-e14563-s002.zip › Source Data EV Figure 4/Source Data EV Figure 4C/GAPDH.jpg]

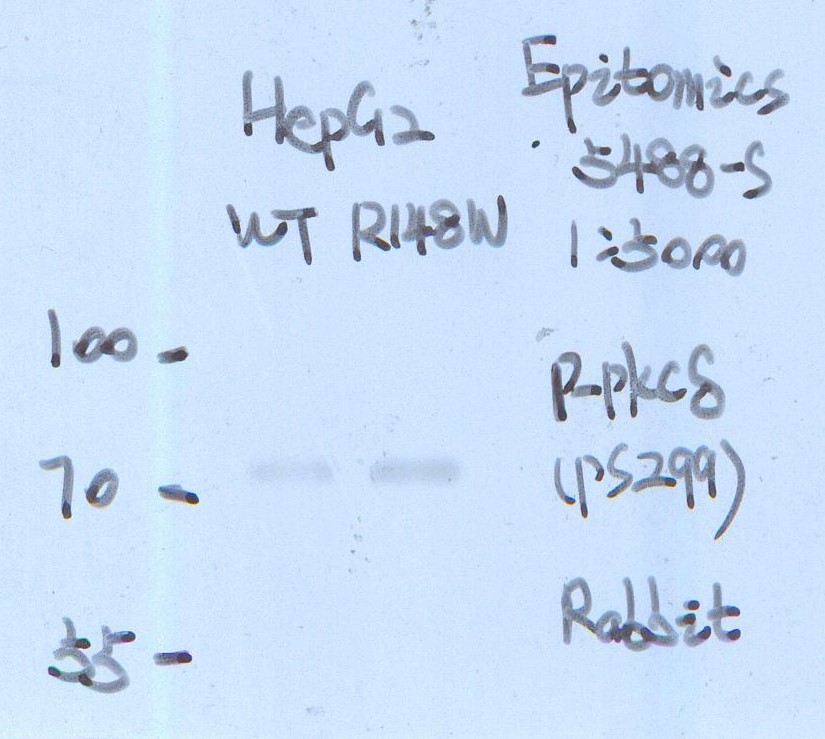

Supplement: Supplementary file 3 — Source Data for Expanded View [file EMMM-13-e14563-s002.zip › Source Data EV Figure 4/Source Data EV Figure 4C/p-PKCδ(pS299).jpg]

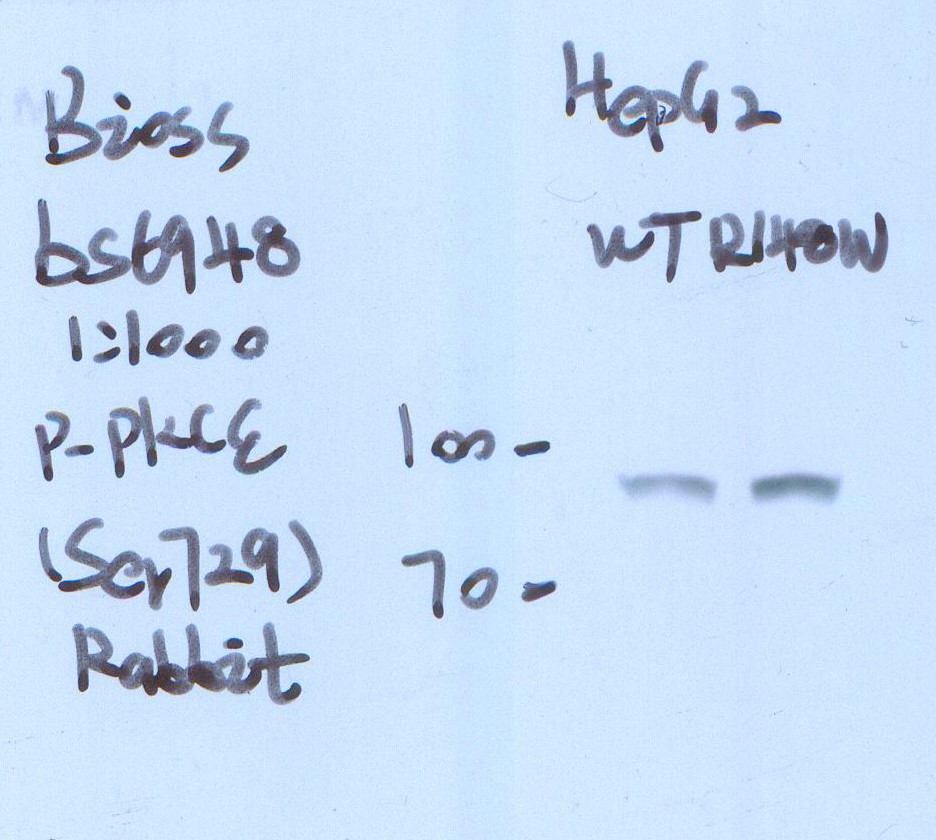

Supplement: Supplementary file 3 — Source Data for Expanded View [file EMMM-13-e14563-s002.zip › Source Data EV Figure 4/Source Data EV Figure 4C/p-PKCε(Ser729).jpg]

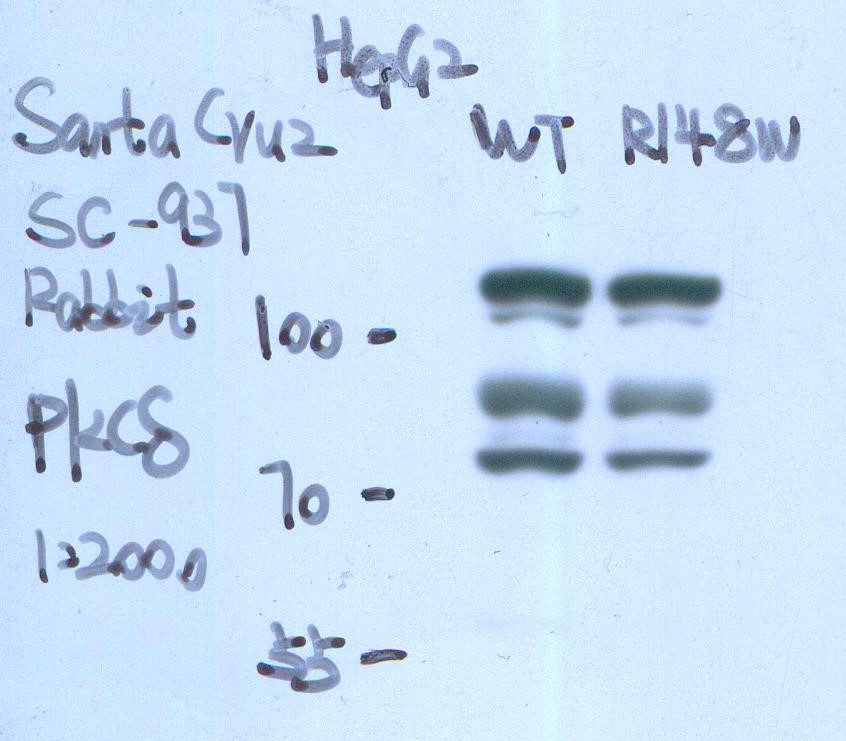

Supplement: Supplementary file 3 — Source Data for Expanded View [file EMMM-13-e14563-s002.zip › Source Data EV Figure 4/Source Data EV Figure 4C/PKCδ.jpg]

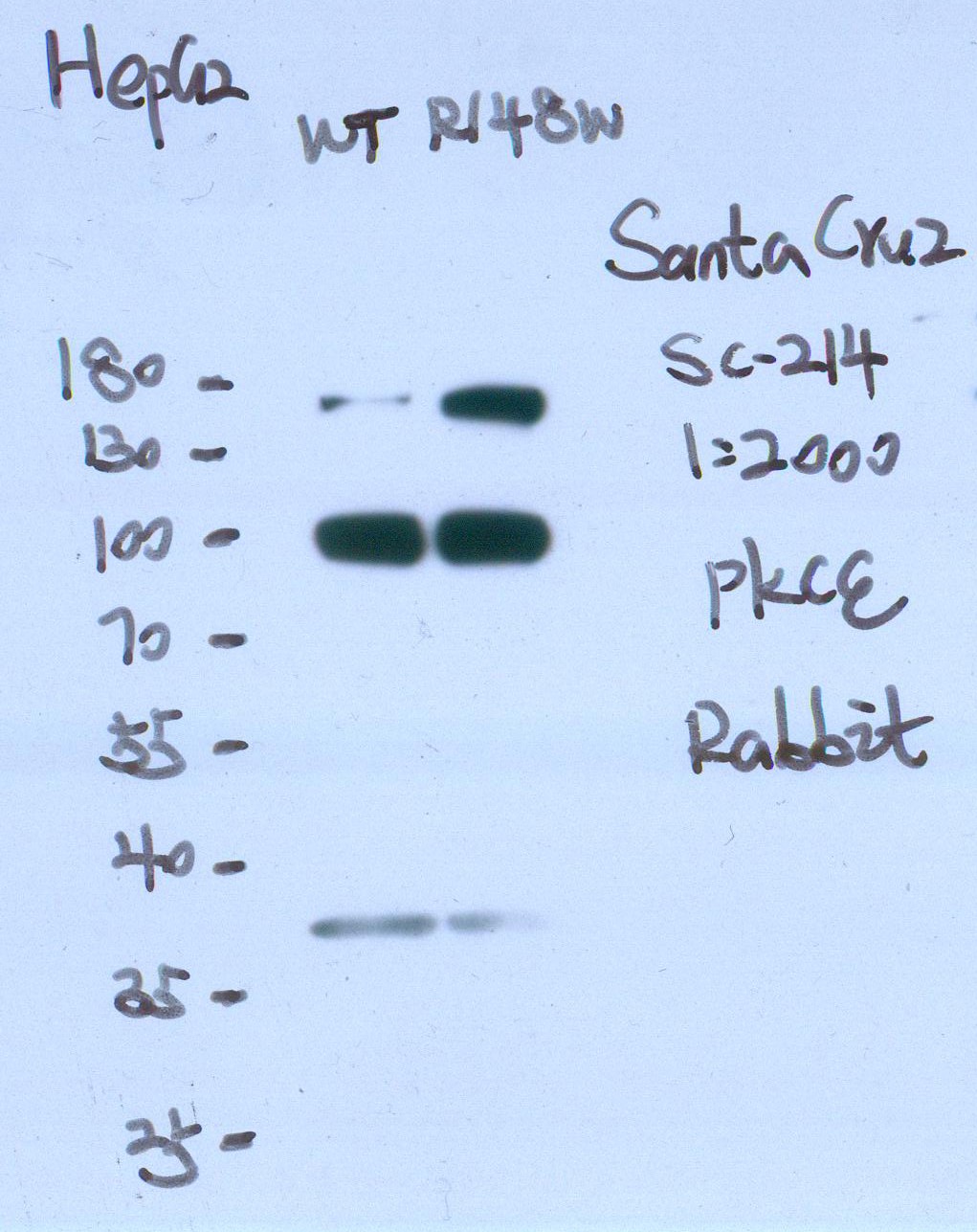

Supplement: Supplementary file 3 — Source Data for Expanded View [file EMMM-13-e14563-s002.zip › Source Data EV Figure 4/Source Data EV Figure 4C/PKCε.jpg]

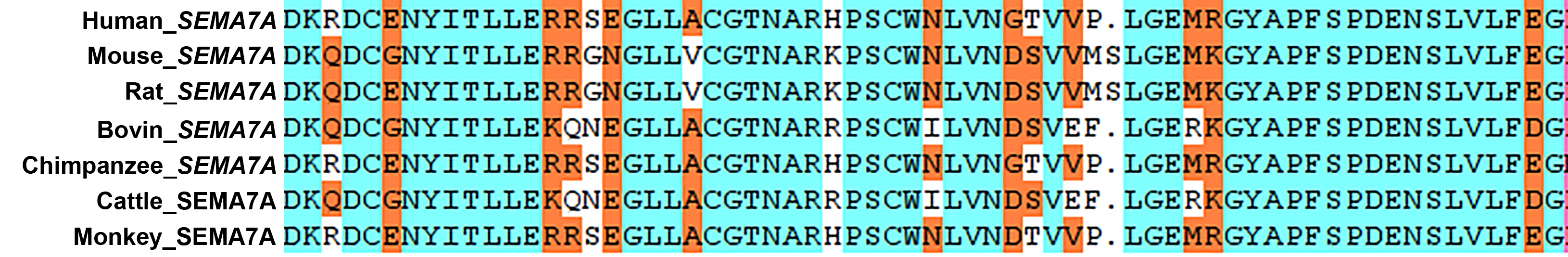

Supplement: Supplementary file 4 — Source Data for Figure 1 [file EMMM-13-e14563-s003.zip › 1C Multiple-Alignment-SEMA7A.tif]

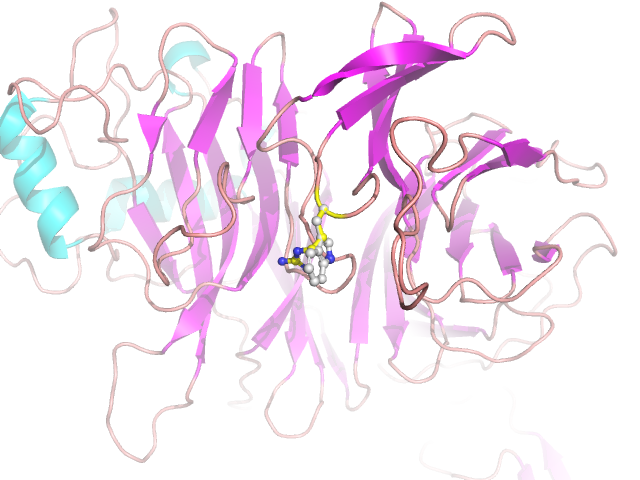

Supplement: Supplementary file 4 — Source Data for Figure 1 [file EMMM-13-e14563-s003.zip › 1D cartoon_structure.png]

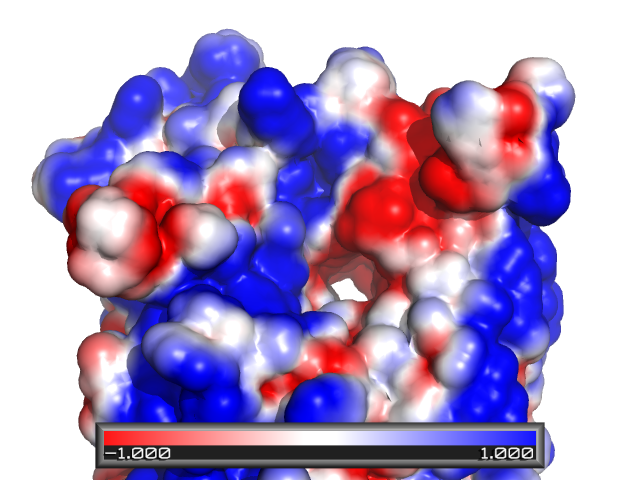

Supplement: Supplementary file 4 — Source Data for Figure 1 [file EMMM-13-e14563-s003.zip › 1E R148 WT.png]

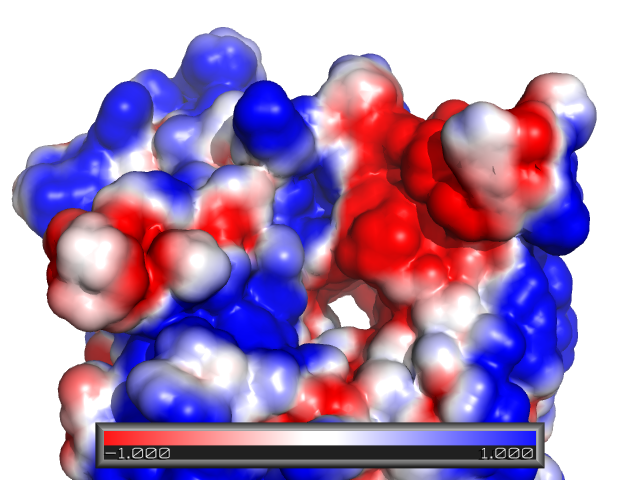

Supplement: Supplementary file 4 — Source Data for Figure 1 [file EMMM-13-e14563-s003.zip › 1F W148 Mutation.png]

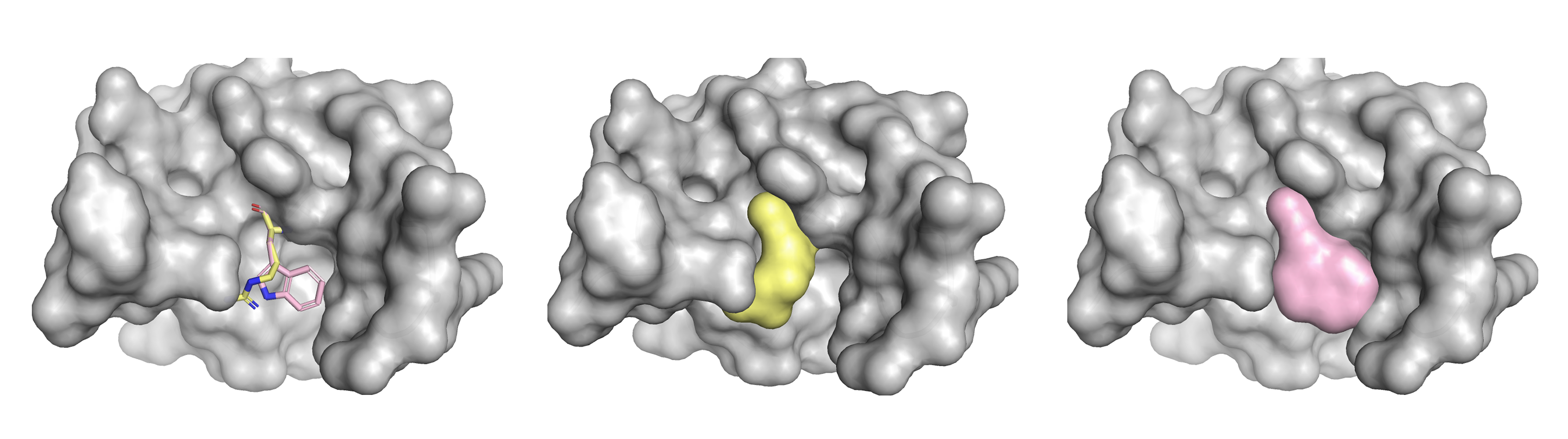

Supplement: Supplementary file 4 — Source Data for Figure 1 [file EMMM-13-e14563-s003.zip › SEMA7A_R148W.tif]

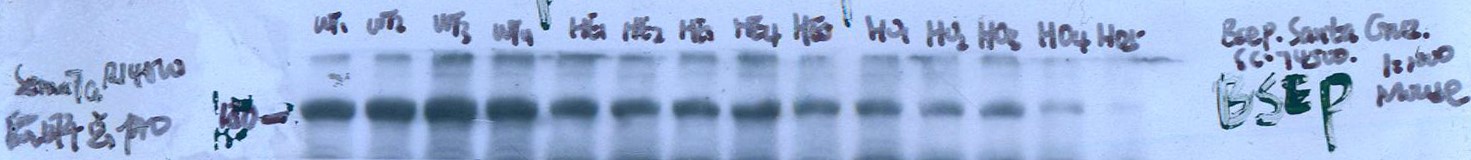

Supplement: Supplementary file 5 — Source Data for Figure 2 [file EMMM-13-e14563-s007.zip › Source Data Figure 2D/Bsep.jpg]

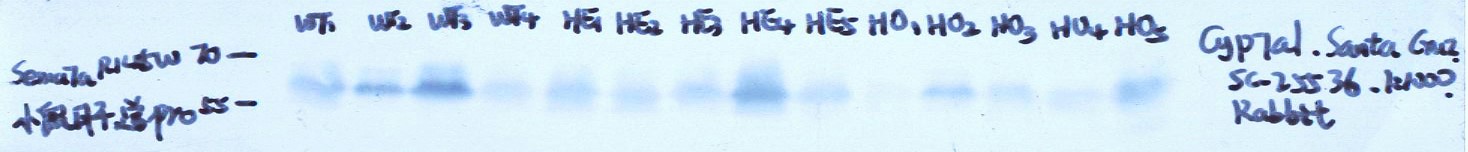

Supplement: Supplementary file 5 — Source Data for Figure 2 [file EMMM-13-e14563-s007.zip › Source Data Figure 2D/Cyp7a1.jpg]

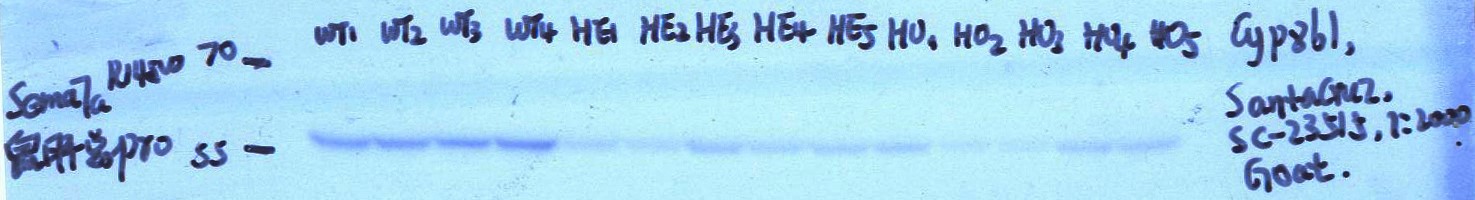

Supplement: Supplementary file 5 — Source Data for Figure 2 [file EMMM-13-e14563-s007.zip › Source Data Figure 2D/Cyp8b1.jpg]

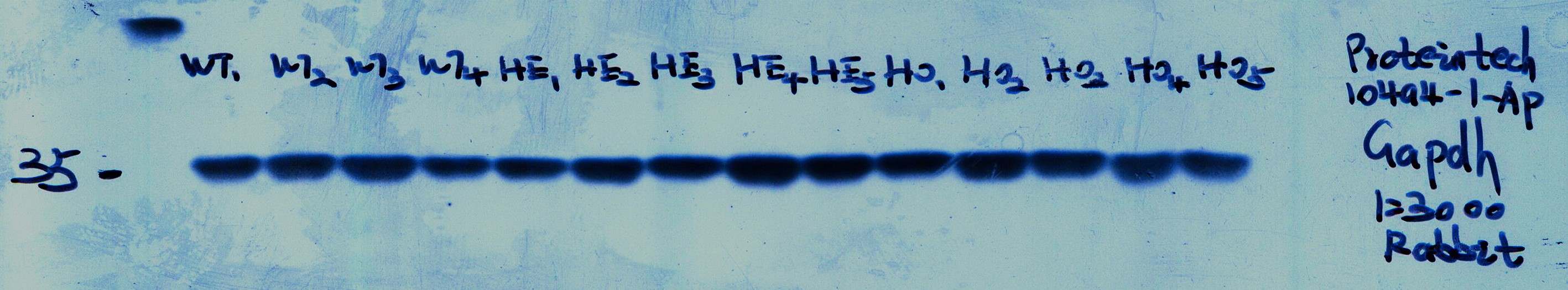

Supplement: Supplementary file 5 — Source Data for Figure 2 [file EMMM-13-e14563-s007.zip › Source Data Figure 2D/Gapdh_001.jpg]

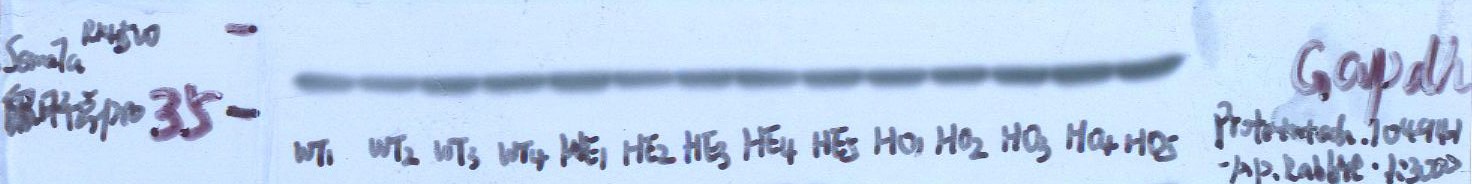

Supplement: Supplementary file 5 — Source Data for Figure 2 [file EMMM-13-e14563-s007.zip › Source Data Figure 2D/Gapdh_002.jpg]

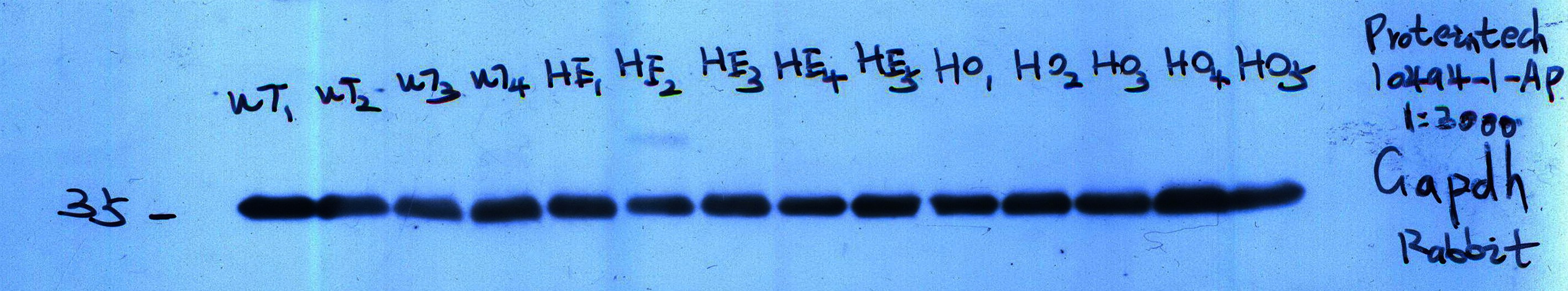

Supplement: Supplementary file 5 — Source Data for Figure 2 [file EMMM-13-e14563-s007.zip › Source Data Figure 2D/Gapdh_003.jpg]

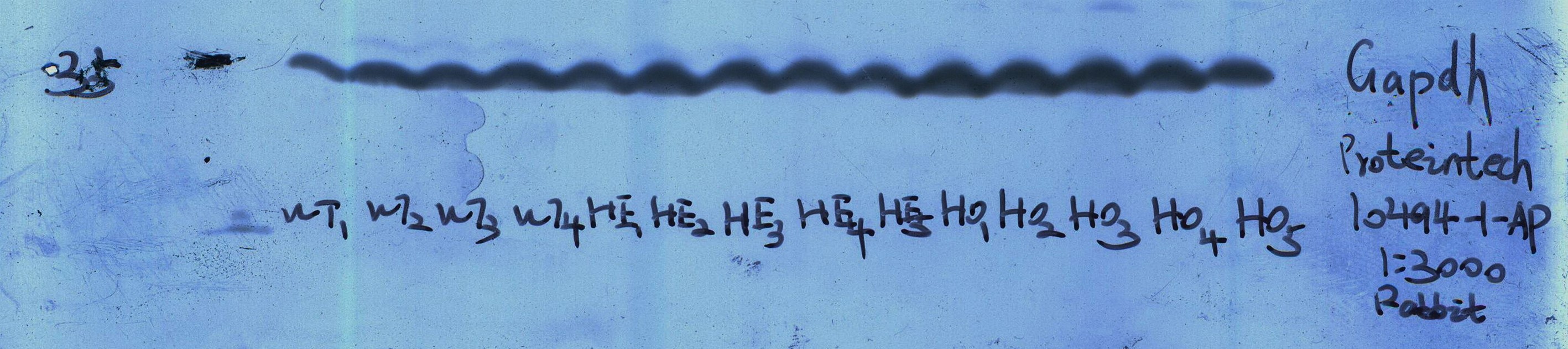

Supplement: Supplementary file 5 — Source Data for Figure 2 [file EMMM-13-e14563-s007.zip › Source Data Figure 2D/Gapdh_004.jpg]

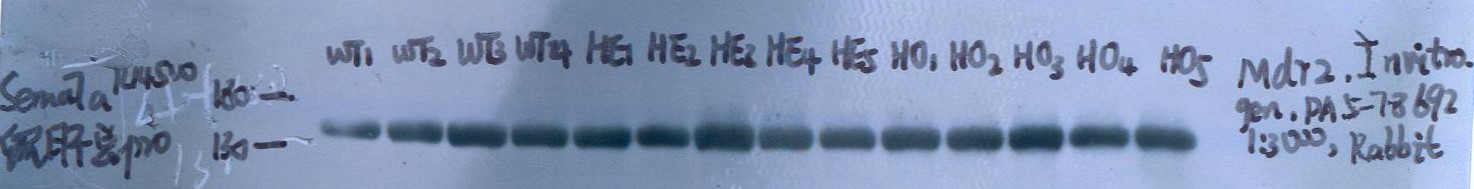

Supplement: Supplementary file 5 — Source Data for Figure 2 [file EMMM-13-e14563-s007.zip › Source Data Figure 2D/Mdr2.jpg]

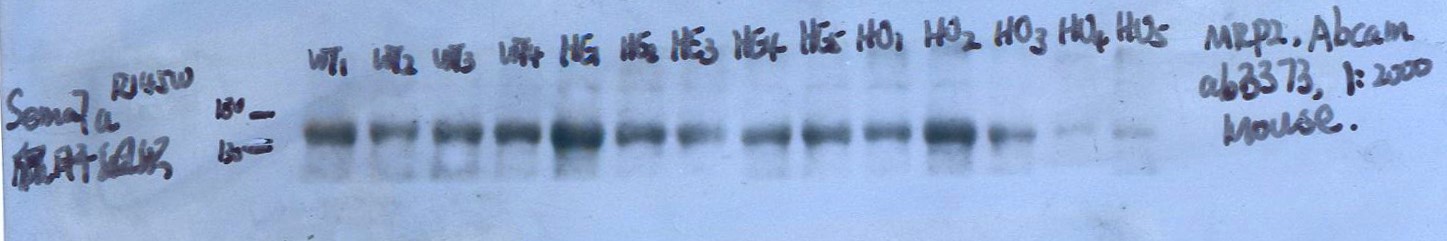

Supplement: Supplementary file 5 — Source Data for Figure 2 [file EMMM-13-e14563-s007.zip › Source Data Figure 2D/Mrp2.jpg]

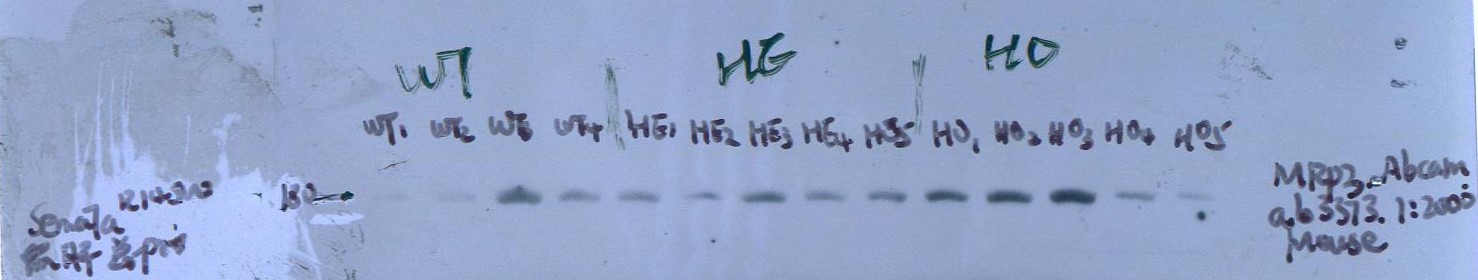

Supplement: Supplementary file 5 — Source Data for Figure 2 [file EMMM-13-e14563-s007.zip › Source Data Figure 2D/Mrp3.jpg]

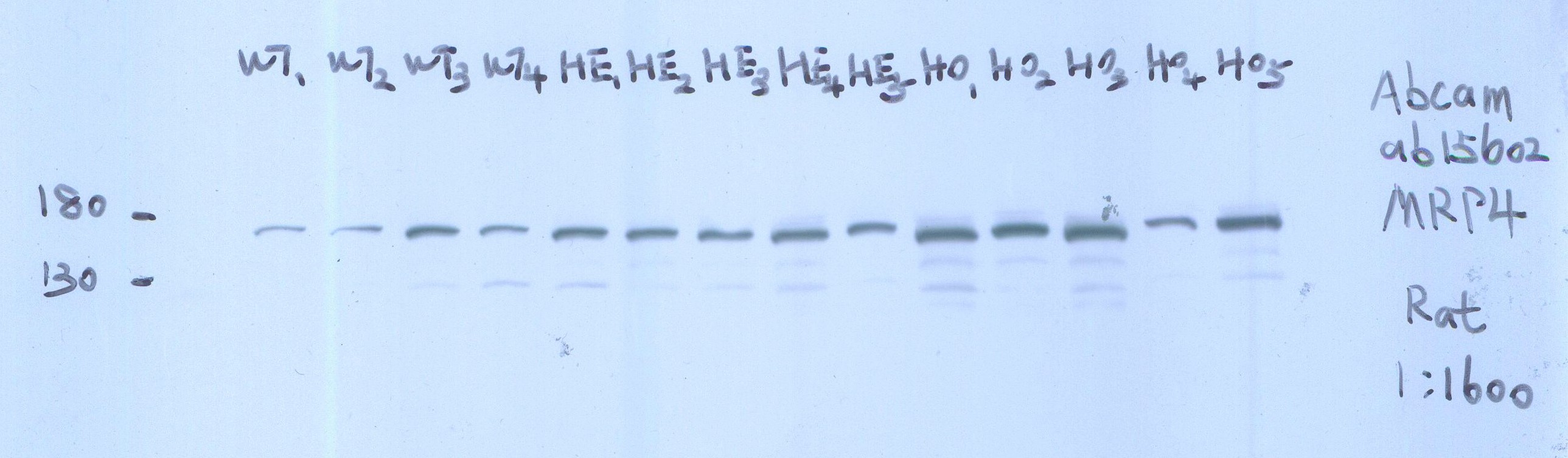

Supplement: Supplementary file 5 — Source Data for Figure 2 [file EMMM-13-e14563-s007.zip › Source Data Figure 2D/Mrp4.jpg]

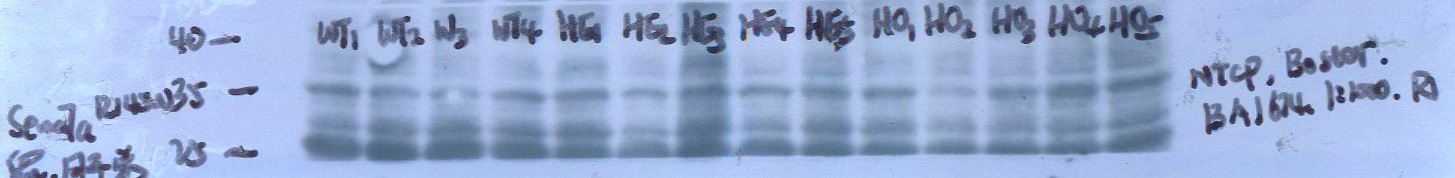

Supplement: Supplementary file 5 — Source Data for Figure 2 [file EMMM-13-e14563-s007.zip › Source Data Figure 2D/Ntcp.jpg]

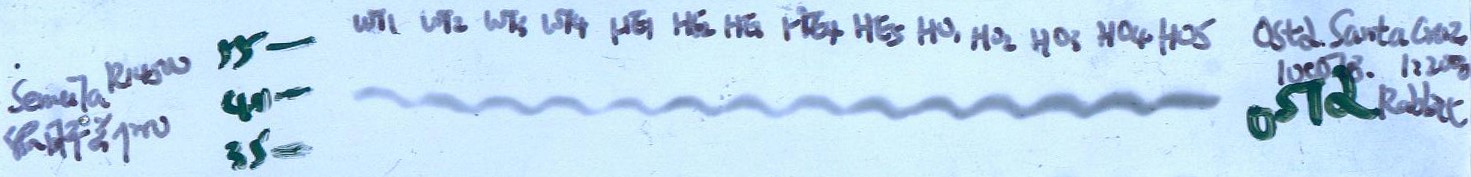

Supplement: Supplementary file 5 — Source Data for Figure 2 [file EMMM-13-e14563-s007.zip › Source Data Figure 2D/Ostα.jpg]

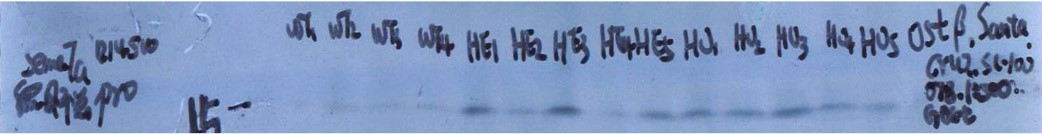

Supplement: Supplementary file 5 — Source Data for Figure 2 [file EMMM-13-e14563-s007.zip › Source Data Figure 2D/Ostβ.jpg]

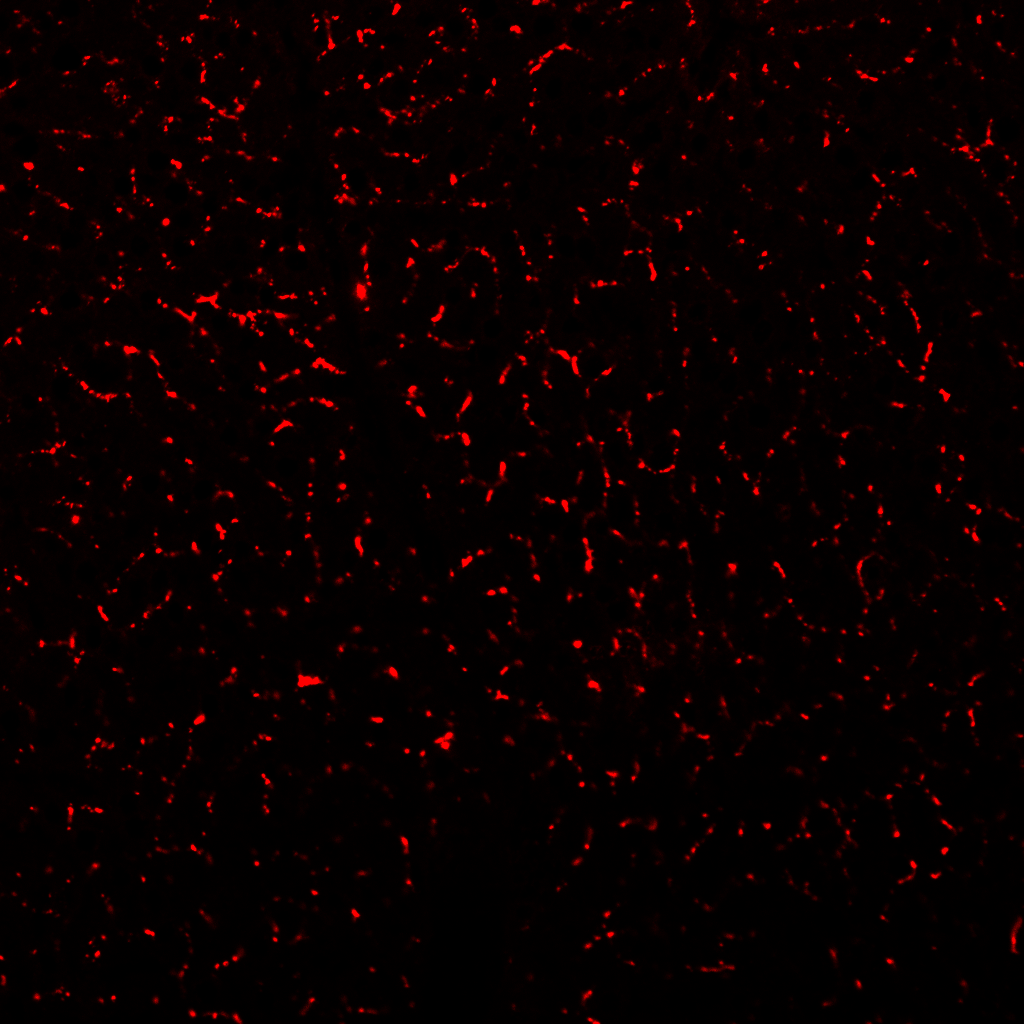

Supplement: Supplementary file 5 — Source Data for Figure 2 [file EMMM-13-e14563-s007.zip › Source Data Figure 2E/Homozygote/Homozygote_Bsep.tif]

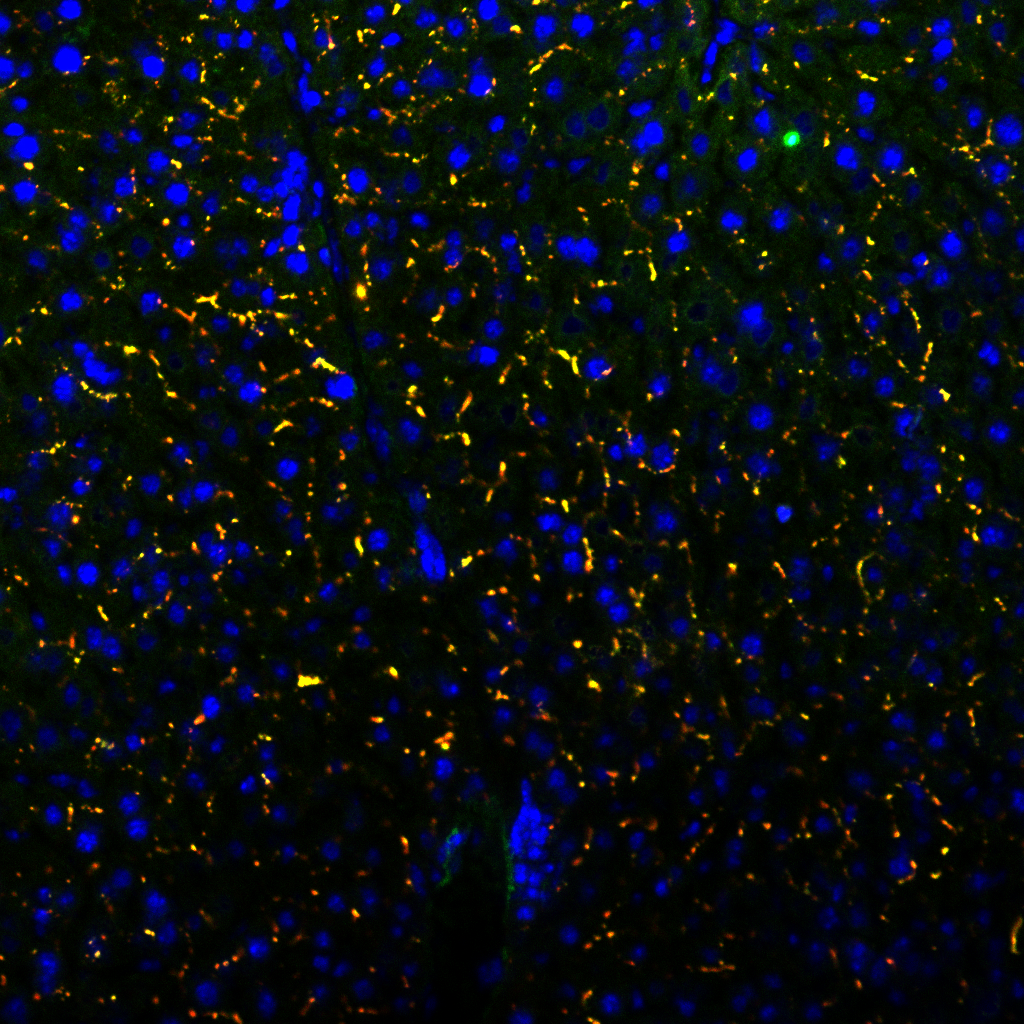

Supplement: Supplementary file 5 — Source Data for Figure 2 [file EMMM-13-e14563-s007.zip › Source Data Figure 2E/Homozygote/Homozygote_Merge.tif]

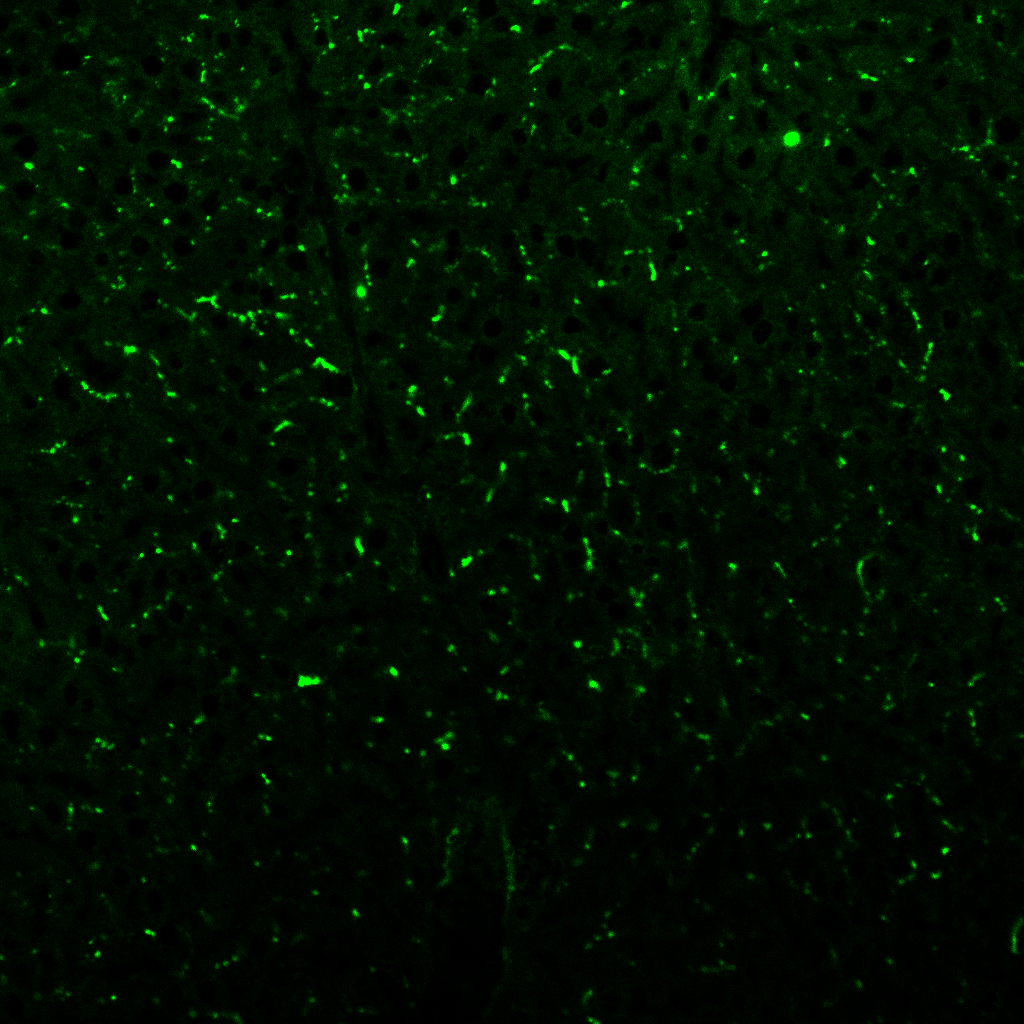

Supplement: Supplementary file 5 — Source Data for Figure 2 [file EMMM-13-e14563-s007.zip › Source Data Figure 2E/Homozygote/Homozygote_Mrp2.tif]

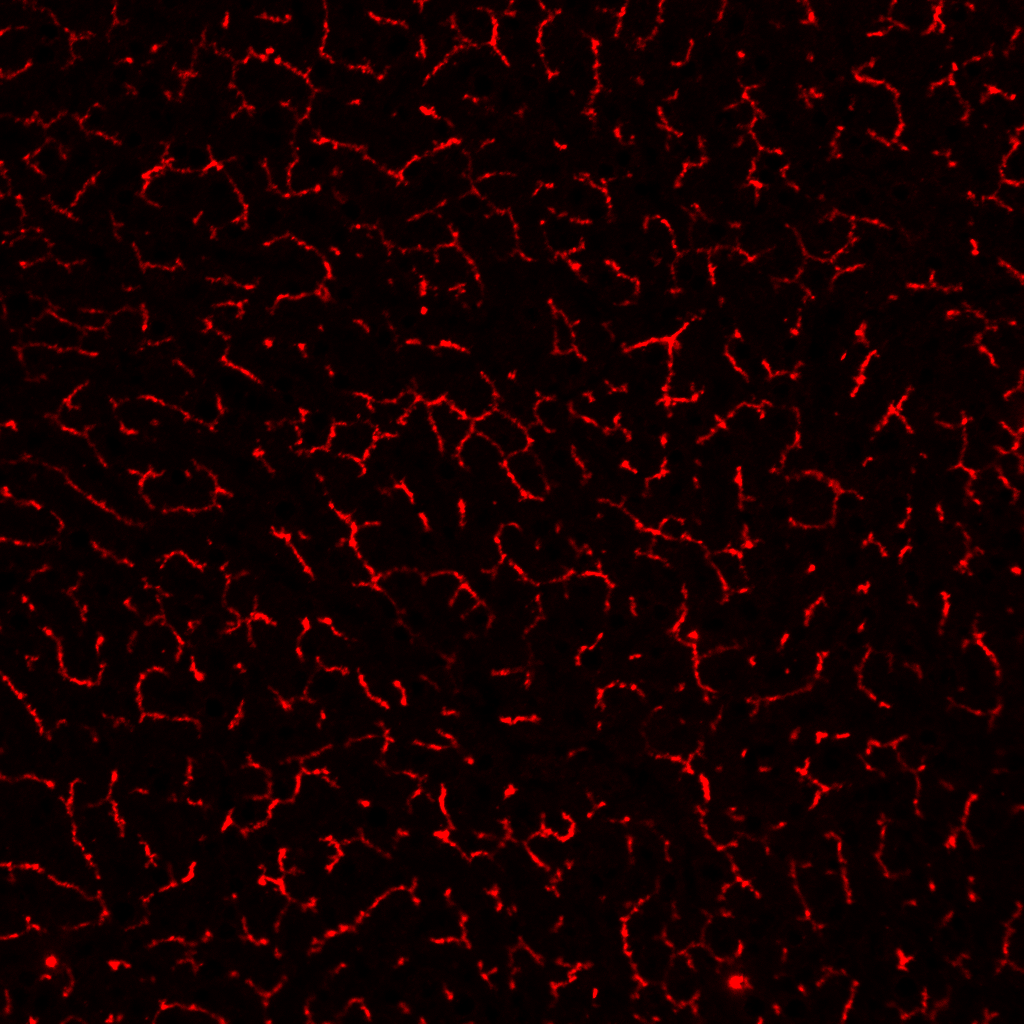

Supplement: Supplementary file 5 — Source Data for Figure 2 [file EMMM-13-e14563-s007.zip › Source Data Figure 2E/Wild type/Wild type_Bsep.tif]

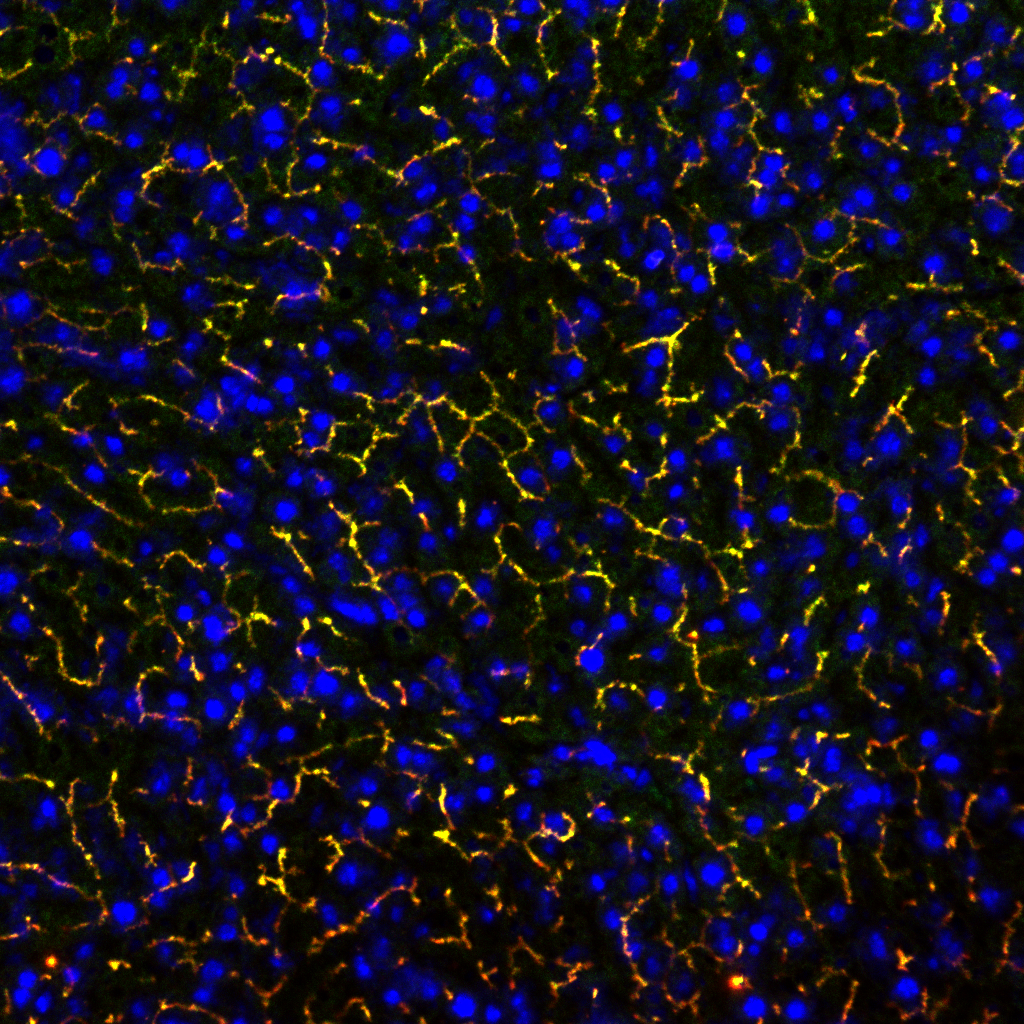

Supplement: Supplementary file 5 — Source Data for Figure 2 [file EMMM-13-e14563-s007.zip › Source Data Figure 2E/Wild type/Wild type_Merge.tif]

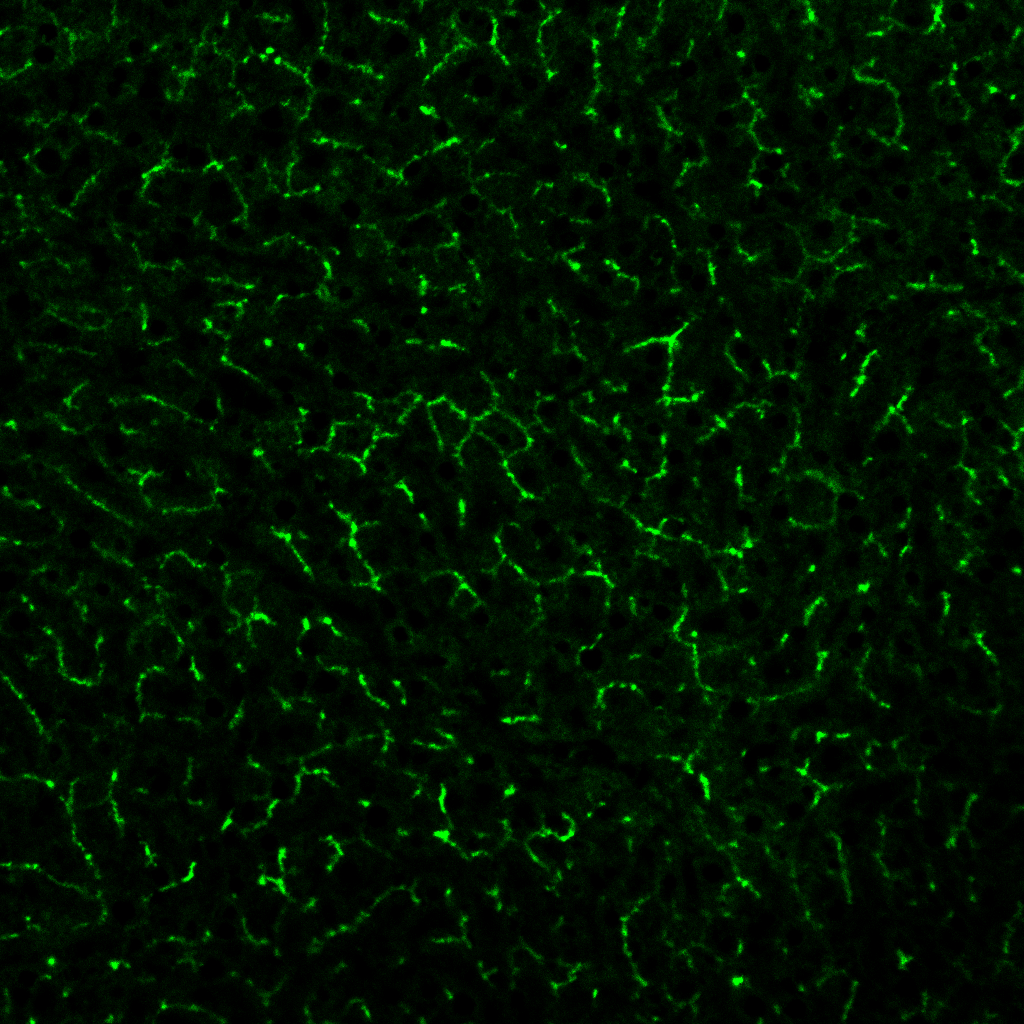

Supplement: Supplementary file 5 — Source Data for Figure 2 [file EMMM-13-e14563-s007.zip › Source Data Figure 2E/Wild type/Wild type_Mrp2.tif]

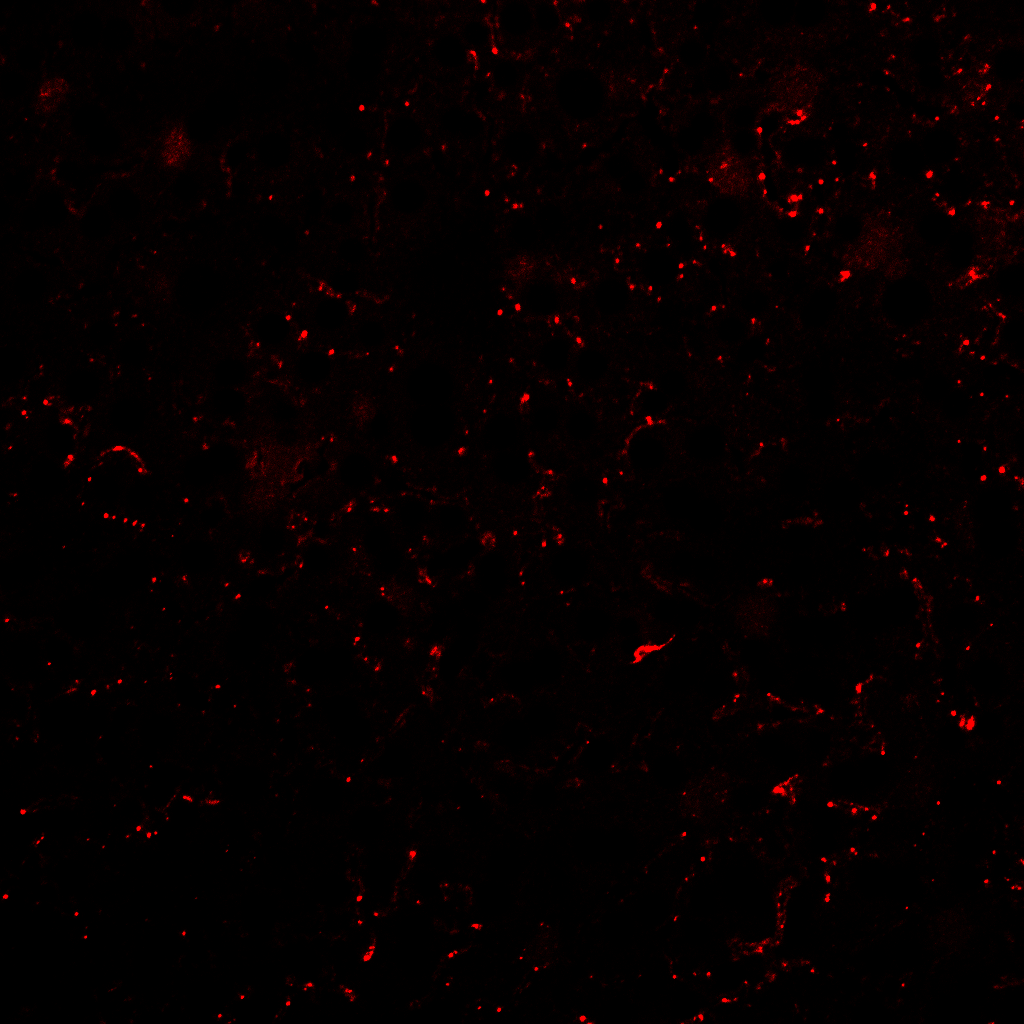

Supplement: Supplementary file 5 — Source Data for Figure 2 [file EMMM-13-e14563-s007.zip › Source Data Figure 2F/Homozygote/Homozygote_Bsep.tif]

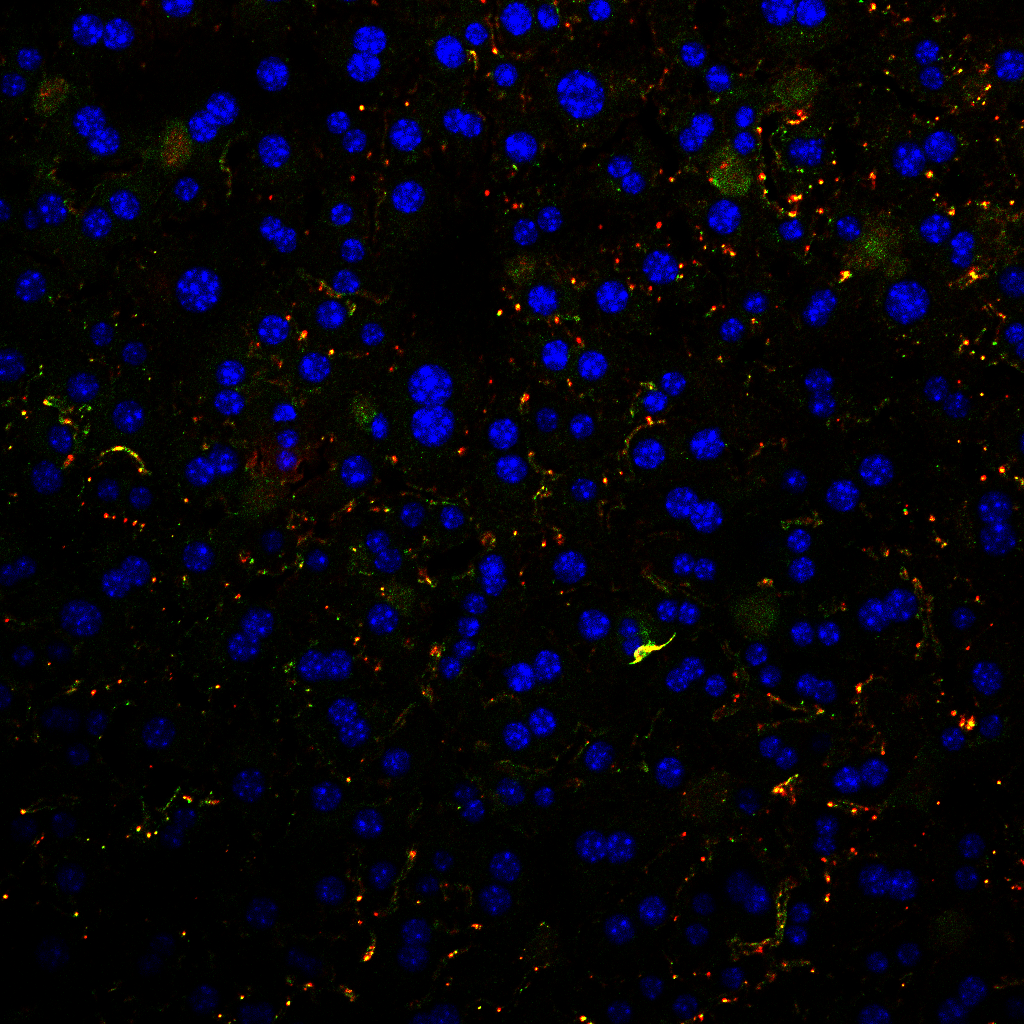

Supplement: Supplementary file 5 — Source Data for Figure 2 [file EMMM-13-e14563-s007.zip › Source Data Figure 2F/Homozygote/Homozygote_Merge.tif]

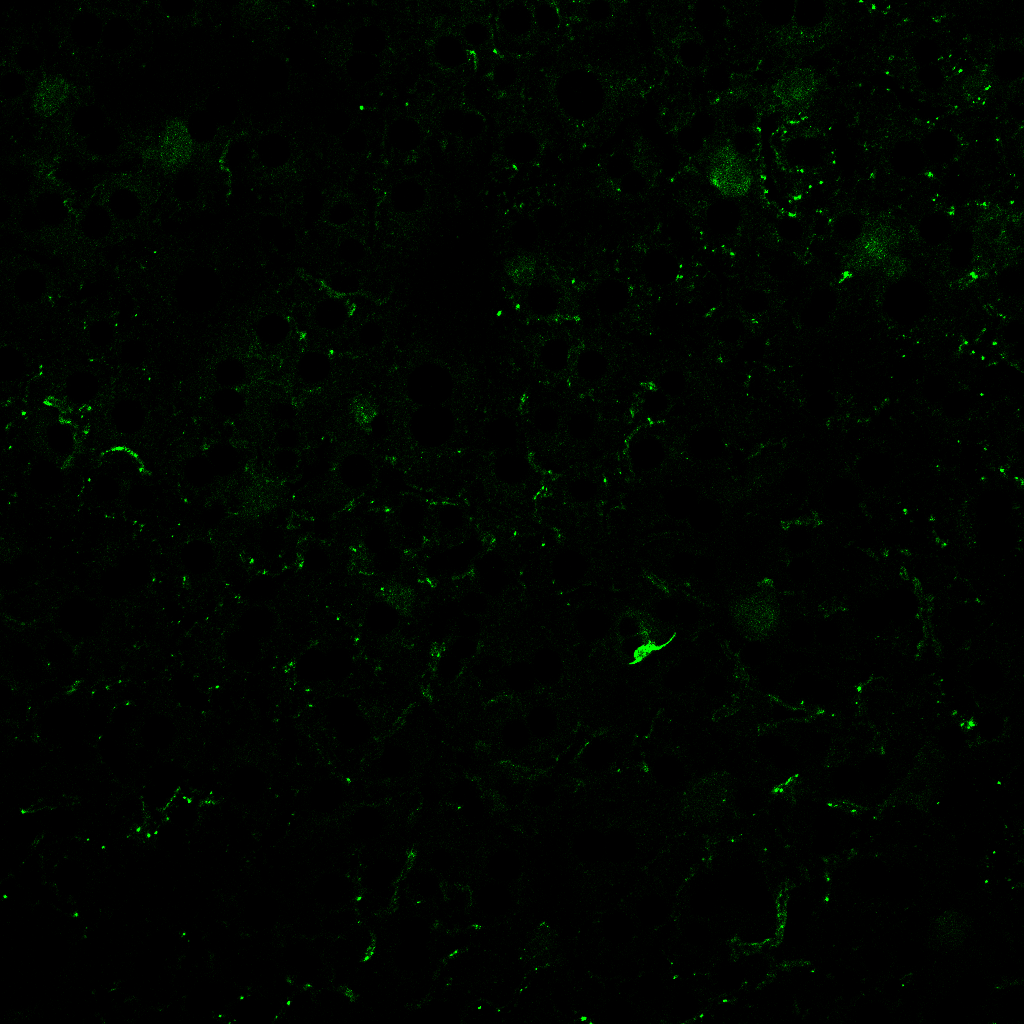

Supplement: Supplementary file 5 — Source Data for Figure 2 [file EMMM-13-e14563-s007.zip › Source Data Figure 2F/Homozygote/Homozygote_Mrp2.tif]

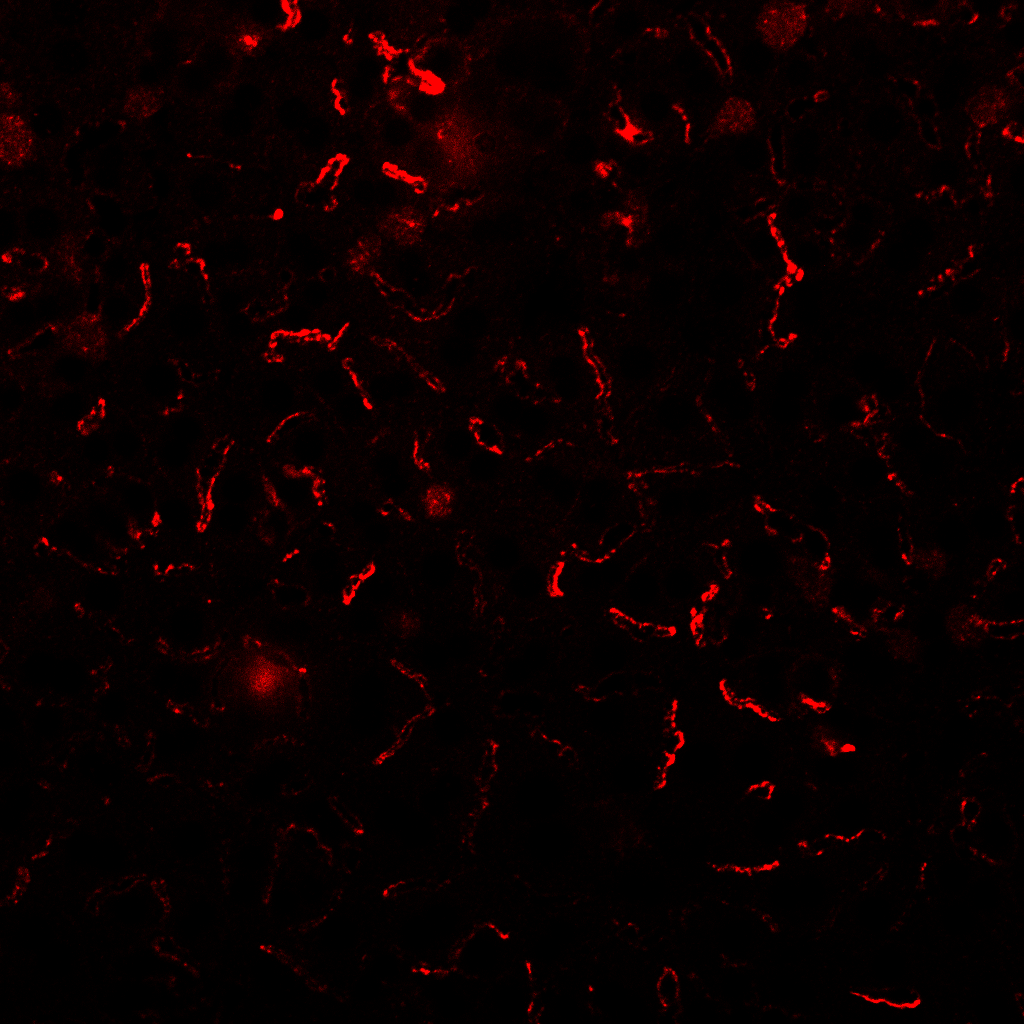

Supplement: Supplementary file 5 — Source Data for Figure 2 [file EMMM-13-e14563-s007.zip › Source Data Figure 2F/Wild type/Wild type_Bsep.tif]

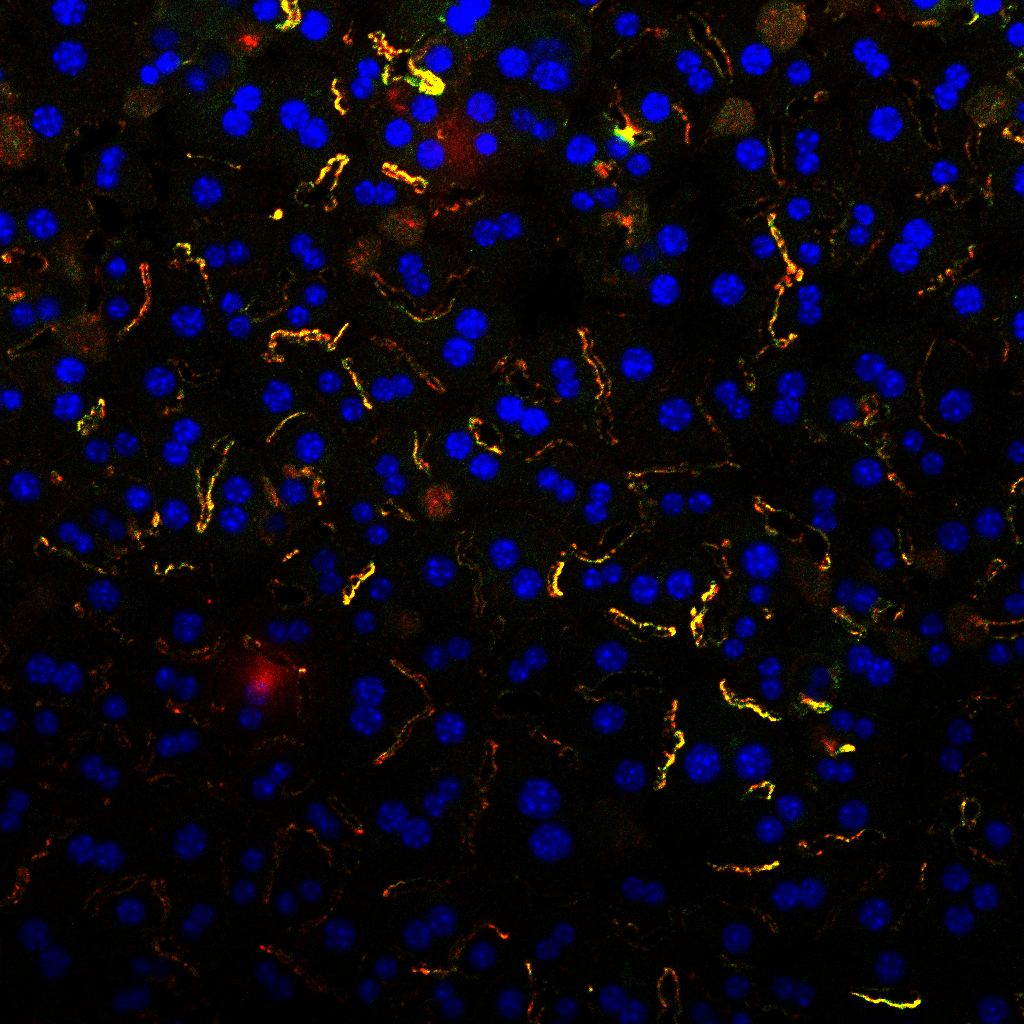

Supplement: Supplementary file 5 — Source Data for Figure 2 [file EMMM-13-e14563-s007.zip › Source Data Figure 2F/Wild type/Wild type_Merge.tif]

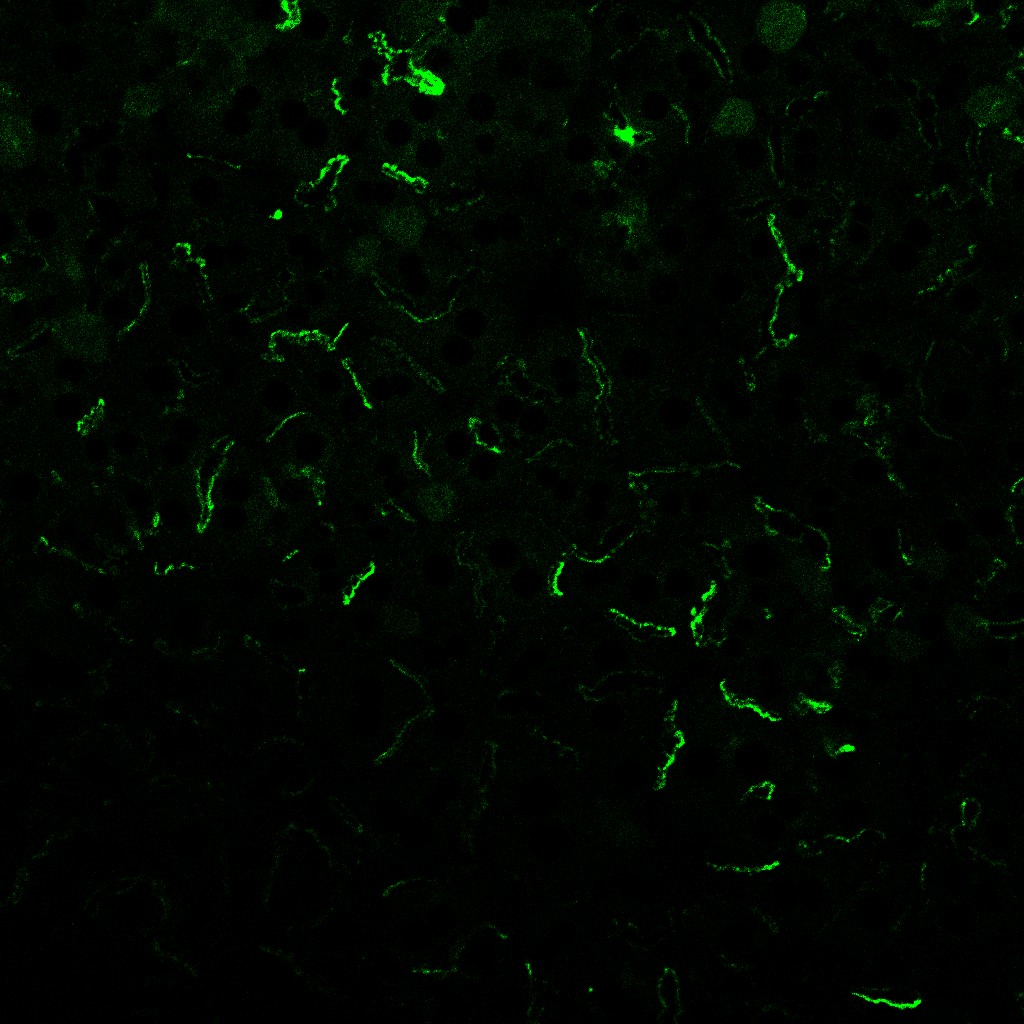

Supplement: Supplementary file 5 — Source Data for Figure 2 [file EMMM-13-e14563-s007.zip › Source Data Figure 2F/Wild type/Wild type_Mrp2.tif]

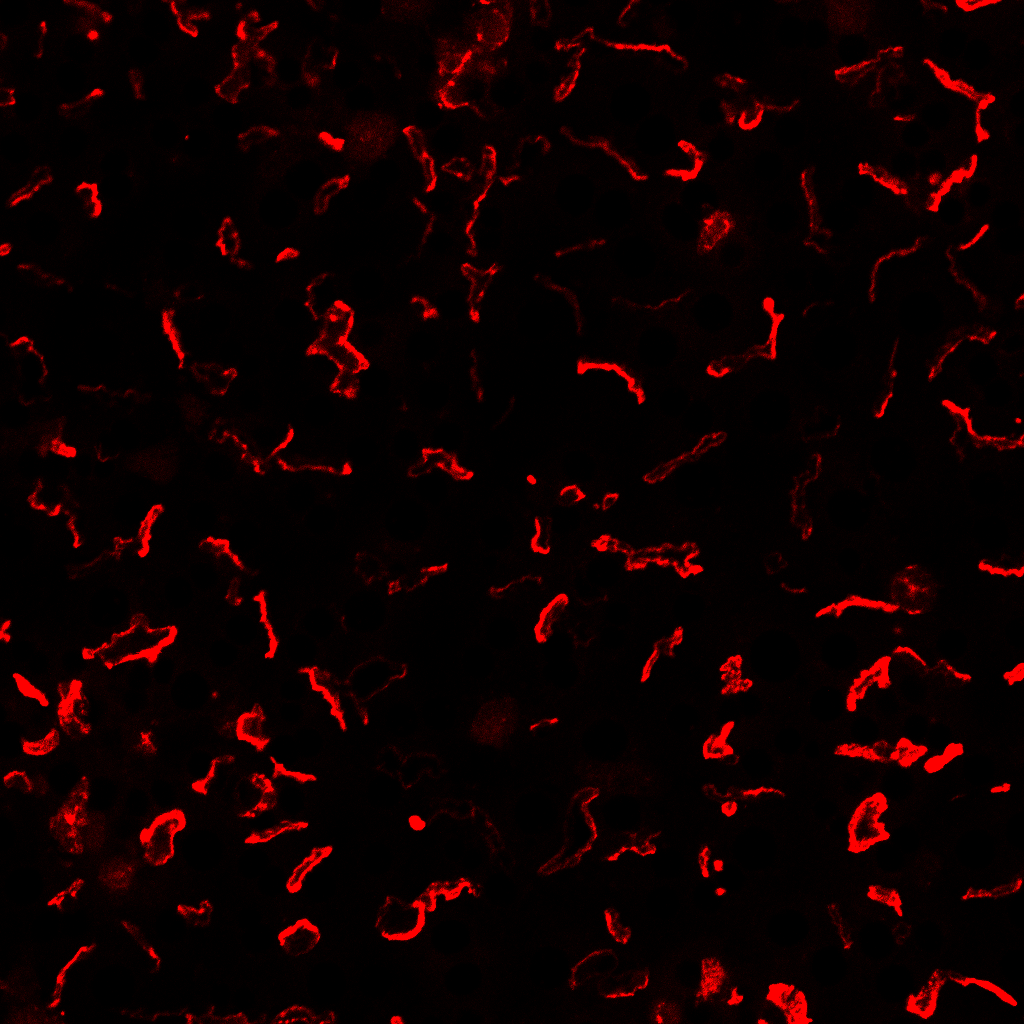

Supplement: Supplementary file 5 — Source Data for Figure 2 [file EMMM-13-e14563-s007.zip › Source Data Figure 2G/CTR/CTR_BSEP.tif]

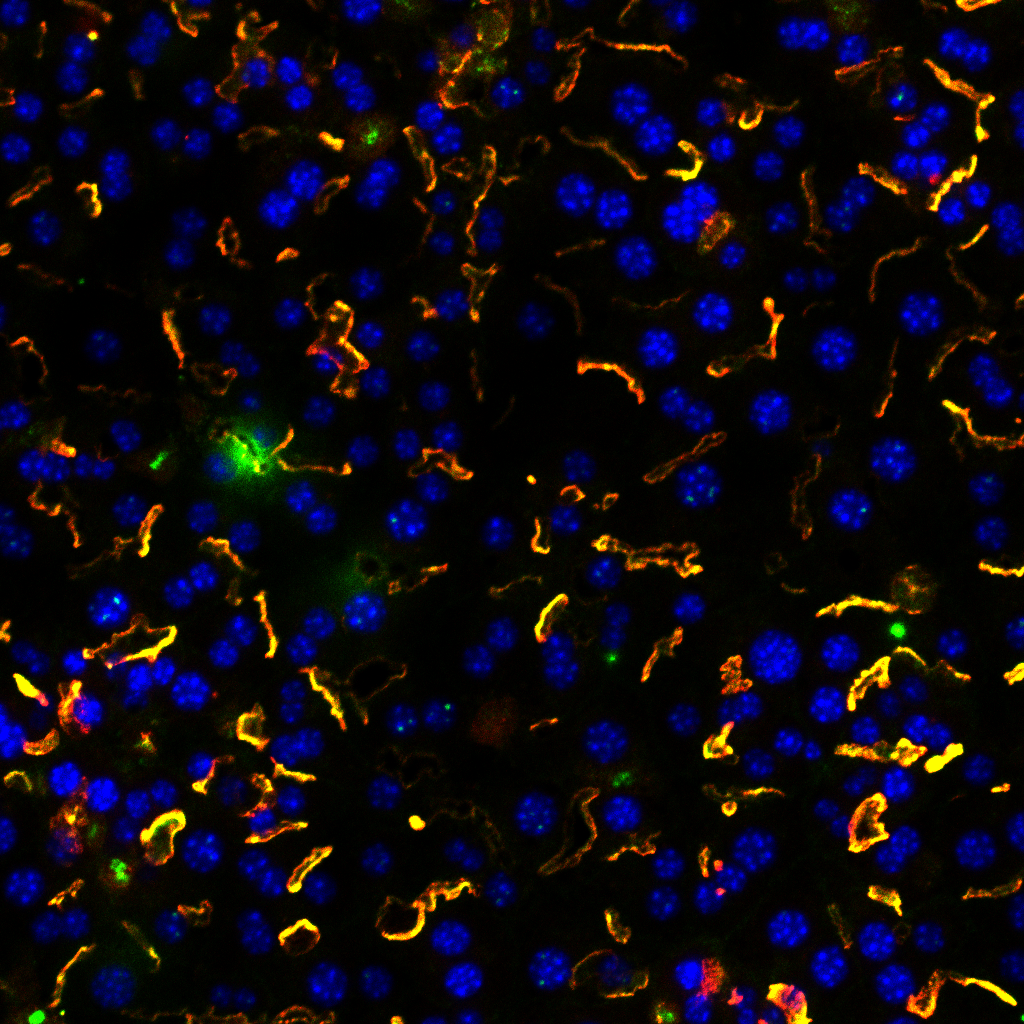

Supplement: Supplementary file 5 — Source Data for Figure 2 [file EMMM-13-e14563-s007.zip › Source Data Figure 2G/CTR/CTR_Merge.tif]

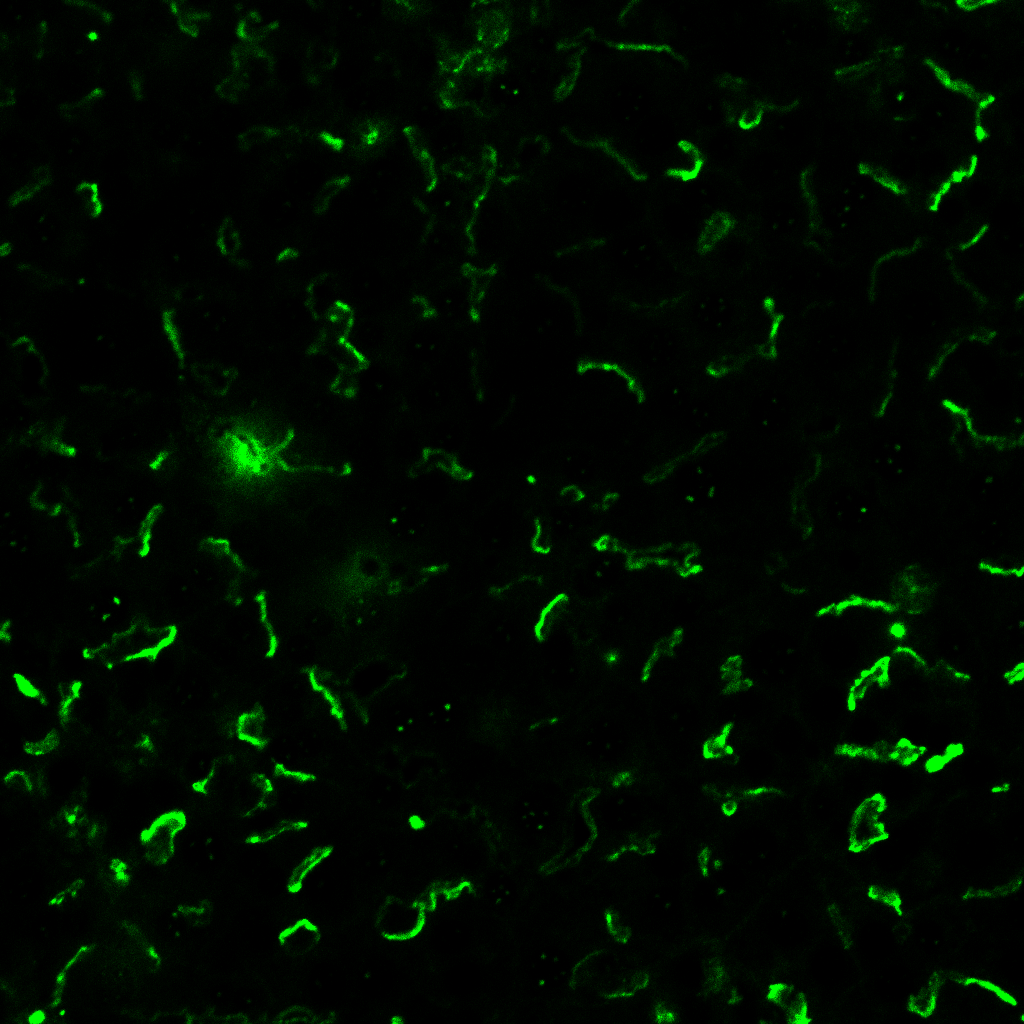

Supplement: Supplementary file 5 — Source Data for Figure 2 [file EMMM-13-e14563-s007.zip › Source Data Figure 2G/CTR/CTR_MRP2.tif]

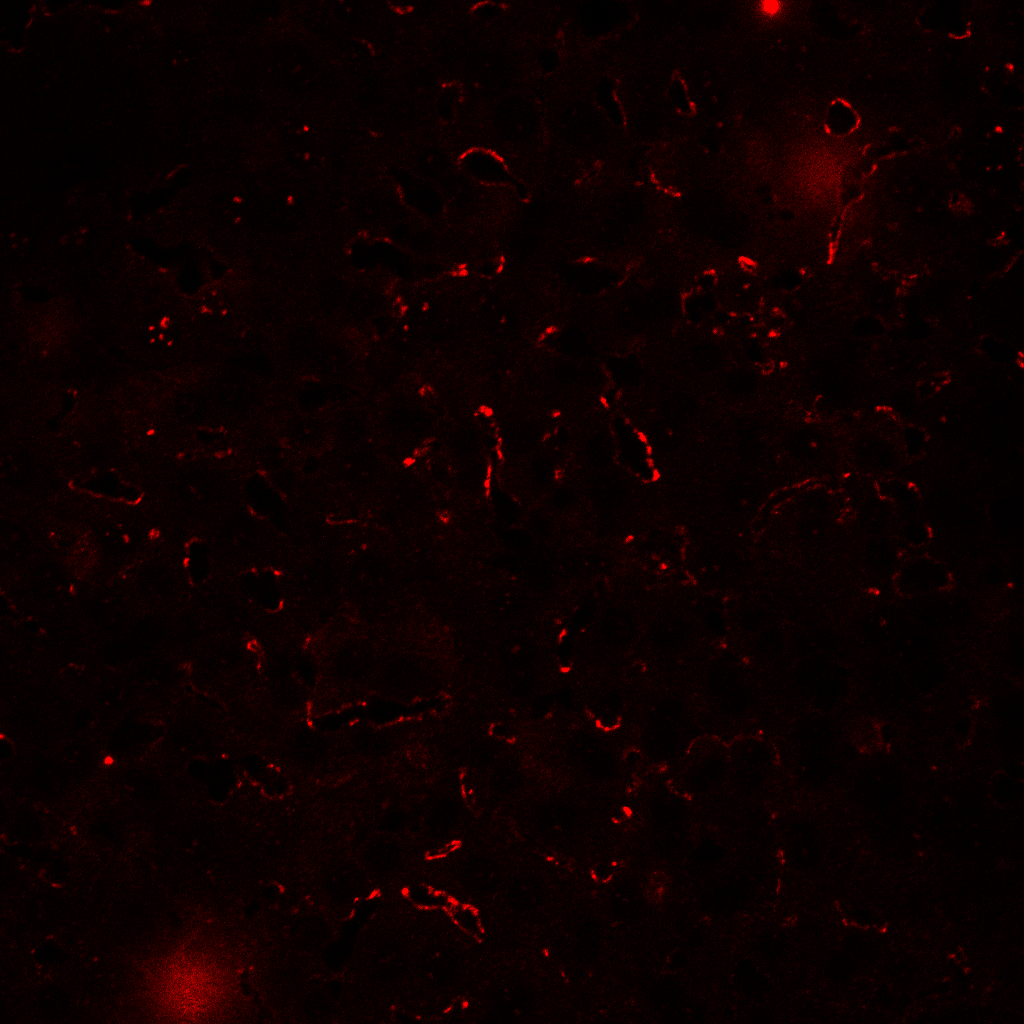

Supplement: Supplementary file 5 — Source Data for Figure 2 [file EMMM-13-e14563-s007.zip › Source Data Figure 2G/SEMA7A_R148W_0.2μg/SEMA7A_R148W_0.2μg_BSEP.tif]

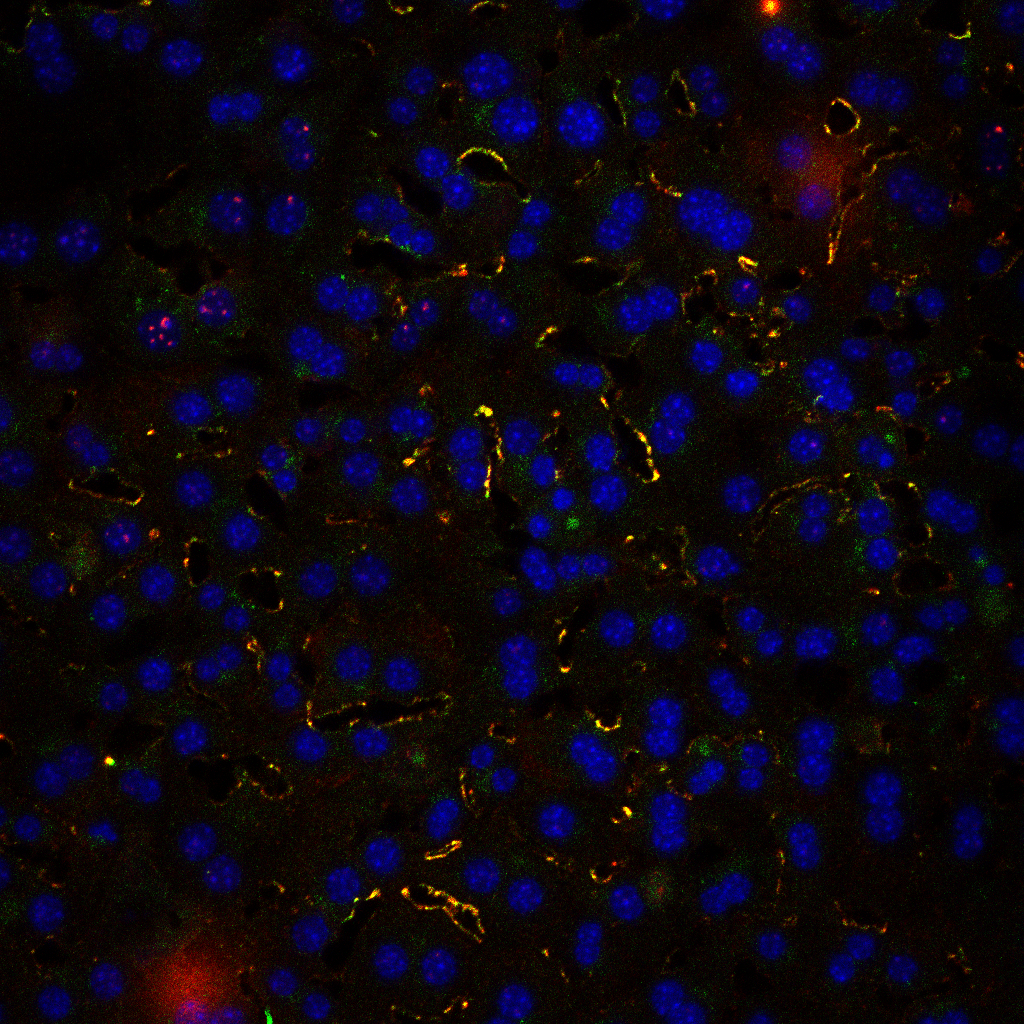

Supplement: Supplementary file 5 — Source Data for Figure 2 [file EMMM-13-e14563-s007.zip › Source Data Figure 2G/SEMA7A_R148W_0.2μg/SEMA7A_R148W_0.2μg_Merge.tif]

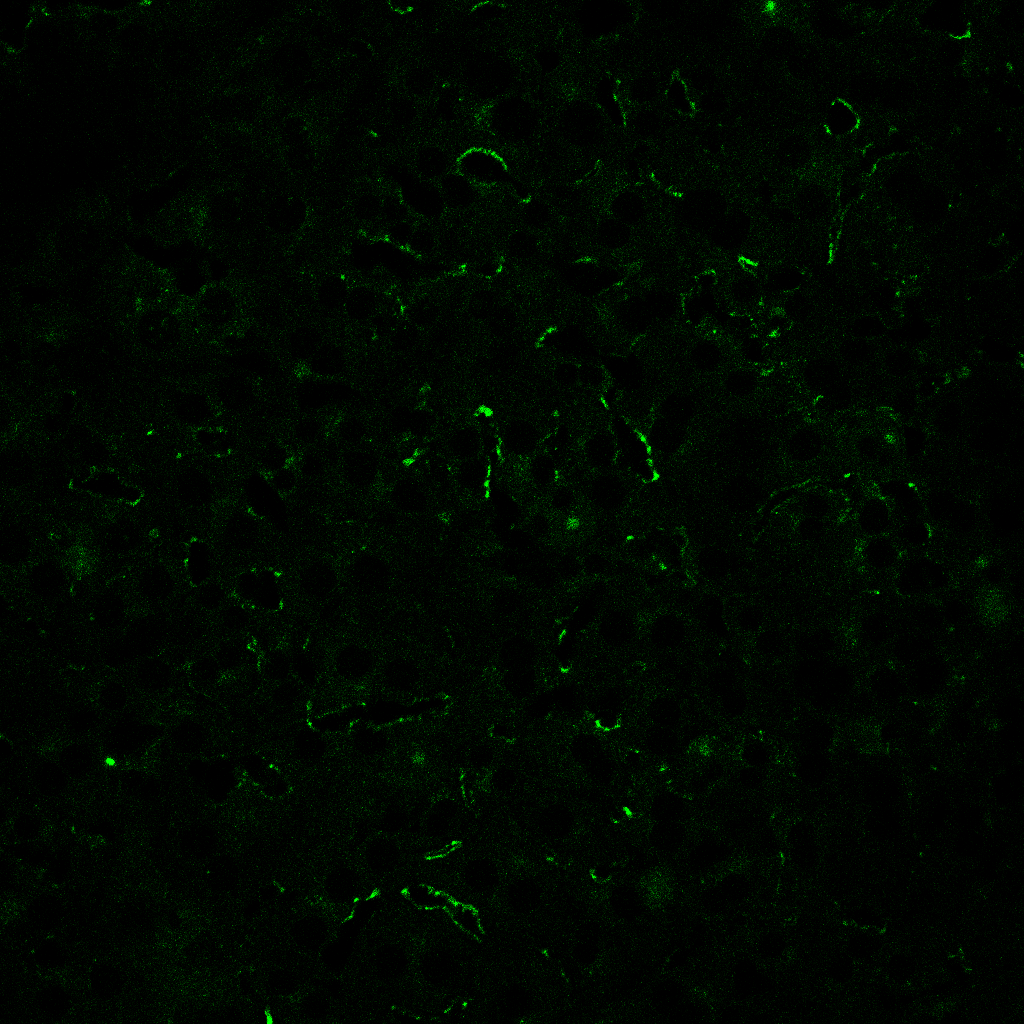

Supplement: Supplementary file 5 — Source Data for Figure 2 [file EMMM-13-e14563-s007.zip › Source Data Figure 2G/SEMA7A_R148W_0.2μg/SEMA7A_R148W_0.2μg_MRP2.tif]

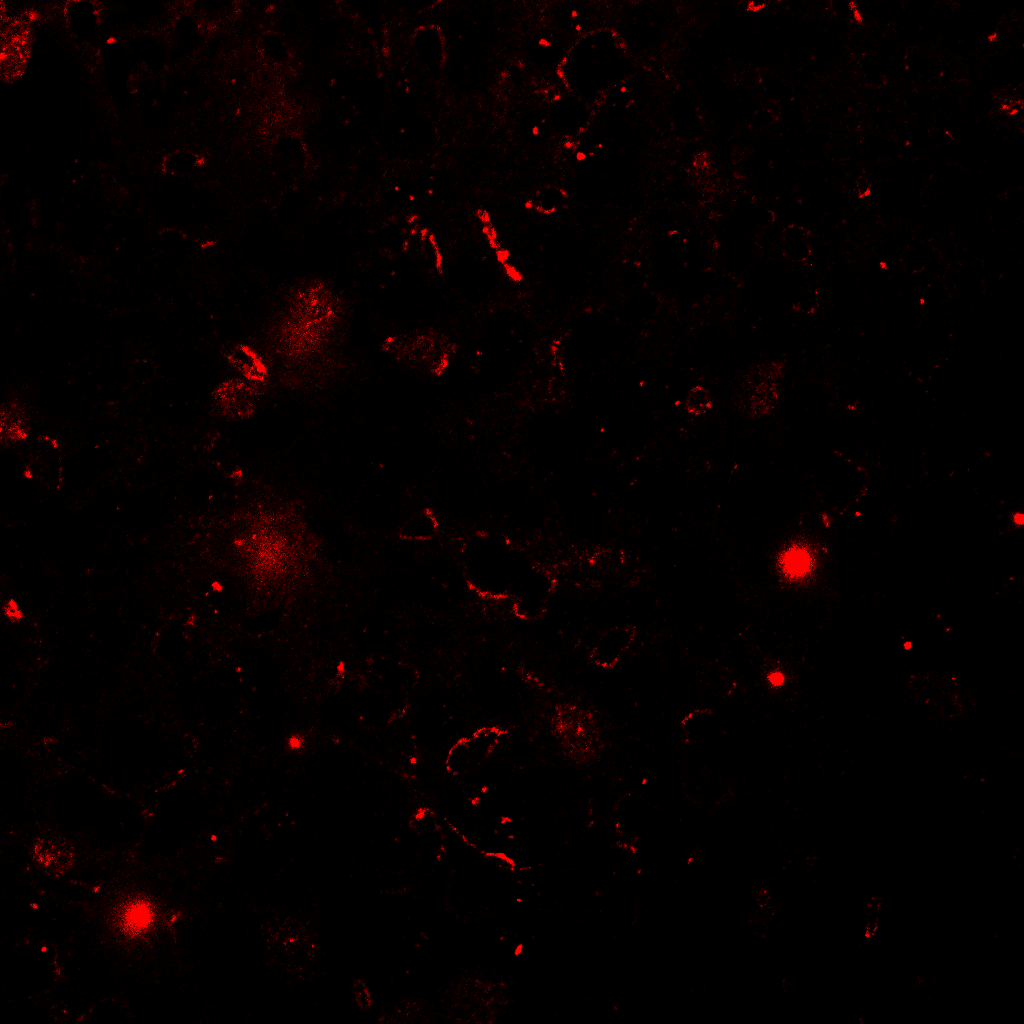

Supplement: Supplementary file 5 — Source Data for Figure 2 [file EMMM-13-e14563-s007.zip › Source Data Figure 2G/SEMA7A_R148W_1.0μg/SEMA7A_R148W_1.0μg_BSEP.tif]

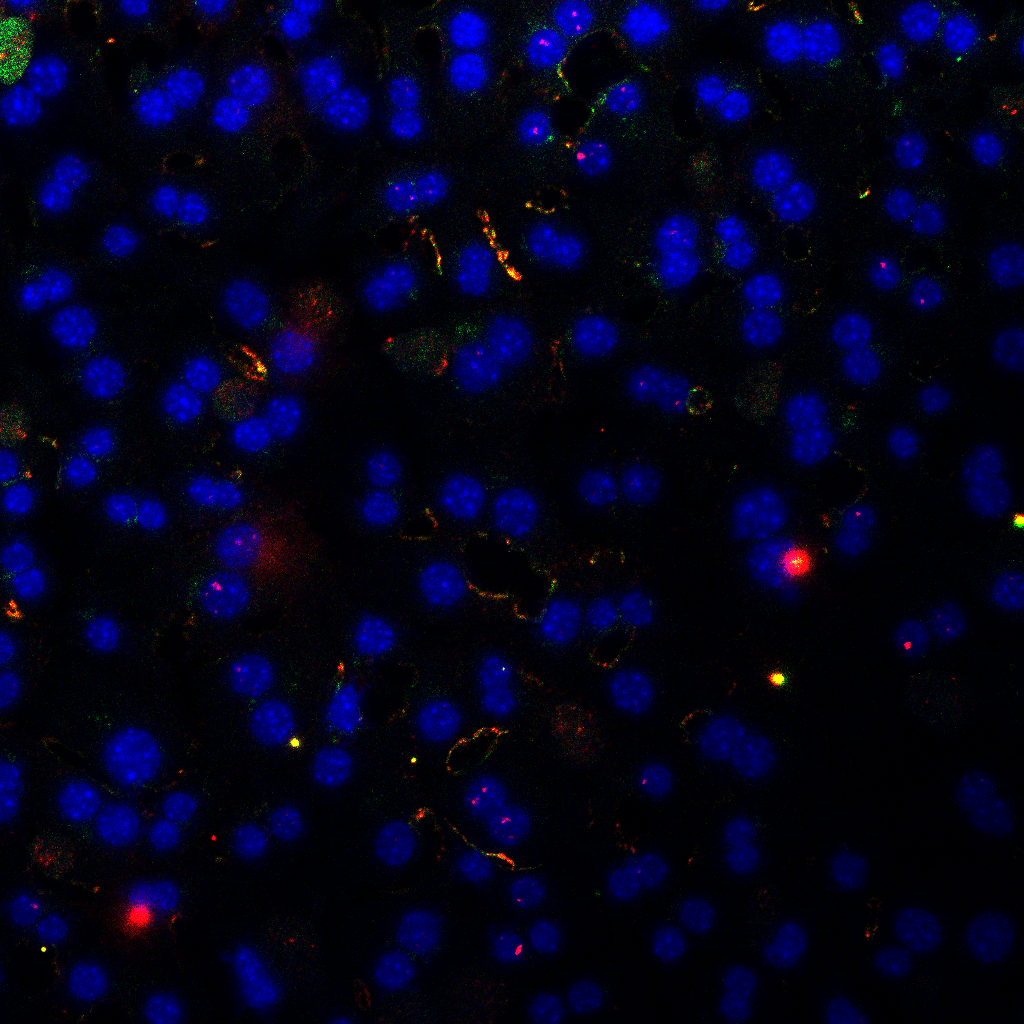

Supplement: Supplementary file 5 — Source Data for Figure 2 [file EMMM-13-e14563-s007.zip › Source Data Figure 2G/SEMA7A_R148W_1.0μg/SEMA7A_R148W_1.0μg_Merge.tif]

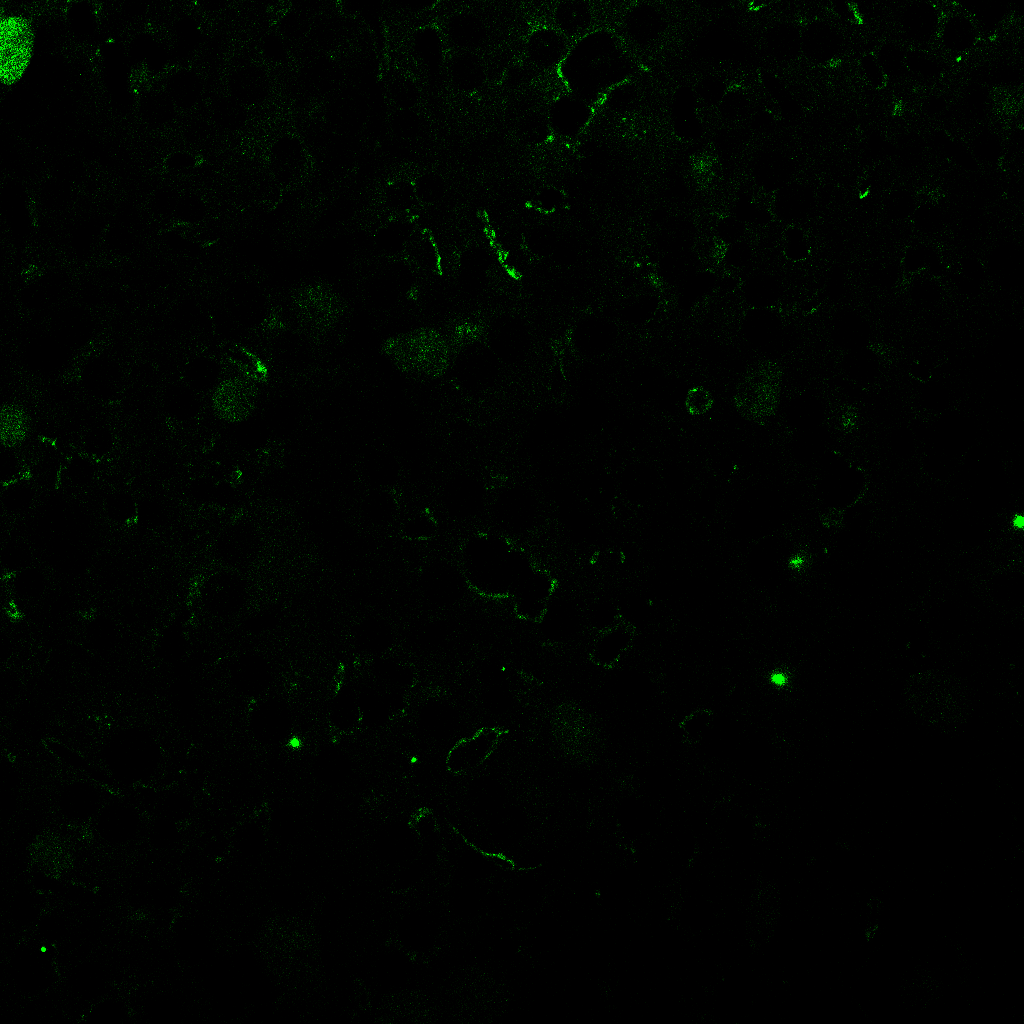

Supplement: Supplementary file 5 — Source Data for Figure 2 [file EMMM-13-e14563-s007.zip › Source Data Figure 2G/SEMA7A_R148W_1.0μg/SEMA7A_R148W_1.0μg_MRP2.tif]

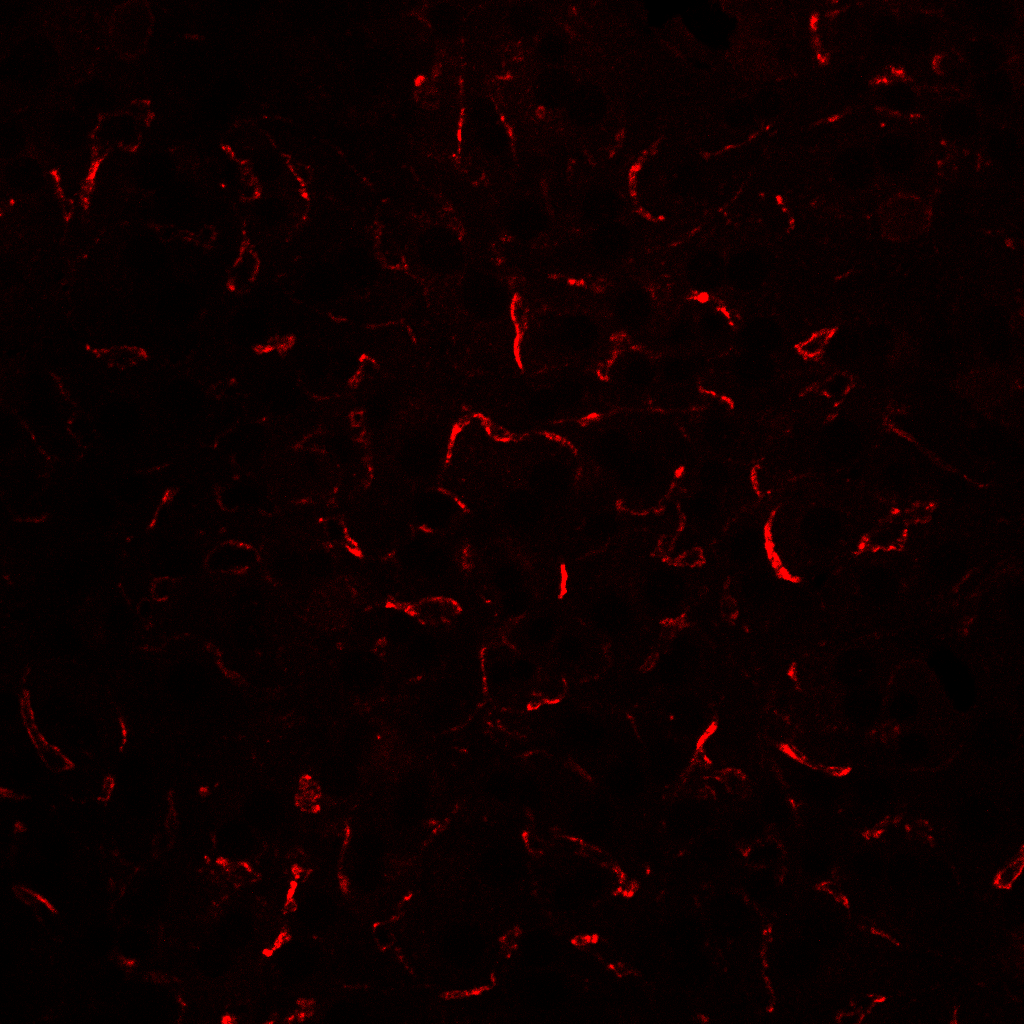

Supplement: Supplementary file 5 — Source Data for Figure 2 [file EMMM-13-e14563-s007.zip › Source Data Figure 2G/SEMA7A_WT_0.2μg/SEMA7A_WT_0.2μg_BSEP.tif]

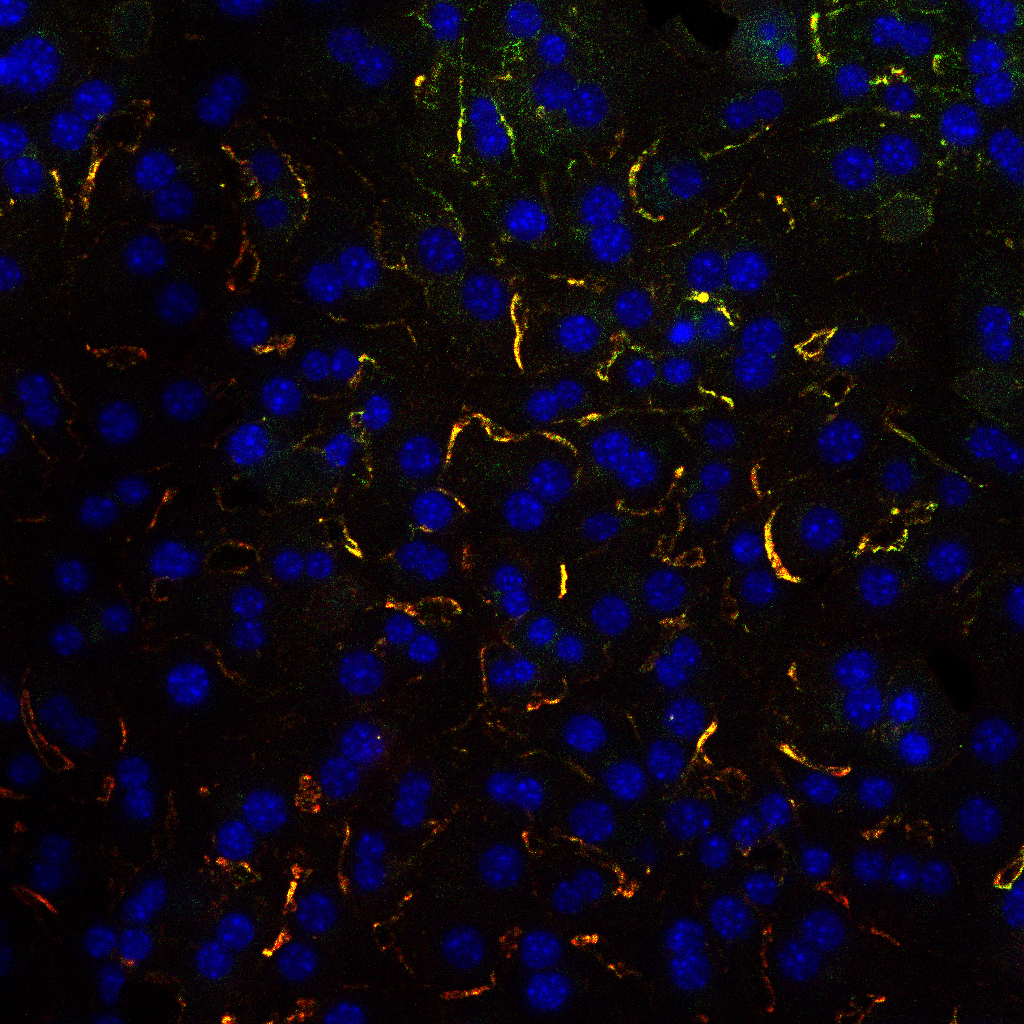

Supplement: Supplementary file 5 — Source Data for Figure 2 [file EMMM-13-e14563-s007.zip › Source Data Figure 2G/SEMA7A_WT_0.2μg/SEMA7A_WT_0.2μg_Merge.tif]

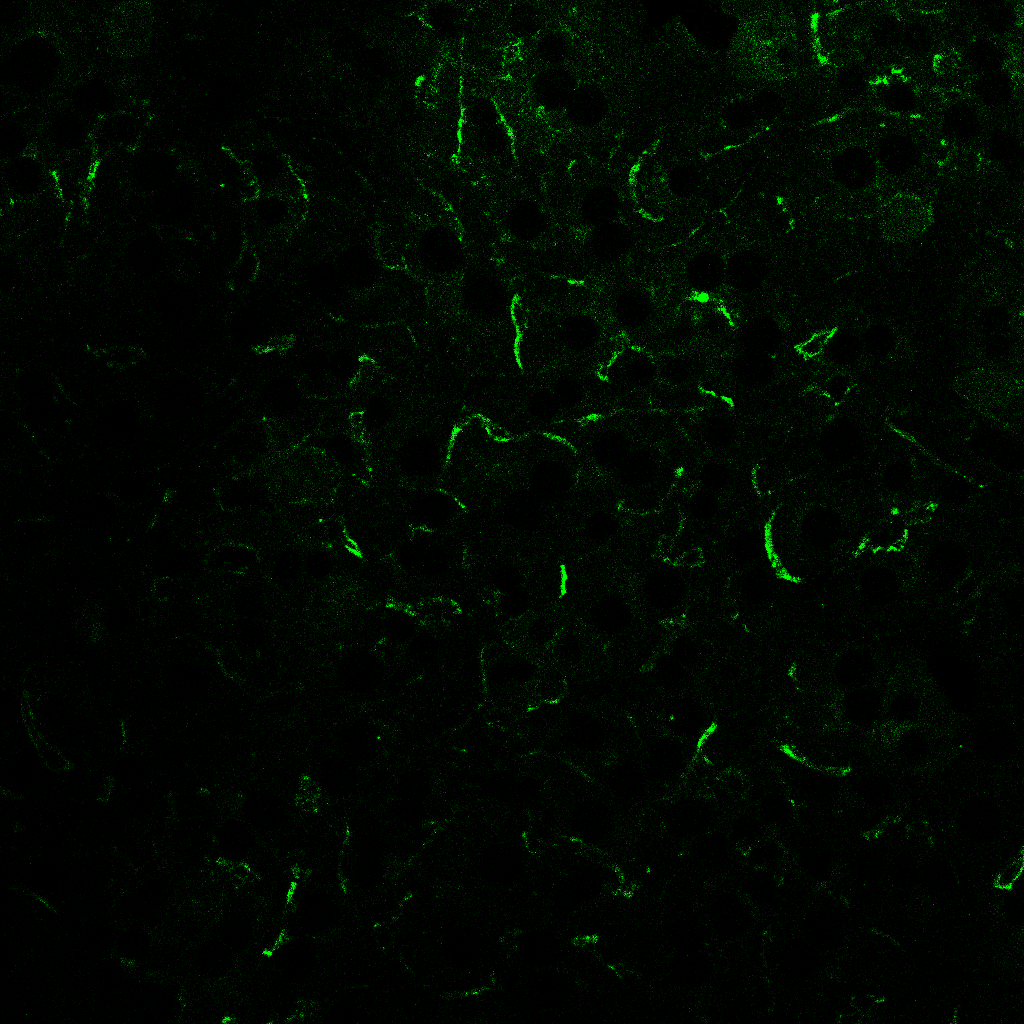

Supplement: Supplementary file 5 — Source Data for Figure 2 [file EMMM-13-e14563-s007.zip › Source Data Figure 2G/SEMA7A_WT_0.2μg/SEMA7A_WT_0.2μg_MRP2.tif]

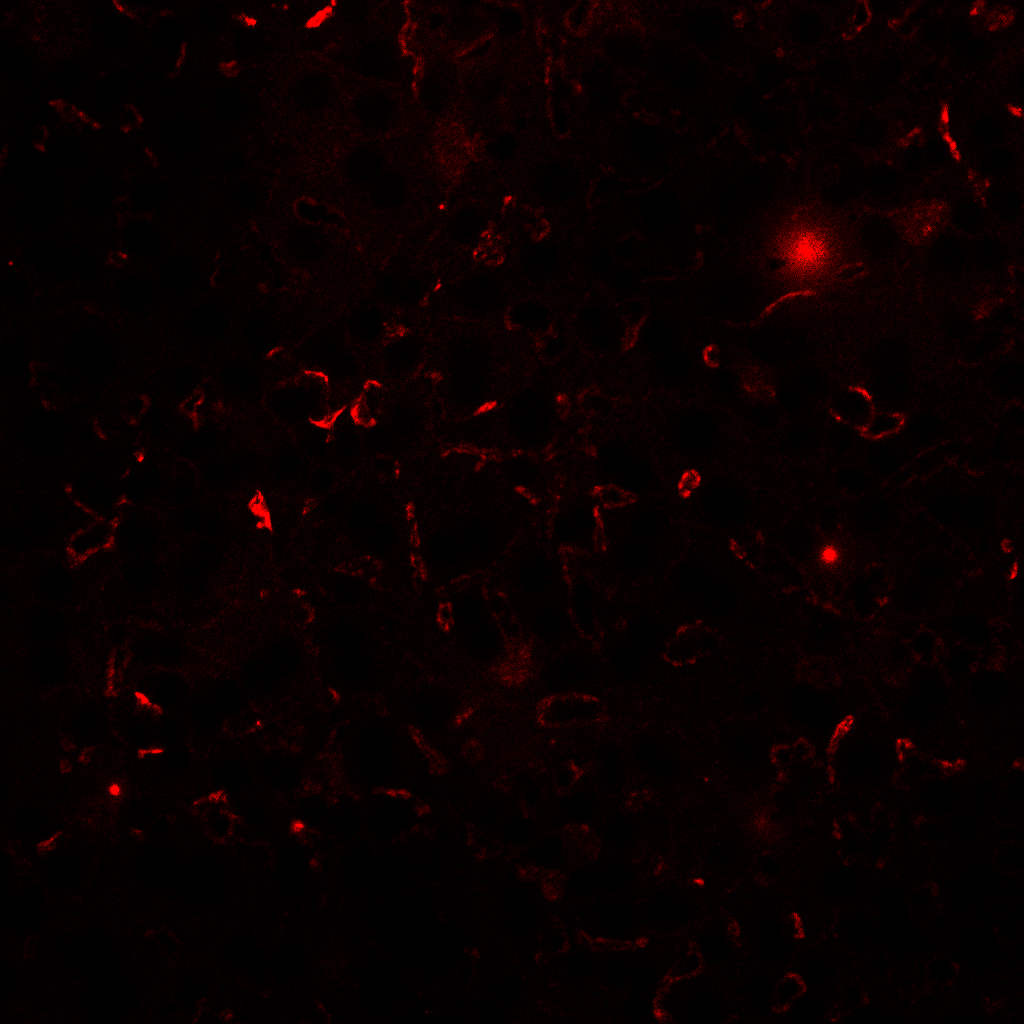

Supplement: Supplementary file 5 — Source Data for Figure 2 [file EMMM-13-e14563-s007.zip › Source Data Figure 2G/SEMA7A_WT_1.0μg/SEMA7A_WT_1.0μg_BSEP.tif]

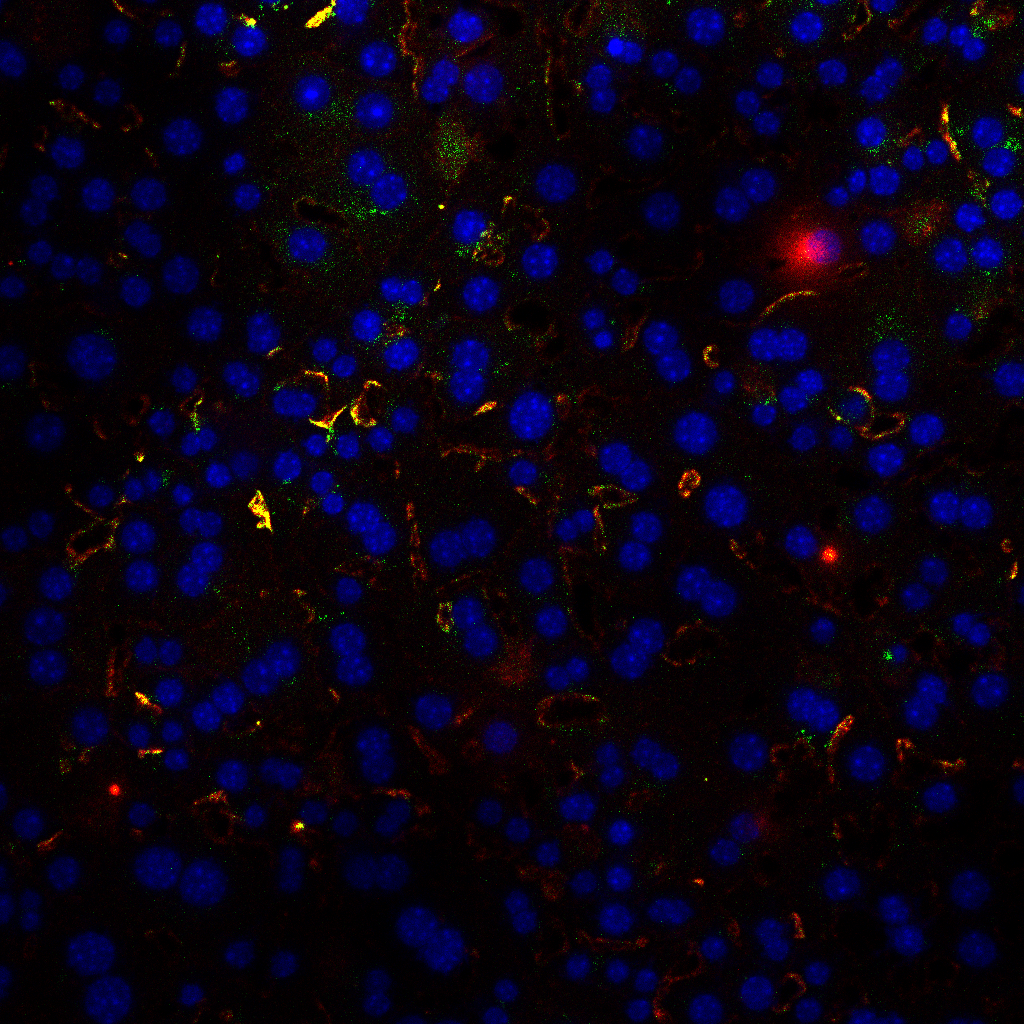

Supplement: Supplementary file 5 — Source Data for Figure 2 [file EMMM-13-e14563-s007.zip › Source Data Figure 2G/SEMA7A_WT_1.0μg/SEMA7A_WT_1.0μg_Merge.tif]

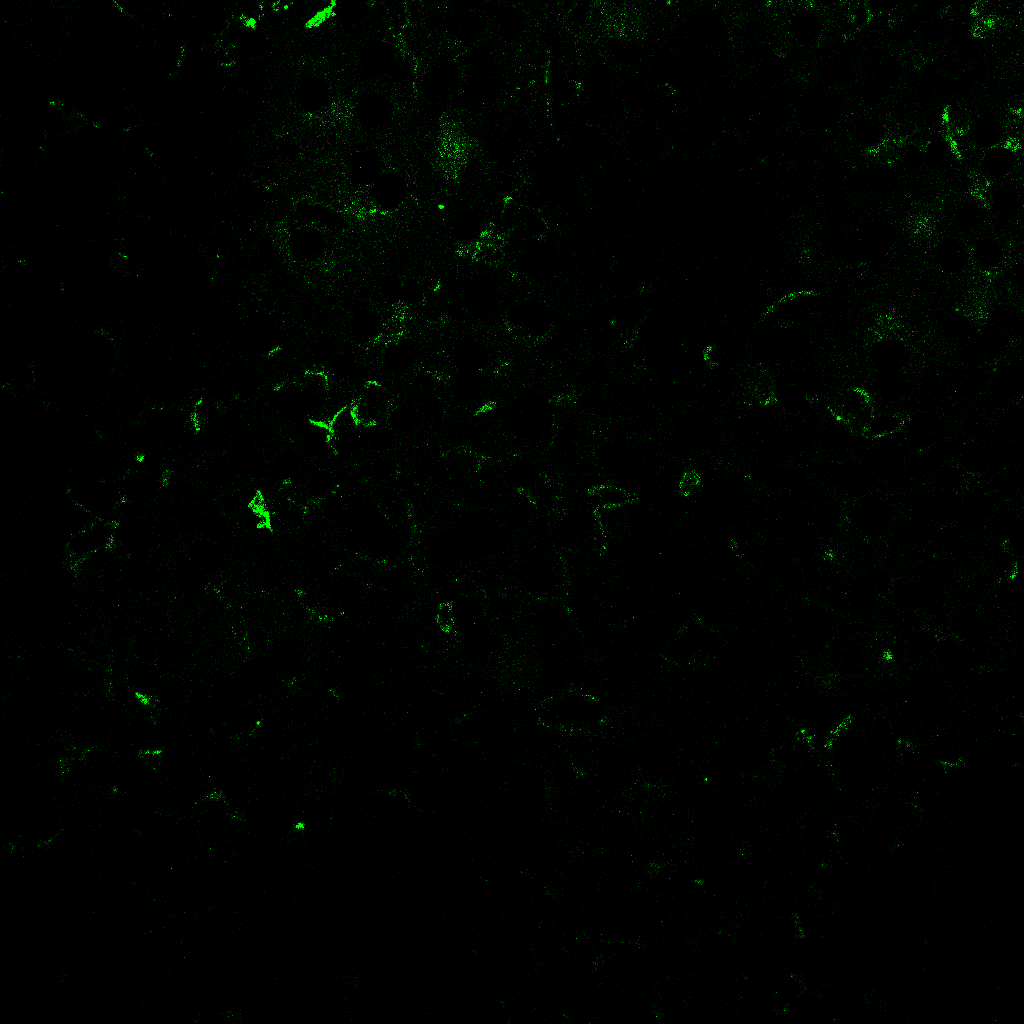

Supplement: Supplementary file 5 — Source Data for Figure 2 [file EMMM-13-e14563-s007.zip › Source Data Figure 2G/SEMA7A_WT_1.0μg/SEMA7A_WT_1.0μg_MRP2.tif]
