# Supplementary material for: A homozygous R148W mutation in Semaphorin 7A causes progressive familial intrahepatic cholestasis
Source: EMBO Mol Med. 2021 Sep 29;13(11):e14563. doi: 10.15252/emmm.202114563 (PMC8573601; doi:10.15252/emmm.202114563)
Supplement: Supplementary file 7 [file EMMM-13-e14563-s001.pdf]

EMBO Molecular Medicine

A homozygous R148W mutation in *Semaphorin 7A* causes progressive familial intrahepatic cholestasis

Qiong Pan, Gang Luo, Jiaquan Qu, Sheng Chen, Xiaoxun Zhang, Nan Zhao, Jingjing Ding, Hong Yang, Mingqiao Li, Ling Li, Ying Cheng, Xuan Li, Qiaoling Xie, Qiao Li, Xueqian Zhou, Huiling Zou, Shijun Fan, Lingyun Zou, Wei Liu, Guohong Deng, Shi-Ying Cai, James L Boyer, and Jin Chai
DOI: 10.15252/emmm.202114563

Corresponding author(s): Jin Chai (jin.chai@cldcsw.org)

Review Timeline:

Submission Date:	17th May 21
Editorial Decision:	2nd Jun 21
Revision Received:	17th Aug 21
Editorial Decision:	30th Aug 21
Revision Received:	3rd Sep 21
Accepted:	7th Sep 21

Editor: Zeljko Durdevic

Transaction Report:

(Note: With the exception of the correction of typographical or spelling errors that could be a source of ambiguity, letters and reports are not edited. Depending on transfer agreements, referee reports obtained elsewhere may or may not be included in this compilation. Referee reports are anonymous unless the Referee chooses to sign their reports.)

1st Editorial Decision 2nd Jun 2021

2nd Jun 2021

Dear Prof. Chai,

Thank you for the submission of your manuscript to EMBO Molecular Medicine. We have now received feedback from the three reviewers who agreed to evaluate your manuscript. As you will see from the reports below, the referees acknowledge the interest of the study but also raise serious and partially overlapping concerns that should be addressed in a major revision. Furthermore, I would like you to consider publishing your manuscript as a scientific report (3 figures, ~22000 characters), for more information please check our "Author Guidelines". https://www.embopress.org/page/journal/17574684/authorguide#reportsarticleguide.

Further consideration of a revision that addresses reviewers' concerns in full will entail a second round of review. EMBO Molecular Medicine encourages a single round of revision only and therefore, acceptance or rejection of the manuscript will depend on the completeness of your responses included in the next, final version of the manuscript. For this reason, and to save you from any frustrations in the end, I would strongly advise against returning an incomplete revision.

We would welcome the submission of a revised version within three months for further consideration. However, we realize that the current situation is exceptional on the account of the COVID-19/SARS-CoV-2 pandemic. Please let us know if you require longer to complete the revision.

I look forward to receiving your revised manuscript.

Yours sincerely,

Zeljko Durdevic

***** Reviewer's comments *****

Referee #1 (Comments on Novelty/Model System for Author):

Some differences between patient and animal model.
Conclusions on BSEP/MRP2 trafficking that would require cellular models as 'internalization'is a dynamic process, while only static data is provided.

Impact is unclear as no data is provided how often Sema7A mutations could lead to PFIC. This is in itself already a rare disease, so this issue is difficult to answer and not required for a first paper identifying a novel disease-causing gene variant I think. Highly novel and important though

The molecular explanation for cholestasis is somewhat preliminary and if not bettter addressed, I would recommend this paper for a short report instead.

Referee #1 (Remarks for Author):

The present study describes a genome-wide mutational analysis in a single patient with PFIC like cholestasis. They identified Semaphorin 7A as a putative causal gene, confirm a causal relation with respect to liver damage using KO mice and investigate the underlying mechanism by studying the role of Semaphorin on trafficking of 2 pivotal bile salt excretion proteins; BSEP and MRP2. The identification of Semaphorin 7A as a novel PFIC gene is novel and fascinating, and opens multiple avenues for future studies with possible therapeutic implications.

Major comments.
The molecular explanation linking Sema7a dysfunction to BSEP/MDSR2 trafficking is not very convincing. Data is not very clear, with much background signal and also effects in heterozygous animals. Importantly, no data on protein internalization is provided at all, while this conclusion is stated in both the abstract and discussion. No rationale that links sema7a to canalicular proteins is provided.

The paper seems to contradict earlier observations that Sema7A deficiency is protective against liver injury in BDL and CCL4 induced liver damage. The authors explain this apparent discrepancy by stating that the R145W variant may be a gain of function variant. This hypothesis should be further explored using a Sema7A dosing in BSEP expressing cell models and seems not to match well with a recessive mode of inheritance.

Minor comments
The liver phenotype of the Sema7a_R145W mice is different from the patients with respect to bilirubin levels. This is somewhat remarkable as hyperbilirubinemia is described for Slc10a1 deficiency (which is present in the patient). Are similar differences due to species differences and also observed with other PFIC genes?

The PFIC phenotype is different from PFIC1/2 in the sense that pruritus was absent. This should be discussed.

Recent data that Sema7A is crucial for resolution of severe inflammation (PNAS 2021; PMID: 33637648) should be discussed as inflammation plays a role in cholestatic liver injury..

Referee #2 (Comments on Novelty/Model System for Author):

In general the study is of interest but lack a mechanistic explanation ofm the findings.

Referee #2 (Remarks for Author):

This study reports on the characterization of a Semaphorin 7a mutation in a female infant showing features of liver injury and cholestasis at 2 months of age. The clinical syndrome is marked by increased levels of AST and ALT and total bile acids (TBA) in the blood. Both the father and the mother had normal AST and ALT and TBA. Liver histopathology also revealed a robust hydropic degeneration in hepatocytes and increased accumulation of conjugated BA in the liver, while immunoshistochemistry in mice demonstrated an internalization of canalicular membrane BSEP and MRP2 .
Gene sequencing revealed a mutation in the semaphoring 7a gene. The newly identified mutation is a homozygous p.R148W mutation in SEMA7A, and SEMA7A R145W homozygous mice, displayed similar histopathology features than the patients, including a hydropic degeneration of hepatocytes and increased accumulation of intrahepatic BA.
The data suggest that SEMA7AR148W homozygous mutation is a new genetic determinant of cholestatic liver injury.
The data reported are of interest.
My comments are the detailed here:
1. The first sentence of Discussion is an over-interpretation of the results: "Here, we first reported a case of familial cholestasis caused by a homozygous p.R148W mutation in SEMA7A, and provided evidence of its pathophysiologic mechanism. This new genetic cholestatic disorder is characterized by (1) elevated levels of serum ALT, AST, and TBA (Fig.2A-C and Tables.3&S13); (2) striking hydropic degeneration in hepatocytes and increased accumulation of conjugated BA in the liver (Fig.2D&E and Table.4); and (3) internalization of canalicular membrane BSEP and MRP2 (Figs.3D&E". The authors have no data on the histopathology of the patient described in this report, therefore there is no information on point 2 and 3. This need to be amended and the same should be done in the abstract and throughout the manuscript.
2. Also the fact that the patient has a homozygous p.S267F mutation in SLC10A1, which leads to very close clinical pattern, does not allow a clear separation between the two syndromes. This suggest a cautionary approach on the title and abstract. The abstract should inform the readers that the patients had both mutations.

3. Page 16, the human mutation SEMA7AR148W is described as a "gain of function" mutation. The patient harboring this mutation had cholestasis and therefore the mutation should lead to some degree of loss of function. Particularly if the SEMA7AR148W prevents insertion of BSEP and MRP2 in the canalicular membrane of hepatocytes. Please clarify.
4. A previous study has shown that lack of semaforin7a is protective again cholestasis in mice, which is in contrast with the data shown in this paper. Refence made to this paper in page 15 is wrong since the paper is cited in ref. 7 (not 6). Furthermore, this paper has been criticized because extensive image duplications: https://pubpeer.com/publications/089AD44F94ECA4B33A4BF1718F644E. I will suggest to remove it from reference.
5. One limitation of the study is the lack of a mechanism explaining how SEM7A regulates BSEP insertion at the canalicular membrane. The structure shown in Figure 1D-F is poorly informative.
6. Table 4, shows a disproportionate increase of TCDCA over TMCA, suggesting an impaired conversion of CDCA to MCA. Does the authors have data on Cyp2c70?
7. How the authors explain differential regulation of Ost and Mrp4 in figure 2

Referee #3 (Comments on Novelty/Model System for Author):

We constantly learn about novel mutations. Many of these are variants of unknown significance. The locus the authors describe is interesting but they cannot convincingly correlate the in vitro and in silico findings to a human clinical phenotype.

Referee #3 (Remarks for Author):

This is potentially an interesting finding however I see several drawbacks that should be addressed before publication:
1. There is no convincing correlation of the in vitro and in silico findings to the clinical phenotype in the child. Histopathology and longitudinal clinical data is missing in particular as previous publication on this gene suggested alleviation of cholestasis rather than a PFIC phenotype.
2. Suggest you follow the reporting guidelines with (equator network)
3. The language needs revision.

Responses to the Editor:

As you will see from the reports below, the referees acknowledge the interest of the study but also raise serious and partially overlapping concerns that should be addressed in a major revision. Furthermore, I would like you to consider publishing your manuscript as a scientific report (3 figures, ~22000 characters), for more information please check our "Author Guidelines".

Response: Thank you very much for the suggestion, we now have revised the manuscript and submit it as a scientific report.

Responses to the Reviewer 1

Referee #1 (Comments on Novelty/Model System for Author):

Some differences between patient and animal model.

Conclusions on BSEP/MRP2 trafficking that would require cellular models as 'internalization' is a dynamic process, while only static data is provided.

Impact is unclear as no data is provided how often Sema7A mutations could lead to PFIC. This is in itself already a rare disease, so this issue is difficult to answer and not required for a first paper identifying a novel disease-causing gene variant I think. Highly novel and important though

The molecular explanation for cholestasis is somewhat preliminary and if not better addressed, I would recommend this paper for a short report instead.

Response: Thank you for your positive comments and constructive suggestions. Now, the manuscript has been revised as a short report.

Referee #1 (Remarks for Author):

The present study describes a genome-wide mutational analysis in a single patient with PFIC like cholestasis. They identified Semaphorin 7A as a putative causal gene, confirm a causal relation with respect to liver damage using KO mice and investigate the underlying mechanism by studying the role of Semaphorin on trafficking of 2 pivotal bile salt excretion proteins; BSEP and MRP2. The identification

of Semaphorin 7A as a novel PFIC gene is novel and fascinating, and opens multiple avenues for future studies with possible therapeutic implications.

Response: We greatly appreciate your positive review of our manuscript.

Major comments.

1. The molecular explanation linking Sema7a dysfunction to BSEP/MDSR2 trafficking is not very convincing. Data is not very clear, with much background signal and also effects in heterozygous animals. Importantly, no data on protein internalization is provided at all, while this conclusion is stated in both the abstract and discussion. No rationale that links sema7a to canalicular proteins is provided.

Response: We agree with your professional comment that no direct evidence demonstrates Bsep and Mrp2 "protein internalization". However, we observed that reduced expression of canalicular membrane Bsep and Mrp2 in *Sema7a*^{R145W} homozygous mouse livers. Therefore, we have modified the phrase "internalization of canalicular membrane Bsep and Mrp2" to "reduced the expression of canalicular membrane Bsep and Mrp2" in the Abstract, Results, and Discussion sections of the revised manuscript. We also performed additional experiments to examine the role of Sema7a in Bsep and Mrp2 expression. Specifically, (1) Multiplex IF analysis in liver sections of WT and *Sema7a*^{R145W} homozygous mice (Fig.2E), as well as Western-blotting analysis (Fig.2D), clearly indicated that *Sema7a*^{R145W} homozygous mutation reduced the expression of canalicular Bsep and Mrp2 in mouse livers. Because of the background signal and effects in heterozygous mice, we replaced the low resolution of IHC images (former Fig.3E) with the high solution of multiplex IF images (Fig.2E). (2) *Sema7a*^{R145W} homozygous mutation also reduced canalicular membrane Bsep and Mrp2 expression in mouse primary hepatocytes in sandwich cultures (Fig.2F). (3) Over-expression of *SEMA7A_R148W* protein markedly reduced the levels of Bsep and Mrp2 on canalicular membrane in a dose dependent manner in primary mouse WT hepatocytes in collagen sandwich cultures, when compared to *SEMA7A_WT* expression construct transfected cells (Fig.2G). Based on these observations, we concluded that *Sema7a*^{R145W} homozygous mutation reduced the expression of canalicular membrane Bsep and Mrp2 in hepatocytes.

Moreover, previous studies have indicated that activation of PKCs reduces canalicular membrane

expression of BSEP and MRP2 by a post-translational mechanism in cholestatic hepatocytes (Chai *et al*, 2015; Crocenzi *et al*, 2008; Kubitz *et al*, 2004; Perez *et al*, 2006). Indeed, we also observed intrahepatic cholestasis (Table.1 and Table.EV16) and the activated PKCδ/ε signaling (Fig.EV4A) in *Sema7a*^{R145W} homozygous mice, implying that *Sema7a*^{R145W} homozygous mutation might activate the PKCδ/ε signaling. This hypothesis was verified in *Sema7a*^{R145W} homozygous mouse primary hepatocytes and human HepG2 cells transfected with *SEMA7A_R148W* expression construct (Fig.EV4B&C). Therefore, we propose that *Sema7a*^{R145W} mutation reduces canalicular membrane Bsep and Mrp2 expression by activating the PKCδ/ε signaling in hepatocytes, although the details remain to be revealed. We have added these new data in the revised manuscript.

References:

Chai J, Cai SY, Liu X, Lian W, Chen S, Zhang L, Feng X, Cheng Y, He X, He Y *et al* (2015) Canalicular membrane MRP2/ABCC2 internalization is determined by Ezrin Thr567 phosphorylation in human obstructive cholestasis. *J Hepatol* 63: 1440-1448

Crocenzi FA, Sanchez Pozzi EJ, Ruiz ML, Zucchetti AE, Roma MG, Mottino AD, Vore M (2008) Ca(2+)-dependent protein kinase C isoforms are critical to estradiol 17beta-D-glucuronide-induced cholestasis in the rat. *Hepatology* 48: 1885-1895

Kubitz R, Saha N, Kuhlkamp T, Dutta S, vom Dahl S, Wettstein M, Haussinger D (2004) Ca2+-dependent protein kinase C isoforms induce cholestasis in rat liver. *J Biol Chem* 279: 10323-10330

Perez LM, Milkiewicz P, Elias E, Coleman R, Sanchez Pozzi EJ, Roma MG (2006) Oxidative stress induces internalization of the bile salt export pump, Bsep, and bile salt secretory failure in isolated rat hepatocyte couplets: a role for protein kinase C and prevention by protein kinase A. *Toxicol Sci* 91: 150-158

2. The paper seems to contradict earlier observations that Sema7A deficiency is protective against liver injury in BDL and CCL4 induced liver damage. The authors explain this apparent discrepancy by stating that the R145W variant may be a gain of function variant. This hypothesis should be further explored using a Sema7A dosing in BSEP expressing cell models and seems not to match well with a recessive mode of inheritance.

Response: We thank you for your critical comments and professional suggestions, which allowed us to improve the manuscript. We have now performed additional experiments to further test our hypothesis according to your suggestions. First, over-expression of *SEMA7A_R148W* or *SEMA7A_WT* protein remarkably reduced canalicular membrane Bsep and Mrp2 expression in a dose dependent manner in primary mouse hepatocyte collagen sandwich cultures (Fig.2G). Second, in the same dose transfection, the levels of canalicular membrane Bsep and Mrp2 protein expression were substantially lower in primary hepatocytes in sandwich cultures transfected with *SEMA7A_R148W* construct than that of with *SEMA7A_WT* construct (Fig.2G). Third, *Sema7a*^{R145W} (human R148W) homozygous mutation activated the PKCδ/ε signaling in mouse primary hepatocytes and human HepG2 cells (Fig.EV4B&C). Therefore, we speculate that the *Sema7a*^{R145W} mutation is a gain-of-function mutation. Furthermore, *Sema7a*^{R145W} homozygous mice displayed an intrahepatic cholestatic phenotype (Table.1 and EV16). Conversely, *Sema7a* deficiency in mice would protect against cholestatic liver injury, in agreement with observations seen in the *Sema7a* knockout mice. Regarding the issue of recessive inheritance, we need to collect and analyze additional patients in the future. We have added these new data and made the corresponding changes in the revised manuscript.

Minor comments

1. The liver phenotype of the Sema7a_R145W mice is different from the patients with respect to bilirubin levels. This is somewhat remarkable as hyperbilirubinemia is described for Slc10a1 deficiency (which is present in the patient). Are similar differences due to species differences and also observed with other PFIC genes?

Response: We agree that there is a difference in serum bilirubin levels between *Sema7a*^{R145W} mice and the child patient. *Slc10a1* deficiency displayed hypercholanemia but not hyperbilirubinemia or liver injury (Vaz *et al*, 2015; Liu *et al*, 2017). Previous studies have shown an elevation in serum levels of total bile acids and/or bilirubin in PFIC patients and mouse models, such as PFIC3 (Aronson *et al*, 2019; Baker *et al*, 2018). Similar to other PFIC phenotype (e.g. PFIC3), *Sema7a*^{R145W} homozygous mice also displayed the elevated levels of serum total BA and bilirubin, as well as serum ALT and AST. However, the child patient with this new PFIC exhibited the elevated levels of serum total BA, ALT and AST, but not bilirubin.

The differences may stem from species differences or the early stage of the new PFIC in this patient.

References:

Aronson S, Bakker R, Shi X, Duijst S, Ten Bloemendaal L, de Waart D, Verheij J, Ronzitti G, Oude Elferink R, Beuers U *et al* (2019) Liver-directed gene therapy results in long-term correction of progressive familial intrahepatic cholestasis type 3 in mice. J Hepatol 71: 153-162

Baker A, Kerkar N, Todorova L, Kamath BM, Houwen RHJ (2018) Systematic review of progressive familial intrahepatic cholestasis. Clin Res Hepatol Gastroenterol 43: 20-36

Liu RH, Chen CM, Xia XF, Liao QJ, Wang Q, Newcombe PJ, Xu SH, Chen MH, Ding Y, Li XY *et al* (2017) Homozygous p.Ser267Phe in SLC10A1 is associated with a new type of hypercholanemia and implications for personalized medicine. *Sci Rep* 7: 9214

Vaz FM, Paulusma CC, Huidekoper H, de Ru M, Lim C, Koster J, Ho-Mok K, Bootsma AH, Groen AK, Schaap FG *et al* (2015) Sodium taurocholate cotransporting polypeptide (SLC10A1) deficiency: conjugated hypercholanemia without a clear clinical phenotype. *Hepatology* 61: 260-267

2. The PFIC phenotype is different from PFIC1/2 in the sense that pruritus was absent. This should be discussed.

Response: Thank you for your helpful suggestion. Indeed, we did not observe pruritus in the child patient, which presented in some cases with other PFIC types, such as PFIC1-3. El-Guindi *et al* (2016) reported a 35.3% incidence of pruritus in a PFIC2 cohort of 17 patients. Therefore, additional patients need to be collected and analyzed. As suggested, we have discussed pruritus in the Discussion section.

References:

El-Guindi MA, Sira MM, Hussein MH, Ehsan NA, Elsheikh NM (2016) Hepatic immunohistochemistry of bile transporters in progressive familial intrahepatic cholestasis. Ann Hepatol 15: 222-229

3. Recent data that Sema7A is crucial for resolution of severe inflammation (PNAS 2021; PMID: 33637648) should be discussed as inflammation plays a role in cholestatic liver injury.

<u>Response</u>: Thank you for pointing this out. We have included this new reference and discussed the inflammatory role of Sema7a in the Discussion section of the revised manuscript.

Comments/concerns from Reviewer 2

Referee #2 (Comments on Novelty/Model System for Author):

In general the study is of interest but lack a mechanistic explanation of the findings.

<u>Response</u>: We thank you for your positive and critical comments. We have performed additional experiments and tried our best to provide a mechanistic explanation based on our data. Also, we modified the manuscript into a short report, because of the lack of detail mechanisms.

Referee #2 (Remarks for Author):

This study reports on the characterization of a Semaphorin 7a mutation in a female infant showing features of liver injury and cholestasis at 2 months of age. The clinical syndrome is marked by increased levels of AST and ALT and total bile acids (TBA) in the blood. Both the father and the mother had normal AST and ALT and TBA. Liver histopathology also revealed a robust hydropic degeneration in hepatocytes and increased accumulation of conjugated BA in the liver, while immunohistochemistry in mice demonstrated an internalization of canalicular membrane BSEP and MRP2.

Gene sequencing revealed a mutation in the semaphoring 7a gene. The newly identified mutation is a homozygous p.R148W mutation in SEMA7A, and SEMA7A R145W homozygous mice, displayed similar histopathology features than the patients, including a hydropic degeneration of hepatocytes and increased accumulation of intrahepatic BA.

The data suggest that SEMA7AR148W homozygous mutation is a new genetic determinant of cholestatic liver injury.

The data reported are of interest.

<u>Response</u>: Thank you for your positive comments.

My comments are the detailed here:

1. The first sentence of Discussion is an over-interpretation of the results: "Here, we first reported a case of familial cholestasis caused by a homozygous p.R148W mutation in SEMA7A, and provided evidence of its pathophysiologic mechanism. This new genetic cholestatic disorder is characterized by (1) elevated levels of serum ALT, AST, and TBA (Fig.2A-C and Tables.3&S13); (2) striking hydropic degeneration in hepatocytes and increased accumulation of conjugated BA in the liver (Fig.2D&E and Table.4); and (3) internalization of canalicular membrane BSEP and MRP2 (Figs.3D&E". The authors have no data on the histopathology of the patient described in this report, therefore there is no information on point 2 and 3. This need to be amended and the same should be done in the abstract and throughout the manuscript.

Response: We agree with the reviewer and appreciate your suggestions. Accordingly, we have removed the point 2 and 3, and made the corresponding changes in the manuscript.

2. Also the fact that the patient has a homozygous p.S267F mutation in SLC10A1, which leads to very close clinical pattern, does not allow a clear separation between the two syndromes. This suggest a cautionary approach on the title and abstract. The abstract should inform the readers that the patients had both mutations.

Response: We appreciate your professional advice. We have made changed the Abstract accordingly.

3. Page 16, the human mutation SEMA7A R148W is described as a "gain of function" mutation. The patient harboring this mutation had cholestasis and therefore the mutation should lead to some degree of loss of function. Particularly if the SEMA7A R148W prevents insertion of BSEP and MRP2 in the canalicular membrane of hepatocytes. Please clarify.

Response: We have studied your thoughtful comments carefully. After performing additional experiments, our data supported that *SEMA7A*$^{\mathbf{R148W}}$ may be a “gain of function” mutation as addressed the question 2 from Reviewer #1. Briefly, over-expression of *SEMA7A_R148W* or *SEMA7A_WT* protein markedly decreased canalicular Bsep and Mrp2 proteins in primary mouse hepatocytes in sandwich

cultures in a dose-dependent manner, particularly after over-expression of the mutant (Fig.2G). Cell culture experiments for *Sema7a*^{R145W} primary mouse hepatocytes and human HepG2 cells transfected with *SEMA7A*_R148W construct revealed that this mutation also increased PKCδ/ε activities (Fig.EV4B&C). Furthermore, *Sema7a*^{R145W} homozygous mice displayed an intrahepatic cholestatic phenotype with reduction in the levels of canalicular membrane Bsep and Mrp2 expression (Fig.2D, Table.1, and Table EV16). Therefore, the gain-of-function mutation of *Sema7a*^{R145W} may cause cholestasis by repressing of hepatic Bsep and Mrp2 expression. We have added these new data and discussed these points in the revised manuscript.

4. A previous study has shown that lack of semaforin7a is protective again cholestasis in mice, which is in contrast with the data shown in this paper. Refence made to this paper in page 15 is wrong since the paper is cited in ref. 7 (not 6).

<u>Response</u>: Thank you for pointing out the wrong citations, which has been corrected in the revised manuscript. As described above, we speculate that *Sema7a*^{R145W} homozygous mutation is a gain-of-function mutation and causes intrahepatic cholestasis in mice. This would be consistent with that *Sema7a* deficiency protects mice against cholestatic liver injury.

Furthermore, this paper has been criticized because extensive image duplications: https://pubpeer.com/publications/089AD44F94ECA4B33A4BF1718F644E. I will suggest to remove it from reference.

<u>Response</u>: We appreciate your advice. Now, we have removed this reference in the revised manuscript.

5. One limitation of the study is the lack of a mechanism explaining how SEMA7A regulates BSEP insertion at the canalicular membrane. The structure shown in Figure 1D-F is poorly informative.

<u>Response</u>: We have performed additional experiments to explore the preliminary mechanisms. We found that *Sema7a*^{R145W} mutation markedly reduced the levels of canalicular membrane Bsep and Mrp2 expression in primary mouse hepatocytes in sandwich cultures (Fig.2F), and increased PKCδ/ε activities

in mouse and human hepatocytes (Fig.EV4B&C). Moreover, many studies have demonstrated that PKC activation reduces canalicular Bsep and Mrp2 proteins in cholestatic hepatocytes (Chai *et al*, 2015; Crocenzi *et al*, 2008; Kubitz *et al*, 2004; Perez *et al*, 2006). Therefore, we speculate that *Sema7a*^{R145W} homozygous mutation may reduce canalicular membrane Bsep and Mrp2 expression by increasing PKCδ/ε activities in hepatocytes. Accordingly, we have added these new data in the revised manuscript. Also, we modified the manuscript into a short report, because of the lack of detail mechanisms.

References:

Chai J, Cai SY, Liu X, Lian W, Chen S, Zhang L, Feng X, Cheng Y, He X, He Y *et al* (2015) Canalicular membrane MRP2/ABCC2 internalization is determined by Ezrin Thr567 phosphorylation in human obstructive cholestasis. *J Hepatol* 63: 1440-1448

Crocenzi FA, Sanchez Pozzi EJ, Ruiz ML, Zucchetti AE, Roma MG, Mottino AD, Vore M (2008) Ca(2+)-dependent protein kinase C isoforms are critical to estradiol 17beta-D-glucuronide-induced cholestasis in the rat. *Hepatology* 48: 1885-1895

Kubitz R, Saha N, Kuhlkamp T, Dutta S, vom Dahl S, Wettstein M, Haussinger D (2004) Ca2+-dependent protein kinase C isoforms induce cholestasis in rat liver. *J Biol Chem* 279: 10323-10330

Perez LM, Milkiewicz P, Elias E, Coleman R, Sanchez Pozzi EJ, Roma MG (2006) Oxidative stress induces internalization of the bile salt export pump, Bsep, and bile salt secretory failure in isolated rat hepatocyte couplets: a role for protein kinase C and prevention by protein kinase A. *Toxicol Sci* 91: 150-158

6. Table 4 shows a disproportionate increase of TCDCA over TMCA, suggesting an impaired conversion of CDCA to MCA. Does the authors have data on Cyp2c70?

Response: Thank you for the constructive suggestion. We have now determined Cyp2c70 mRNA expression in mouse livers and found a significant decrease in in homozygous mice, when compared to WT and heterozygous mice (Fig.2C). We have added this new data in the revised manuscript.

7. How the authors explain differential regulation of Ost and Mrp4 in figure 2

Response: After reading the question, we carefully examined the original data about hepatic bile acid transporters. We noticed that there was an outlier in the heterozygous group that had affected the qPCR data of Ostβ and Mrp4 in the former figure 2. To address this issue, we repeated these experiments and corrected these results in the revised manuscript.

Responses to the Reviewer 3

Referee #3 (Comments on Novelty/Model System for Author):

We constantly learn about novel mutations. Many of these are variants of unknown significance. The locus the authors describe is interesting but they cannot convincingly correlate the in vitro and in silico findings to a human clinical phenotype.

Response: Thank you for your insightful and critical comments. We have added new clinical data as below and discussed this issue in the revised manuscript.

Referee #3 (Remarks for Author):

This is potentially an interesting finding however I see several drawbacks that should be addressed before publication:

1. There is no convincing correlation of the in vitro and in silico findings to the clinical phenotype in the child. Histopathology and longitudinal clinical data is missing in particular as previous publication on this gene suggested alleviation of cholestasis rather than a PFIC phenotype.

Response: We greatly appreciate your critical comments and helpful suggestions. To address the concern, we performed additional experiments and collected new clinical data. Firstly, the child patient carried with a *SEMA7A*^{R148W} homozygous mutation and displayed the elevated levels of serum ALT, AST and TBA (Table EV1). Secondly, *Sema7a*^{R145W} homozygous mice also exhibited the elevated levels of serum ALT, AST and TBA (Table.1 and Fig.EV3) and intrahepatic cholestasis (Table.1), which recapitulated her clinical phenotypes. Thirdly, over-expression of *SEMA7A_R148W* protein in primary

mouse hepatocytes in sandwich cultures remarkably reduced the levels of canalicular membrane Bsep and Mrp2 expression in a dose-dependent manner (Fig.2G), compared to that of with *SEMA7A_WT* over-expression (Fig.2G). Furthermore, *Sema7a*^{R145W} mutation increased PKCδ/ε activities in mouse and human hepatocytes (Fig.EV4B&C), supporting that the *Sema7a*^{R145W} mutation is a gain-of-function mutation. Collectively, the *Sema7a*^{R145W} mutation is gain-of-function mutation and can cause intrahepatic cholestasis in mice. Conversely, *Sema7a* deficiency in mice should attenuate cholestasis.

We did not have histopathological data of the child patient because the patient health condition did not meet the requirements for liver biopsy. However, longitudinal clinical data were collected and added in the revised manuscript (Fig.3). After finding abnormal liver function in the child, the child was treated with a therapeutic drug for cholestasis, UDCA (13 mg/kg/d) and a dietary supplement GSH (40 mg/kg/d). Interestingly, treatment with both UDCA and GSH for 2 weeks significantly corrected the abnormal levels of serum ALT and AST and reduced the levels of serum TBA in the patient (Fig.3). However, when the treatment was ceased, the levels of serum ALT, AST and TBA increased abnormally in the patient (Fig.3). These new clinical data supported that *Sema7a*^{R148W} mutation caused intrahepatic cholestasis in the child patient. Accordingly, we have added these new data and discussed them in the revised manuscript.

2. Suggest you follow the reporting guidelines with (equator network)

Response: We appreciate your advice. We have revised the manuscript following the CARE case report guidelines. The checking list was attached to the revised manuscript.

3. The language needs revision.

Response: We appreciate your advice. Now, we have carefully checked each sentence to eliminate/reduce any potential syntax. The manuscript has been proof-read by two native English biologists.

30th Aug 2021

Dear Prof. Chai,

Thank you for the submission of your revised manuscript to EMBO Molecular Medicine. I am pleased to inform you that we will be able to accept your manuscript pending the following final amendments:

1) Please address all the points raised by the referees. Particular attention should be given to the western blots in Figure 2D that appear not to be form the same gel although single GAPDH is presented as a loading control as pointed out by the referee #2. Western blots from different gels should be presented with a corresponding loading control blot.
2) In the main manuscript file, please do the following:
- Correct/answer the track changes suggested by our data editors by working from the attached document.
- Reduce keywords to max. 5.
- Remove the "Abbreviation list". Abbreviations should be incorporated in the text.
- Remove data not shown (p. 8 and 17).
- Add callout for Fig 1A. Also, Fig EV2 should be called out before Fig EV4.
- In M&M, a statistical paragraph that should reflect all information that you have filled in the Authors Checklist, especially regarding randomization, blinding, replication.
- Use initials for author contributions and move it down after "Acknowledgements".
- Move conflict of interest statement down after "Author contributions".
- Merge "Grants support" with "Acknowledgements".
- Add full URL to your deposited data and please be aware that all deposited data have to be freely available before publication of the manuscript. Please use the following format to report the accession number of your data:

The datasets produced in this study are available in the following databases:
[data type]: [full name of the resource] [accession number/identifier] ([doi or URL or identifiers.org/DATABASE:ACCESSION])

Please check "Author Guidelines" for more information.
https://www.embopress.org/page/journal/17574684/authorguide#availabilityofpublishedmaterial
3) EV Tables: Please move all the EV Tables to "Appendix", rename them to "Appendix Table S1" etc., and update their callouts in the main text.
4) Source data: Please upload one file per figure for the main Figures (zipp were appropriate) and zipp source data for all EV Figures as one file.
5) CARE Checklist is not required and can be removed.
6) As part of the EMBO Publications transparent editorial process initiative (see our Editorial at http://embomolmed.embopress.org/content/2/9/329), EMBO Molecular Medicine will publish online a Review Process File (RPF) to accompany accepted manuscripts. This file will be published in conjunction with your paper and will include the anonymous referee reports, your point-by-point response and all pertinent correspondence relating to the manuscript. Let us know whether you agree with the publication of the RPF and as here, if you want to remove or not any figures from it prior to publication. Please note that the Authors checklist will be published at the end of the RPF.
7) Please provide a point-by-point letter INCLUDING my comments as well as the reviewer's reports and your detailed responses (as Word file).

I look forward to reading a new revised version of your manuscript as soon as possible.

Yours sincerely,

Zeljko Durdevic

***** Reviewer's comments *****

Referee #1 (Comments on Novelty/Model System for Author):

They addressed my comments adequately and I have no further comments.

Referee #1 (Remarks for Author):

The authors have adapted the manuscript to a short report. This better reflects the level of mechanistical insight linking Sem7A variant to cholestasis. Furthermore, they did a nice job in assesssing the direct effects of SEM7A overexpression (WT and patient variant) on BSEP/MRP2 abundance/localization. I would have prefered if the experiments with SEMA7A overexpression were performed and analyzed in a blinded (and more quantitative) manner but don't consider this essential for this manuscript at this stage.
The text is also improved and at present accurately reflects the data. I have no further comments

Referee #2 (Remarks for Author):

The authors have provided convincing replies to my comments. I have a technical comment related to the quality of WB that is very low and should be increased. Further on, I'm concerned over figure 3D, since many proteins are presented with a single Gapdh as a control. This will imply that the same gell has been used multiple times, but the morphoplogy of the various bands clearly indicate that is not the case. As such, relative control for each protein or groups of protein should be provided.

The authors performed the requested editorial changes.

Responses to the Reviewer

Referee #1 (Comments on Novelty/Model System for Author):

They addressed my comments adequately and I have no further comments.

Response: We appreciate his/her support.

Referee #1 (Remarks for Author):

The authors have adapted the manuscript to a short report. This better reflects the level of mechanistical insight linking Sem7A variant to cholestasis. Furthermore, they did a nice job in assesssing the direct effects of SEM7A overexpression (WT and patient variant) on BSEP/MRP2 abundance/localization. I would have prefered if the experiments with SEMA7A overexpression were performed and analyzed in a blinded (and more quantitative) manner but don't consider this essential for this manuscript at this stage. The text is also improved and at present accurately reflects the data. I have no further comments.

Response: We thank his/her positive comments and professional suggestions, which will help improve our project in the future.

Referee #2 (Remarks for Author):

The authors have provided convincing replies to my comments. I have a technical comment related to the quality of WB that is very low and should be increased.

Response: We thank his/her positive comments. We agree that some Western blot data may be at low quality, such as Ntcp and Cyp7a1 in Figure 2D. Indeed, we repeated the experiments for multiple times. However, we did not obtain a high quality blot, which may stem from low quality of primary antibodies available in our market.

Further on, I'm concerned over figure 2D, since many proteins are presented with a single Gapdh as a control. This will imply that the same gell has been used multiple times, but the morphoplogy of the various bands clearly indicate that is not the case. As such, relative control for each protein or groups of protein should be provided.

Response: We appreciate his/her advice. Accordingly, we have added the corresponding loading controls in the revised Figure 2D.

7th Sep 2021

Dear Prof. Chai,

We are pleased to inform you that your manuscript is accepted for publication and is now being sent to our publisher to be included in the next available issue of EMBO Molecular Medicine.

EMBO PRESS

YOU MUST COMPLETE ALL CELLS WITH A PINK BACKGROUND ↓

PLEASE NOTE THAT THIS CHECKLIST WILL BE PUBLISHED ALONGSIDE YOUR PAPER

Corresponding Author Name: Jin Chai
Journal Submitted to: EMBO Molecular Medicine
Manuscript Number: EMM-2021-14563

Reporting Checklist For Life Sciences Articles (Rev. June 2017)

This checklist is used to ensure good reporting standards and to improve the reproducibility of published results. These guidelines are consistent with the Principles and Guidelines for Reporting Preclinical Research issued by the NIH in 2014. Please follow the journal's authorship guidelines in preparing your manuscript.

A- Figures
1. Data
The data shown in figures should satisfy the following conditions:

- the data were obtained and processed according to the field's best practice and are presented to reflect the results of the experiments in an accurate and unbiased manner.
- figure panels include only data points, measurements or observations that can be compared to each other in a scientifically meaningful way.
- graphs include clearly labeled error bars for independent experiments and sample sizes. Unless justified, error bars should not be shown for technical replicates.
- if n< 5, the individual data points from each experiment should be plotted and any statistical test employed should be justified
- Source Data should be included to report the data underlying graphs. Please follow the guidelines set out in the author ship guidelines on Data Presentation.

2. Captions

Each figure caption should contain the following information, for each panel where they are relevant:

- a specification of the experimental system investigated (eg cell line, species name).
- the assay(s) and method(s) used to carry out the reported observations and measurements
- an explicit mention of the biological and chemical entity(ies) that are being measured.
- an explicit mention of the biological and chemical entity(ies) that are altered/varied/perturbed in a controlled manner.

- the exact sample size (n) for each experimental group/condition, given as a number, not a range;
- a description of the sample collection allowing the reader to understand whether the samples represent technical or biological replicates (including how many animals, litters, cultures, etc.).
- a statement of how many times the experiment shown was independently replicated in the laboratory.
- definitions of statistical methods and measures:
  - common tests, such as t-test (please specify whether paired vs. unpaired), simple χ2 tests, Wilcoxon and Mann-Whitney tests, can be unambiguously identified by name only, but more complex techniques should be described in the methods section;
  - are tests one-sided or two-sided?
  - are there adjustments for multiple comparisons?
  - exact statistical test results, e.g., P values = x but not P values < x;
  - definition of 'center values' as median or average;
  - definition of error bars as s.d. or s.e.m.

Any descriptions too long for the figure legend should be included in the methods section and/or with the source data.

In the pink boxes below, please ensure that the answers to the following questions are reported in the manuscript itself. Every question should be answered. If the question is not relevant to your research, please write NA (non applicable). We encourage you to include a specific subsection in the methods section for statistics, reagents, animal models and human subjects.

USEFUL LINKS FOR COMPLETING THIS FORM

http://www.antibodypedia.com
http://1degreebio.org
http://www.equator-network.org/reporting-guidelines/improving-bioscience-research-repor
http://grants.nih.gov/grants/olaw/olaw.htm
http://www.mrc.ac.uk/Ourresearch/Ethicsresearchguidance/Useofanimals/index.htm
http://ClinicalTrials.gov
http://www.consort-statement.org
http://www.consort-statement.org/checklists/view/32-consort/66-title
http://www.equator-network.org/reporting-guidelines/reporting-recommendations-for-tumo
http://datadryad.org
http://figshare.com
http://www.ncbi.nlm.nih.gov/gap
http://www.ebi.ac.uk/ega
http://biomodels.net/
http://biomodels.net/miriam/
http://jjj.biochem.sun.ac.za
https://osp.od.nih.gov/biosafety-biosecurity-and-emerging-biotechnology/
http://www.selectagents.gov/

B- Statistics and general methods

Please fill out these boxes ↓ (Do not worry if you cannot see all your text once you press return)

1.a. How was the sample size chosen to ensure adequate power to detect a pre-specified effect size?	Sample sizes were carefully determined according to previously published of ours and other groups.
1.b. For animal studies, include a statement about sample size estimate even if no statistical methods were used.	The sample sizes are similar to previous reports in the same field.
2. Describe inclusion/exclusion criteria if samples or animals were excluded from the analysis. Were the criteria pre-established?	No data were excluded from the analyses.
3. Were any steps taken to minimize the effects of subjective bias when allocating animals/samples to treatment (e.g. randomization procedure)? If yes, please describe.	All samples and animals used in this study were randomized into experimental groups.
For animal studies, include a statement about randomization even if no randomization was used.	All samples and animals used in this study were randomized into experimental groups. In vivo studies, all mice were age- and sex- matched and randomly assigned into control and treatment groups to ensure similar body weight.
4.a. Were any steps taken to minimize the effects of subjective bias during group allocation or/and when assessing results (e.g. blinding of the investigator)? If yes please describe.	In immunohistochemistry and multiplex immunofluorescence , images were acquired using the same laser power and sensitivity, and image processing was the same across the experimental groups.
4.b. For animal studies, include a statement about blinding even if no blinding was done	NA.
5. For every figure, are statistical tests justified as appropriate?	Yes.
Do the data meet the assumptions of the tests (e.g., normal distribution)? Describe any methods used to assess it.	The statistical methods are indicated in the figure legends.
Is there an estimate of variation within each group of data?	Yes.

Is the variance similar between the groups that are being statistically compared?	If the variance was not similar,we performed the unpaired test with Welch's correction for data analysis.

C- Reagents

6. To show that antibodies were profiled for use in the system under study (assay and species), provide a citation, catalog number and/or clone number, supplementary information or reference to an antibody validation profile. e.g., Antibodypedia (see link list at top right), 1DegreeBio (see link list at top right).	Related information was described in Appendix Table S18.
7. Identify the source of cell lines and report if they were recently authenticated (e.g., by STR profiling) and tested for mycoplasma contamination.	Human hepatoma HepG2 cells (ATCC, Manassas, VA) were kindly provided by Prof. Cheng Qian (Southwest Cancer Center, Southwest Hospital, Third Military Medical University, Chongqing, China) and identified by STR.

* for all hyperlinks, please see the table at the top right of the document

D- Animal Models

8. Report species, strain, gender, age of animals and genetic modification status where applicable. Please detail housing and husbandry conditions and the source of animals.	Strain, gender, age, genetic modification and source of mice were described in Materials and Methods section.Mice were kept in an animal room under SPF conditions(temperature: 18 - 23℃; humidity: 40–60%; photoperiod: 12 h light/12 h dark) with free access to food and water.
9. For experiments involving live vertebrates, include a statement of compliance with ethical regulations and identify the committee(s) approving the experiments.	Animal studies were conducted under the approval of the Unicersity of Third Military Medical University Committee on Use and Care of Animals.
10. We recommend consulting the ARRIVE guidelines (see link list at top right) (PLoS Biol. 8(6), e1000412, 2010) to ensure that other relevant aspects of animal studies are adequately reported. See author guidelines, under 'Reporting Guidelines'. See also: NIH (see link list at top right) and MRC (see link list at top right) recommendations. Please confirm compliance.	All animal studies comply with the ARRIVE guidelines.

E- Human Subjects

11. Identify the committee(s) approving the study protocol.	The study protocol was reviewed and approved by the Institutional Ethics Review Board of the Southwest Hospital, Chongqing China. Protocol number:KY2020169
12. Include a statement confirming that informed consent was obtained from all subjects and that the experiments conformed to the principles set out in the WMA Declaration of Helsinki and the Department of Health and Human Services Belmont Report.	All subjects obtained and signed the informed consent and that the experiments conformed to the principles set out in the WMA Declaration of Helsinki and the Department of Health and Human Services Belmont Report.
13. For publication of patient photos, include a statement confirming that consent to publish was obtained.	A consent that agree to publish patient photos was signed by patient'parents and was included in the study.
14. Report any restrictions on the availability (and/or on the use) of human data or samples.	NA.
15. Report the clinical trial registration number (at ClinicalTrials.gov or equivalent), where applicable.	NA.
16. For phase II and III randomized controlled trials, please refer to the CONSORT flow diagram (see link list at top right) and submit the CONSORT checklist (see link list at top right) with your submission. See author guidelines, under 'Reporting Guidelines'. Please confirm you have submitted this list.	NA.
17. For tumor marker prognostic studies, we recommend that you follow the REMARK reporting guidelines (see link list at top right). See author guidelines, under 'Reporting Guidelines'. Please confirm you have followed these guidelines.	NA.

F- Data Accessibility

18: Provide a "Data Availability" section at the end of the Materials & Methods, listing the accession codes for data generated in this study and deposited in a public database (e.g. RNA-Seq data: Gene Expression Omnibus GSE39462, Proteomics data: PRIDE PXD000208 etc.) Please refer to our author guidelines for 'Data Deposition'.<br><br>Data deposition in a public repository is mandatory for:<br>a. Protein, DNA and RNA sequences<br>b. Macromolecular structures<br>c. Crystallographic data for small molecules<br>d. Functional genomics data<br>e. Proteomics and molecular interactions	The deposition of Genome-wide sequencing data was described in the "Data Availability" section.The datasets produced in this study are available in the following databases:WGS data: BioProject-accession number PRJNA487655 (https://www.ncbi.nlm.nih.gov/bioproject/?term=PRJNA487655).
19. Deposition is strongly recommended for any datasets that are central and integral to the study; please consider the journal's data policy. If no structured public repository exists for a given data type, we encourage the provision of datasets in the manuscript as a Supplementary Document (see author guidelines under 'Expanded View' or in unstructured repositories such as Dryad (see link list at top right) or Figshare (see link list at top right).	NA.
20. Access to human clinical and genomic datasets should be provided with as few restrictions as possible while respecting ethical obligations to the patients and relevant medical and legal issues. If practically possible and compatible with the individual consent agreement used in the study, such data should be deposited in one of the major public access-controlled repositories such as dbGAP (see link list at top right) or EGA (see link list at top right).	NA.
21. Computational models that are central and integral to a study should be shared without restrictions and provided in a machine-readable form. The relevant accession numbers or links should be provided. When possible, standardized format (SBML, CellML) should be used instead of scripts (e.g. MATLAB). Authors are strongly encouraged to follow the MIRIAM guidelines (see link list at top right) and deposit their model in a public database such as Biomodels (see link list at top right) or JWS Online (see link list at top right). If computer source code is provided with the paper, it should be deposited in a public repository or included in supplementary information.	NA.

G- Dual use research of concern

22. Could your study fall under dual use research restrictions? Please check biosecurity documents (see link list at top right) and list of select agents and toxins (APHIS/CDC) (see link list at top right). According to our biosecurity guidelines, provide a statement only if it could.	NA.
